# VTA dopamine neuron activity produces spatially organized value representations

**DOI:** 10.1101/2025.11.04.685995

**Published:** 2025-11-06

**Authors:** Alejandro Pan-Vazquez, Christopher A. Zimmerman, Brenna McMannon, Julie M. J. Fabre, Miranta Louka, Tingying Jia, Yotam Sagiv, Steven J. West, Mayo Faulkner, International Brain Laboratory, Peter Dayan, Ilana B. Witten

**Affiliations:** 1 Princeton Neuroscience Institute, Princeton University, Princeton, NJ, USA; 2 Sainsbury Wellcome Centre, University College London, London, UK; 3 Institute of Neurology, University College London, London, UK; 4 Max Planck Institute for Biological Cybernetics, Tübingen, Germany; 5 University of Tübingen,Tübingen, Germany; 6 Howard Hughes Medical Institute, Princeton University, Princeton, NJ, USA

## Abstract

How does the activity of midbrain dopamine (DA) neurons reinforce actions? A prominent hypothesis is that the activity of ventral tegmental area (VTA) DA neurons instructs representations of predicted reward, or value, in downstream neurons^1^. To directly test this model, we performed comprehensive striatal recordings in mice engaged in a trial-and-error probabilistic learning task where they continuously adapted their choices to obtain a reward of optogenetic stimulation of VTA DA neurons (paired with an auditory cue). We then assessed neural representations of action values (estimated from a behavioral model), revealing for the first time that VTA DA stimulation is sufficient to generate downstream neural correlates of action value. Surprisingly, these value correlates were strongest in the intermediate caudoputamen (CP) and weakest in the nucleus accumbens (NAc), despite NAc being the major projection target of VTA DA neurons^2,3^. This was true not only for the value of each choice, but also for state value (reward expectation) and relative value (the decision variable). However, value representations were differentially organized within the intermediate CP, with ventromedial domains (which receive inputs from orbitofrontal cortex) preferentially encoding state value and dorsolateral domains (which receive inputs from motor cortex) preferentially encoding relative value. A difference in learning rate for the value computation between NAc and CP did not explain the relatively weak value correlates in NAc. Instead, we found that VTA DA stimulation was sufficient to produce learned neural responses to the stimulation-paired auditory cue throughout the striatum, including in the NAc, and that animals work for this cue rather than for VTA DA stimulation itself. Overall, this suggests that VTA DA neurons support trial-and-error learning indirectly, by making stimuli valuable (“conditioned reinforcers”), which in turn support the generation of action value representations in the CP.

## INTRODUCTION

How do animals make choices that allow them to obtain rewards? DA neurons are thought to contribute critically to this process by encoding an error signal for predicting rewards^4–7^. This error signal is hypothesized to instruct representations in downstream target neurons of the rewards predicted by different stimuli or actions (i.e., values), which animals can then use to make the most appropriate or valuable choice^1,8,9^.

There is extensive support for aspects of this model. Behaviorally, optogenetic stimulation of VTA DA neurons following an action can reinforce it^10–15^, which implies that VTA DA neurons can make actions valuable. Concomitantly, inhibiting these neurons can rob natural rewards of their reinforcing power^16–18^. More mechanistically, DA can generate synaptic plasticity at target sites in the striatum^19–23^ and elsewhere^24–26^ across vertebrates^27^ and invertebrates^28^, which is consistent with DA potentially acting as a teaching signal that is used to sculpt downstream representations of action value.

However, the core idea that DA release produces action value representations in downstream neurons remains untested. This is because most previous work involving artificial activation of DA neurons has employed behavioral rather than neural readouts of value^11,29–36^. Conversely, previous work identifying value representations during behavior has not employed the direct activation of DA neurons^35,37–55^. In fact, despite the important characterizations of the activity patterns of DA neurons^4,7,17,36,56–81^, how such activity in turn alters downstream neural dynamics to mediate adaptive behavior remains an open question.

Thus, here we ask: is VTA DA neuron activity sufficient to produce downstream value correlates? And, if so, are they localized to the target sites of the VTA DA neurons, as might be expected if DA serves as a teaching signal for producing such representations?

## RESULTS

### Mice adjust their behavior using trial-and-error learning to obtain VTA DA stimulation

**Extended Data Figure 1.**
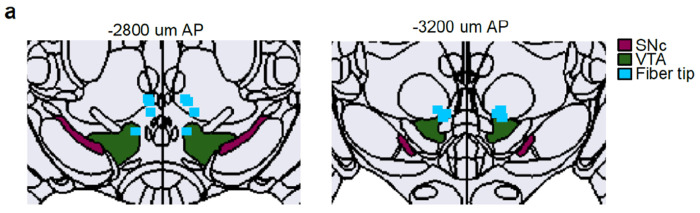
VTA stimulation histology. **a,** Histological reconstruction of optogenetic fiber tip locations (blue rectangles) for VTA DA stimulation projected to the nearest 400-μm slice of the Allen CCF (all mice in Figs. 1–4).

**Figure 1.**
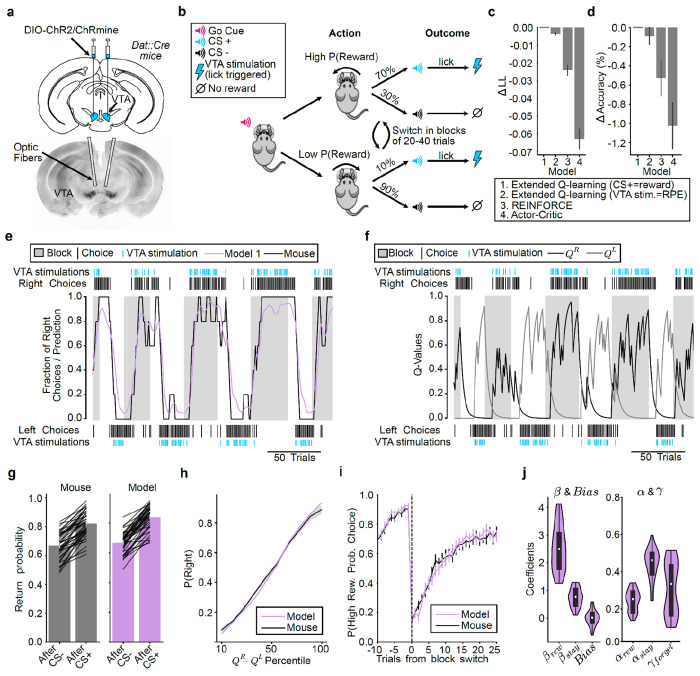
Mice adjust their behavior based on trial-and-error to obtain VTA DA stimulation. **a,** *Top:* Experimental strategy for optogenetic stimulation of VTA DA neurons. *Bottom:* Representative coronal brain slice showing virus expression and optical fiber placement. **b,** Probabilistic reversal learning task. VTA stimulation consisted of a 20 Hz burst of 5 ms light pulses for 1 s (ChR2: 447 nm, ~8 mW; ChRmine: 532 nm, ~0.5 mW). **c,** Mean accuracy of different RL models in relation to the best performing extended Q-learning model (Model 1, Extended Q-learning (CS+ as a reward), 76.8% accuracy, −0.475 LL). Error bars represent mean ± s.e.m. across sessions (n=41 sessions). **d,** Same as c, but for log-likelihood (LL) per choice. **e,** Data and Q-learning model predictions in a representative behavioral session. The fraction of right choices (black line, 5-trial moving average) and the model’s predicted probability of choosing right (purple line, 5-trial moving average) are each plotted. Shaded grey areas indicate blocks of trials where the right choice had the higher probability of reward. Black vertical ticks at the top and bottom represent individual right and left choices. Blue vertical ticks indicate VTA DA stimulation. **f**, The Q-learning model’s action value estimates for the left ($Q^{L}$) and right ($Q^{R}$) choices over the same session as in (**e**). **g,** Calibration of the Q-learning model’s win-stay behavior. Bar plot showing the probability of repeating a choice following an CS+ or CS− trial for the mice (grey) and for the Q-learning model’s on-policy simulations (purple). Error bars represent the mean ± s.e.m. across sessions. **h,** Calibration of the Q-learning model’s action selection policy. The probability of choosing the right action is plotted as a function of the percentile of the difference between the model-derived action values ($Q^{R}-Q^{L}$) for the mice (grey) and for the Q-learning model’s on-policy simulations (purple). Lines and shading represent the mean ± s.e.m. across sessions. **i,** Calibration of the Q-learning model’s reward-contingency reversal (block switch) behavior. The probability of choosing the right action is plotted as a function of trials after block switches for the mice (grey) and for the Q-learning model’s on-policy simulations (purple). Lines and error bars represent the mean ± s.e.m. across sessions. **j,** Distributions of the best-fit parameters for the Q-learning model across all sessions. $\beta_{\mathrm{Reward}}$: weight of the reward-seeking component of the decision, $\beta_{\mathrm{Stay}}$: weight of the perseveration component of the decision, *Bias*: choice bias for left or right actions, $\alpha_{\mathrm{Reward}}$: learning rate for the reward-seeking component of the chosen action, $\alpha_{\mathrm{Stay}}$: learning rate for the perseveration component, $\gamma_{\mathrm{forget}}$: forgetting rate for the reward-seeking component of the unchosen action. Violin plots illustrate the distribution of the mean of each parameter across sessions; the limits of the violin represent the extreme of the fit parameters, and the boxplot within the violin marking the median (white) and interquartile range (IQR; thick black line). *n* = 41 sessions from 8 animals for all panels. In panels **e**-**j**, results are from the best performing model: Model 1, Q-learning (CS+ as a reward)).

To address these questions, we developed a head-fixed probabilistic reversal learning task in which, rather than working for a palatable reward^15,17,82,83^, mice work to receive optogenetic stimulation of VTA DA neurons (Fig. 1a,b and Extended Data Fig. 1a). After an auditory “go cue”, the mice rotated a wheel positioned under their forepaws left or right. This choice resulted in the presentation of one of two auditory cues (CS+ or CS−). The CS+ signaled the opportunity to lick a spout to receive a 1 s burst of VTA DA stimulation. The CS− signalled the absence of stimulation. For a block of trials, turning the wheel in one direction led to the CS+ with a probability of 70%, whereas the other direction led to the CS+ with only a 10% probability. After a variable number of trials, and without any indication to the mouse, the outcome contingencies reversed so that the other choice led to the CS+ with higher probability.

**Extended Data Figure 2.**
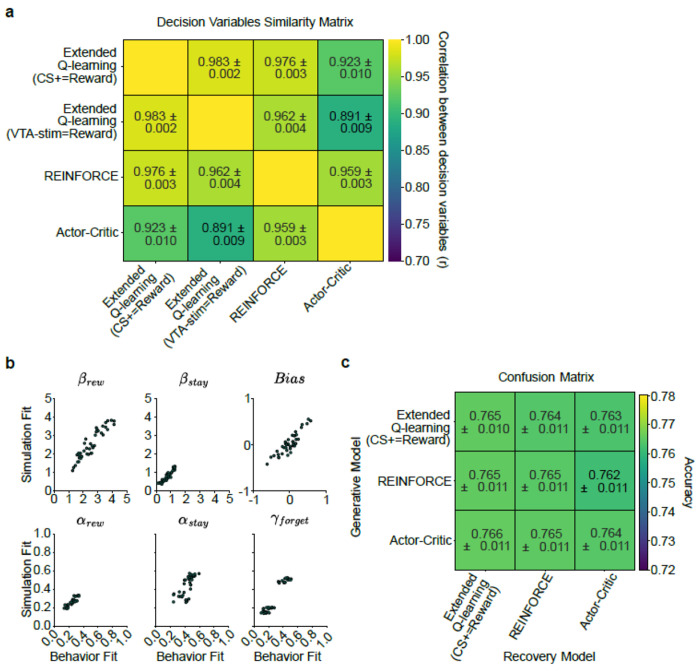
The Q-learning model parameters are recoverable, but the model’s behavior is indistinguishable from that of REINFORCE and actor-critic models. **a,** Similarity between the decision variables from every model: Relative value (*Q^R^ - Q^L^*) for Extended Q-learning models, and propensities (π) for REINFORCE and the Actor-Critic models (see Behavioral Models section in Methods for details on how these variables are computed). The cell values and color represent the mean ± s.e.m. correlation pearson correlation coefficient (r) across sessions. **b,** Parameter recovery for the best performing Q-learning model (Q-learning (CS+ as a reward)). The x-axis represents the Q-learning model’s fitted parameters from the animals’ choice data, which was then used to generate new simulated choice data on-policy. The y-axis represents the estimates of those same parameters from fitting the Q-learning model to the simulated choice data. Each point represents one simulated session (n=41 sessions). **c,** Confusion matrix characterizing the distinguishability of the Q-learning, REINFORCE, and actor-critic models. The cell values and color represent the mean ± s.e.m. accuracy of the recovered fit (% correct) across sessions. The y-axis indicates which model was used to simulate the choice data, and the x-axis indicates which model was used to recover that simulated choice data. For all plots n = 41 sessions.

The task was optimized to produce ongoing value-based adaptation of choice behavior based on recent choices and their outcomes, but not to distinguish among the diverse family of reinforcement learning models^84^. To capture the animals’ decision-making strategy, we fit paradigmatic value-based (variants of Q-learning) and policy-based (REINFORCE and actor-critic) models to the choices (Fig. 1c,d). To be comprehensive, for Q-learning we considered a version where the CS+ acted as the reward (given that it signaled the availability of VTA DA stimulation^85^), as well as a version where the VTA DA stimulation was the reward prediction error (given that DA is thought to signal a reward prediction error^1,4^). All the models fit similarly (Fig. 1c,d), produced similar trial-by-trial decision variables (Extended Data Fig. 2a), and, as expected, could not be distinguished from each other in a recovery analysis (Extended Data Fig. 2b,c). Therefore, for convenience, for subsequent analyses we used the single model which numerically fit best (Model 1: Extended Q-learning model (CS+ as reward)).

The model, which estimates the value of both the left ($Q^{L}$) and right ($Q^{R}$) actions to arrive at a choice, was able to accurately predict the animals’ choice trajectories across blocks (Fig. 1e,f). This model also successfully recapitulated key aspects of the animals’ decision-making behavior when run in simulation (“on-policy”), including the higher return probability after rewarded versus unrewarded choices (Fig. 1g), the relationship between relative value ($\Delta Q=Q^{R}-Q^{L}$) and choice probability (*P*(Right); Fig. 1h), and the gradual reversal in choice following block switches (Fig. 1i).

The fitted model coefficients revealed that the animals primarily relied on the reward ($\beta_{\mathrm{rew}}$), rather than perseveration ($\beta_{\mathrm{stay}}$) or bias, to select their choice (Fig. 1j). Moreover, the animals integrated rewards over a number of trials, as evidenced by a slow learning rate (Fig. 1j; $\alpha_{\mathrm{rew}}=0.24$, mean across sessions).

Together, this demonstrates that mice will perform a probabilistic reversal learning task to obtain VTA DA stimulation, and that their decision-making can be well-explained by a reinforcement learning model that estimates the trial-by-trial values of each possible action.

### VTA DA stimulation is sufficient to generate action value representations in the striatum, particularly in intermediate CP

**Extended Data Figure 3.**
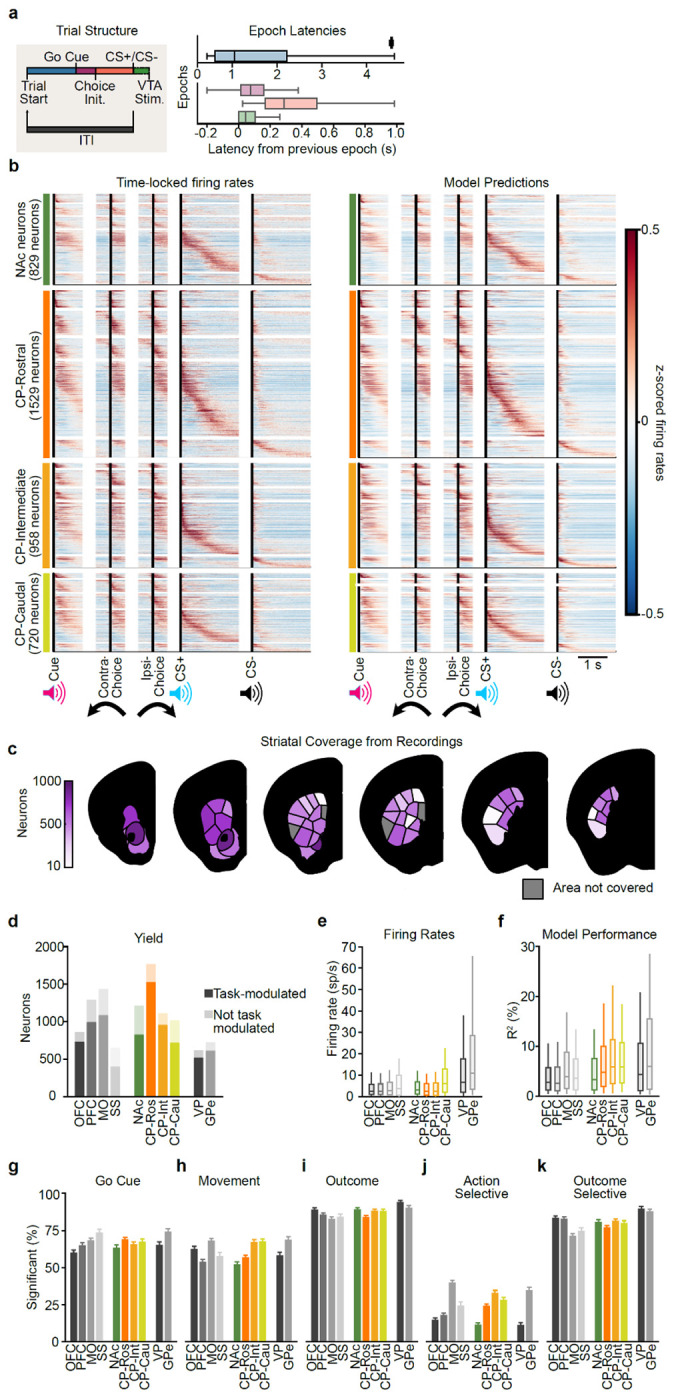
Recording statistics and modulation by task events. **a,** Box plot showing the distribution of latencies for between the main task events. Inter-trial-interval (ITI) from CS+/CS− onset to next trial start (Black), Trial Start to Go Cue (Blue), Go Cue to Choice Initiation / First Movement (Purple), Choice Initiation to CS+/CS− (Red), CS+ to Lick/First VTA stim pulse (Green). The boxplots show the median, inter-quartile range (IQR), and whiskers extending to the furthest point within 1.5x IQR (n = 22,365 trials from 41 sessions, 8 animals). **b,** *Left:* Time-locked, z-scored firing rate peri-event time histograms (PETHs) for all task-modulated single units from each striatal subregion. *Right:* Corresponding encoding model predictions for each neuron. In both plots, activity is aligned to task events: go cue, action (contra- or ipsilateral movement onset), and outcome (CS+ or CS− onset). Neurons within each subregion were first sorted based on their peak response timing in the concatenated PETHs using only odd trials, and then only even trials were used to generate the PETHs shown in the figure. The model predictions are shown in the same order as the neurons. **c,** Yield of task-modulated single units for striatal domains in the ^91,92^ atlas. Grey areas were not covered by the recordings. **d,** Yield of single-units recorded in cortical, striatal and pallidal subregions, categorized by whether each neuron was task-modulated (dark bars) or not (light extension to the barsbars). For all encoding model analyses, only task-modulated single units were included. **e,** Box plot showing firing rates across regions. **f,** Box plot showing the bilinear encoding model’s model performance, quantified as the cross-validated percentage of variance explained (R^2^) for neurons in each subregion. Box plots represent the 10th, 25th, 50th, 75th, and 90th percentiles. **g–k,** We classified neurons as significantly encoding task events by comparing the performance of a “full” encoding model with kernels for all events present to reduced models with a subset of event kernels removed (see Methods for details). We used this strategy to assess significance for (**g**) the go cue, by removing only the go cue kernel in the reduced model; (**h**) action, by removing both the left and right movement onset kernels; (**i**) outcome, by removing the CS+, laser stimulation, and CS− kernels; (**j**) choice-selectivity, by replacing the left and right movement onset kernels with a single movement onset kernel that was not choice-specific; and (**k**) outcome-selectivity, by replacing the CS+ and CS− kernels with a single CS kernel that was not reward-specific and also removing the laser stimulation onset kernel. All values were computed relative to a null distribution (see Methods for details). Error bars in bar plots represent the mean ± s.e.m.

**Figure 2.**
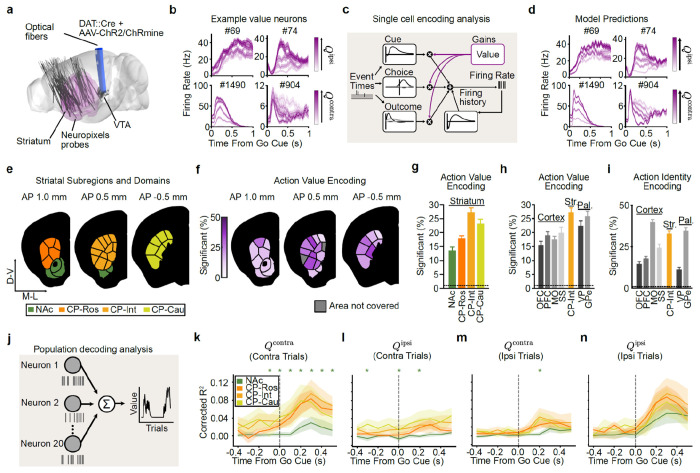
VTA DA stimulation is sufficient to generate action value representations, most prominently in the intermediate CP. **a,** 3D rendering of the Allen CCF showing optogenetic fiber (blue) placement over the VTA (black) and reconstructions of all Neuropixels probe trajectories (grey) in the striatum (purple) and associated brain structures. Recordings were bilateral but reconstructions are projected onto the left hemisphere. **b,** Example peri-stimulus time histograms (PSTHs) for four CP neurons (#69:CP-Int, #98:CP-Int, #1490:CP-Cau, #904:CP-Cau), showing their firing rate after the go cue. Trials are divided based on quintiles of $Q^{\mathrm{ipsi}}$ (top) or $Q^{\mathrm{contra}}$ (bottom) action values. Lines and shading represent the mean ± s.e.m. across trials. Only ipsilateral trials are plotted for $Q^{\mathrm{ipsi}}$ and only contralateral trials for $Q^{\mathrm{contra}}$. **c,** Schematic of the single-neuron encoding analysis (see Methods for details). **d,** PSTH predictions from the encoding model for the same example neurons as in (**b**). **e,** Coronal sections illustrating the locations of analyzed striatal subregions and domains within three anterior-posterior (AP) subregions. Each striatal subregion (rostral, intermediate, and caudal CP and the NAc) has a unique color, and the striatal domains within those subregions are separated by black lines. **f,** Coronal sections identical to (**e**) with color saturation indicating the percentage of neurons significantly encoding action value in each striatal domain. Grey areas were not covered by the recordings. **g,** Percentage of neurons significantly encoding action values (either $Q^{\mathrm{contra}}$ or $Q^{\mathrm{ipsi}}$) in recorded striatal subregions. **h,** Percentage of neurons significantly encoding action values ($Q^{\mathrm{contra}}$ or $Q^{\mathrm{ipsi}}$ in recorded cortical and pallidal subregions along with CP-Int from **(g)**. *n* = 829 NAc, 1529 CP-Ros, 958 CP-Int, 720 CP-Cau, 733 OFC, 996 PFC, 1088 MO, 402 SS, 522 VP, and 615 GPe task-modulated single units, see Extended Data Table 3 for mapping between these names and Allen CCF regions. **i,** Same as (**h**) but for action identity encoding (choice). **j,** Schematic of the population decoding analysis (see Methods for details). **k–n**, Time-resolved population decoding performance (corrected R^2^) in 100 ms bins for $Q^{\mathrm{contra}}$ (**k**,**m**) or $Q^{\mathrm{ipsi}}$ (**l**,**n**) action values in trials with contralateral (**k**,**l**) or ipsilateral trials (**m**,**n**) choices. Decoding was based on groups of 20 randomly chosen neurons. Lines and shading represent the mean ± s.e.m. across sessions (*n* = 18 NAc, 20 CP-Ros, 15 CP-Int, and 13 CP-Cau sessions). The corrected R^2^ is computed by subtracting the mean R^2^ of a session-specific null distribution from the mean R^2^ of decoders applied to the observed behavioral data (see Methods for details). * indicate time points where decoding performance for CP was significantly different from NAc (*P* ≤ 0.05, from two-tailed Mann–Whitney U test at each timepoint). In all bar plots, bars denote the mean ± bernoulli s.e.m. across all recorded neurons and the dashed lines indicate the chance level for significance (1%; see Methods for details on significance testing).

We next set out to test the long-standing hypothesis that VTA DA neuron activity generates action value representations in the striatum^1,4,20,31,38,39,86,87^. To accomplish this, we surveyed neural activity across the striatum using high-density Neuropixels probes^88^ as mice performed our task (Fig. 2a and Extended Data Fig. 3a–e; 5,109 single units from 37 sessions from 8 mice).

The activity of most striatal units was significantly modulated by task events (79.0%). Of these task-modulated neurons, 67.3% were modulated by the go cue, 61.0% by the actions, and 87.0% by the outcomes (*P* ≤ 0.01 based on a linear encoding model; see Methods for details; Extended Data Fig. 3f–k).

For a subset of these neurons, the responses to these task events were further modulated by trial-by-trial action values (as estimated from the Q-learning model). For example, the event-related firing of the example neurons shown in Fig. 2b increased or decreased monotonically as a function of contralateral ($Q^{\mathrm{contra}}$) or ipsilateral ($Q^{\mathrm{ipsi}}$) action value (contralateral and ipsilateral are defined relative to the recording hemisphere, see Methods).

This implies that VTA DA neuron stimulation is indeed sufficient to create action value representations in at least a subset of striatal neurons. Our high-density Neuropixels recordings allowed us to map where in the striatum such DA-driven action value representations were located.

First, to quantify if and how each neuron’s event-related activity was modulated by the trial-by-trial $Q^{\mathrm{contra}}$ and $Q^{\mathrm{ipsi}}$ action values, we used a bilinear encoding model^44,82^ (Fig. 2c,d; see Methods for details). Briefly, each neuron’s spiking activity was modeled using a sum of temporal kernels aligned to the task events (go cue, contralateral/ipsilateral action (relative to the recording hemisphere), CS+/−, and VTA DA stimulation) that were each modulated multiplicatively by trial-by-trial action values inferred by the behavioral model. We then quantified the strength and significance of action value coding by calculating how much the inclusion of the action values in the model improved our ability to explain each neuron’s spiking activity relative to null distributions that preserved the temporal correlations in the data^89,90^ (see Methods for details). Across the entire striatum, our encoding model identified 20.2% of neurons as significantly encoding trial-by-trial action values.

We then examined the strength of action value encoding across the striatum based on the divisions from the mouse cortico-striatal projectome^91,92^, which organizes the striatum into four higher-order divisions (the rostral, intermediate, and caudal CP and the NAc; referred to here as “subregions”) that are then further parcellated into thirty-one lower-order subdivisions (referred to here as “domains”; Fig. 2e).

**Extended Data Figure 4.**
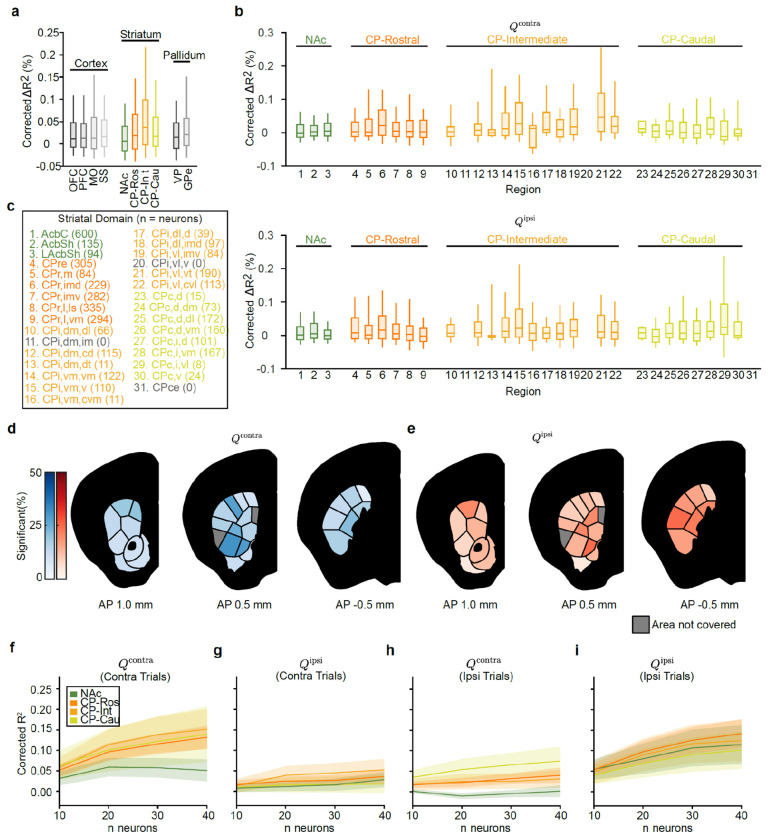
Supplementary information related to encoding and decoding analyses of $Q^{\mathrm{contra}}$ and $Q^{\mathrm{ipsi}}$. **a,** Box-plot showing the increase in null corrected variance explained ($\Delta R^{2}$) for adding the action value gains ($Q^{\mathrm{contra}}$ or $Q^{\mathrm{ipsi}}$) to the encoding model across cortical, striatal and pallidal regions. Corrected $\Delta R^{2}$ values are computed by subtracting the mean $\Delta R^{2}$ of a session-specific null distribution from the $\Delta R^{2}$ of the encoding model applied to the observed behavioral data (see Methods for details). b,c, Distribution of corrected $\Delta R^{2}$ values for $(b)Q^{\mathrm{contra}}$ or $(c)Q^{\mathrm{ipsi}}$ action value encoding in each striatal domain, grouped by subregion. Box plots represent the 10th, 25th, 50th, 75th, and 90th percentiles. Legend displays relation between numbers in x-axis and striatal domain names. **d,e** Coronal sections with color saturation indicating the percentage of neurons significantly encoding (**d**) $Q^{\mathrm{contra}}$ or $(e)Q^{\mathrm{ipsi}}$ QIpsi action values in each striatal domain (n=829 NAc, 1529 DS-Ros, 958 DS-Int, 720 DS-Cau). Grey areas were not covered by our recordings. f−i, Population decoding performance (corrected R^2^) 200 ms after the go cue, as a function of neuron count and region for (f,g) $Q^{\mathrm{contra}}$ or (h,i) $Q^{ipsi}$ action values in trials with contralateral (**f,h**) or ipsilateral (**g,i**) choices. Lines and shading represent the mean ± s.e.m. across sessions with at least 40 neurons in that region (*n* = 10 NAc, 17 CP-Ros, 13 CP-Int, and 9 CP-Cau sessions).

Within the four subregions, we found that modulation by action value was most common in the intermediate CP (27.3% of neurons) and least common in the NAc (13.6% of neurons; Fig. 2f,g; similar conclusions from ΔR^2^ in Extended Data Fig. 4a). Intermediate CP had significantly more neurons encoding action value than NAc (*P* < 1e-11 by χ^2^ test) and rostral CP (*P* < 1e-7 by χ^2^ test) with a trending effect relative to caudal CP (*P* = 0.054 by χ^2^ test; *P* < 1e-6 when comparing ΔR^2^ by Mann–Whitney U test). In contrast, the NAc had fewer neurons encoding action value than any other subregion (*P* < 0.01 vs. CP-Ros, *P* < 1e-11 vs. CP-Int, and *P* < 1e-5 vs. CP-Cau by χ^2^ tests). Furthermore, within the intermediate CP, individual domains had as many as 35.9% of neurons significantly encoding action value (Fig. 2f; see also Extended Data Fig. 4b–e).

The stronger value coding in intermediate CP than NAc was the case despite firing rates, and therefore the statistical power of our encoding model^93,94^, being significantly higher in the NAc when compared to the intermediate CP (*P* < 0.001 by Mann–Whitney U test; Extended Data Fig. 3e).

### Stronger encoding of action identity in MO but action value in intermediate CP

We compared these encoding model results in striatum to those in cortex, because the cortex provides a major monosynaptic input to the striatum (Fig. 2h). We performed cortical recordings in the somatosensory cortex (SS), motor cortex (MO), prefrontal cortex (PFC), and orbitofrontal cortex (OFC; see Extended Data Table 3 for Allen region groupings). In the cortex, neurons encoding action values were less common compared to the striatal subregion where action value was most strongly encoded (intermediate CP; *P* < 1e-8 vs. OFC, *P* < 1e-4 vs. PFC, *P* < 1e-6 vs. MO, and *P* < 0.01 vs. SS by χ^2^ tests). In contrast, the action identity (contralateral versus ipsilateral choice) was most strongly encoded in the MO compared not only to other cortical regions (*P* < 1e-7 vs. OFC, PFC, and MO by χ^2^ tests) but also to all striatal subregions, including the intermediate CP (*P* < 0.01 by χ^2^ test) (Fig. 2i). Thus, action encoding dominates in MO while action value encoding dominates in intermediate CP.

We also recorded from output structures of the CP and NAc: the globus pallidus externus (GPe) and ventral pallidum (VP), respectively (Fig. 2h). In the GPe, we observed a high fraction of neurons encoding action value, at levels that were comparable to the intermediate CP (*P* = 0.51 by χ^2^ test). In contrast, the fraction of neurons encoding action value was significantly lower in the VP when compared to the intermediate CP (*P* < 0.05 by χ^2^ test). These results suggest that value representations in the striatum are projected downstream to the pallidum.

### Population analyses corroborate stronger representations of action value in CP relative to NAc

The analyses above relied on single neuron encoding models. Population-level decoding corroborated the conclusion that action value representations generated by VTA DA stimulation are preferentially localized to the CP relative to the NAc. We trained linear decoders to predict the trial-by-trial estimates of $Q^{\mathrm{contra}}$ or $Q^{\mathrm{ipsi}}$ using the activity of groups of 20 randomly chosen, simultaneously recorded units from each striatal subregion around the time of the go cue (Fig. 2j–n and Extended Data Fig. 4f–i). In order to control for neural activity related to the different actions, we trained separate action value decoders for contralateral versus ipsilateral choices.

On trials in which the mouse turned the wheel in the contralateral direction, the value of both chosen (contralateral) and unchosen (ipsilateral) actions was better decodable from CP subregions than from the NAc in multiple 100 ms bins before and after the go cue (Fig. 2k,l; *P* < 0.05 by Mann–Whitney U tests). For both contralateral and ipsilateral choices, the value of non-chosen actions was less strongly decoded than the chosen action (compare Fig. 2k to Fig. 2l, and Fig. 2m to Fig. 2n; statistics in Extended Data Table 7), although decoding of the non-chosen value was still above chance across CP subregions (Fig. 2l). Decoding in the CP tended to be stronger than NAc on ipsilateral choice trials as well, although the difference was not significant in this case (Fig. 2m,n).

**Extended Data Figure 5:**
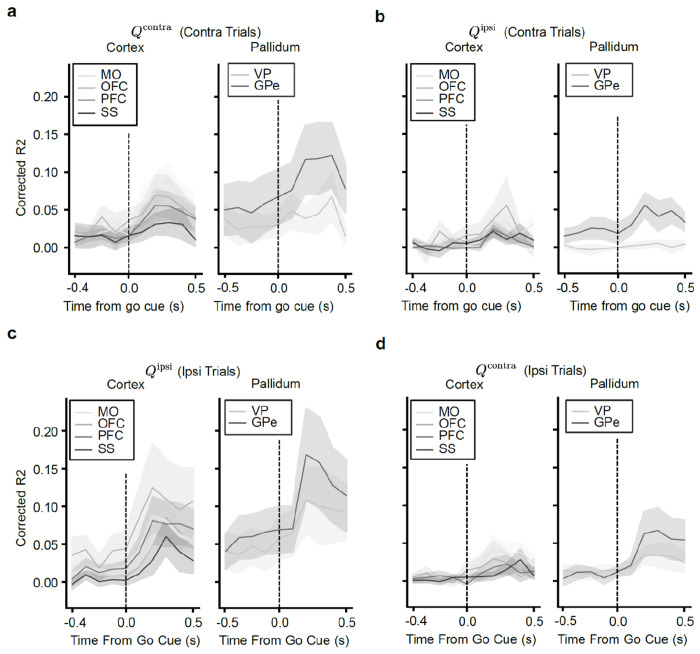
Action value decoding from striatal inputs (cortex) and outputs (pallidum). Time-resolved population decoding (groups of 20 randomly picked groups combos of 20 neurons) performance (corrected R^2^) for (**a**, **d**) $Q^{\mathrm{contra}}$ or (**b**, **c**) $Q^{\mathrm{ipsi}}$ action values in trials with (**a**, **b**) contralateral or (**c**, **d**) ipsilateral trials choices. The corrected R^2^ is computed by subtracting the mean R^2^ of a session-specific null distribution from the mean R^2^ of decoders applied to the observed behavioral data (see Methods for details). Lines and shading represent the mean ± s.e.m. across sessions (*n* = 19 MO, 10 OFC, 11 PFC, 12 SS, 11 GPe, and 13 VP sessions).

Downstream of the striatum, action value decoders trained using population activity from the GPe, a major output of the CP, performed much better than decoders trained using activity from the VP, an output of the NAc (Extended Data Fig. 5a–d). Indeed, action value decoding in the VP was hardly better than chance, despite the presence of single neurons that significantly encoded this variable (Fig. 2h). Thus, action value decoding is stronger in the CP relative to the NAc, as well as in the respective downstream targets of these structures.

### VTA DA stimulation generates relative and state value representations, most prominently in intermediate CP

**Extended Data Figure 6.**
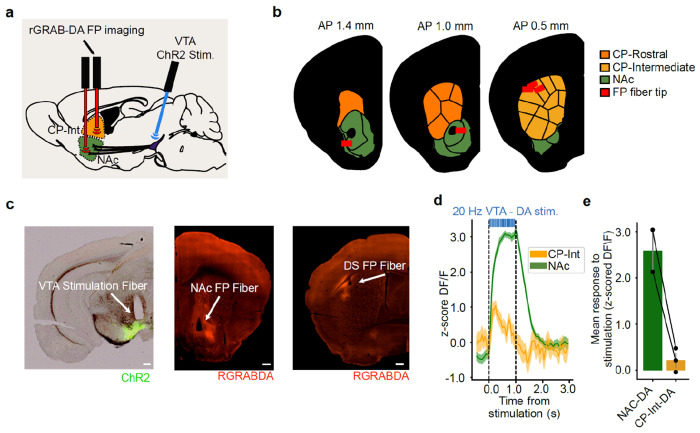
VTA stimulation primarily drives DA release in the NAc. **a,** Schematic of the fiber photometry experiment for the validation of the VTA DA stimulation approach. **b,** Histological reconstruction of photometry fiber tip locations (red rectangles) projected to the nearest 400-μm slice on the Allen CCF with striatal domains segmented ^91,92^. **c,** Representative images confirming optical fiber placement and virus expression. *Left:* Optogenetics fiber track for VTA DA stimulation and ChR2 expression (green) in the VTA. *Center:* Photometry fiber track and rGRAB-DA expression in the NAc. *Right:* Photometry fiber track and rGRAB-DA expression in the intermediate CP. Scale bars, 200 μm. **d,** Example peri-stimulus time histogram, from one animal, of DA transients in the NAc (green) and intermediate CP (orange) evoked by VTA DA stimulation (447 nm; 20 Hz; 5 ms pulse width; 8 mW). The signal represents the z-scored change in the ΔF/F fluorescence trace. Lines and shading represent the mean ± s.e.m. across 20 trials. The dashed line represents the VTA DA stimulation period. **e,** Quantification of the mean stimulation-evoked DA response in the NAc and intermediate CP during the 1 s VTA DA stimulation period (epoch represented with vertical dashed lines in (**d**)). Points indicate individual animals (n = 2 NAc animals, 3 intermediate CP animals), and lines connect data from the same animal. Error bars represent the mean ± s.e.m. across animals.

We were surprised that action value representations generated by VTA DA stimulation were localized to the CP (relative to the NAc; Fig. 2), given that the NAc is the major target of DA release by VTA DA neurons^2,3^, a fact which we confirmed in our own stimulation experiments (Extended Data Fig. 6a–e).

However, so far we have only considered modulation of neural activity by the value of each action. A prominent hypothesis (the actor-critic model^39,50,87^) instead suggests that the NAc might compute a different form of value: state value (the trial-by-trial reward expectation, *V*), which could be used for calculating reward prediction errors and also serve as a motivational signal. The CP, in turn, might encode relative value (the difference in value between the two actions, Δ*Q*), which could be used to select a choice.

**Figure 3.**
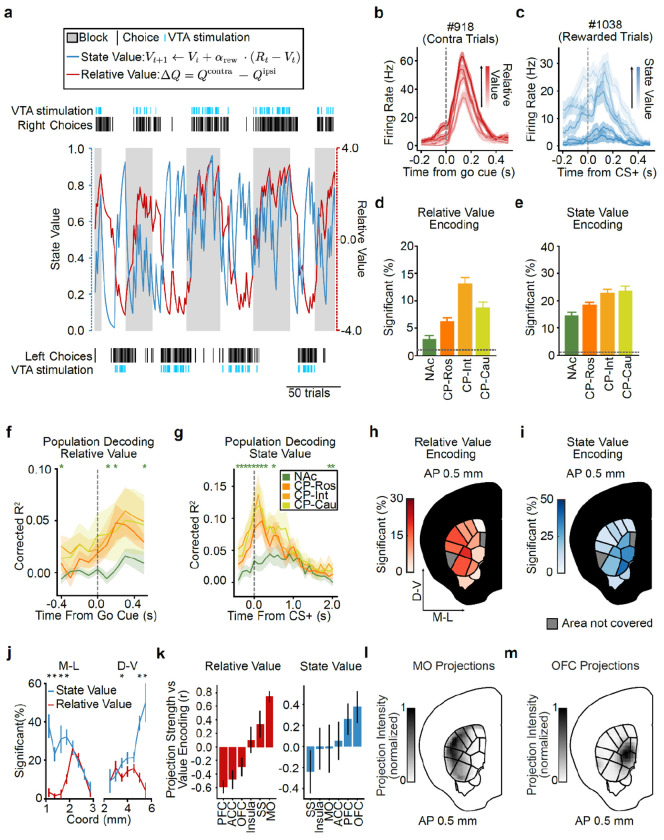
VTA DA stimulation generates relative and state value representations in the intermediate CP, with different spatial organizations. **a,** Estimated state (blue) and relative (red) value for a representative behavioral session. Black vertical ticks at the top and bottom represent individual right and left choices. Shaded grey indicates blocks where the right choice had the higher probability of reward. Blue vertical ticks indicate VTA stimulation. **b,** Example PSTH for a striatal neuron (#918:CP-Ros) in contralateral trials for different relative values. **c**, Example PSTH for a different striatal neuron (#1038: CP-Int) for rewarded trials with different state values. In (**b,c**), trials are divided based on value quintiles of relative and state value. Lines and shading represent mean ± s.e.m. **d,** Percentage of neurons in each striatal subregion significantly encoding relative value. Bar plots represent mean ± s.e.m. (*n* = 829 NAc, 1529 DS-Ros, 958 DS-Int, 720 DS-Cau). The dashed line indicates the chance level for significance (1%). **e,** Same as (**d**) but for state value. **f,** Time-resolved population decoding performance (corrected R^2^) for relative value in trials with contralateral choices (based on groups of 20 randomly chosen neurons). The corrected R^2^ is computed by subtracting the mean R^2^ of a session-specific null distribution from the mean R^2^ of decoders applied to the observed behavioral data (see Methods for details). Lines and shading represent the mean ± s.e.m. across sessions (*n* = 18 NAc, 20 CP-Ros, 15 CP-Int, and 13 CP-Cau sessions). * indicates time points where decoding performance for CP was significantly different from NAc (*P* ≤ 0.05, from two-tailed Mann–Whitney U test at each timepoint). **g,** Same as (**f**) but for state value around the time of the CS+, on rewarded trials only. **h,** Coronal sections illustrating analyzed striatal domains within CP-Int. Saturation indicates the local percentage of neurons significantly encoding relative value. Grey areas were not covered by the recordings. **i**, Same as **(h)** but for state value encoding. **j,** Average percentage of neurons encoding state (blue) and relative (red) value along the mediolateral (left, ML) and dorsoventral (right, DV) axes within intermediate CP. Lines and error bars represent mean ± binomial s.e.m. (*P < 0.05, χ^2^ test) **k,** Correlation coefficients (Pearson r) between the normalized cortical projection intensity to each striatal domain from the Allen Connectivity Atlas ^95^ and that domain’s encoding of relative (red) and state (blue) value. Bar plots indicate mean ± bootstrap standard deviation (s.d.). From left to right: cortical projection intensities were obtained from 9 pre-frontal cortex (PFC), 34 anterior cingulate cortex (ACC), 16 orbito-frontal cortex (OFC), 16 Insula, 70 somatosensory cortex (SS), and 60 motor cortex (MO) anterograde injections (see Extended data table 6 for details). **l,m,** Mean normalized intensity of projections from primary and secondary motor cortex (**l**, MO) and orbito-frontal cortex (**m**, OFC) to intermediate striatum.

Therefore, we next investigated whether state and relative values were differentially encoded across striatal subregions and domains, and whether state value would be preferentially localized to the NAc. To this end, we again used a bilinear encoding model to test how strongly each neuron’s task-related activity was modulated by trial-by-trial estimates of value, but this time considered modulation by relative value ($\Delta Q=Q^{\mathrm{contra}}-Q^{\mathrm{ipsi}}$) or state value (V; Fig. 3a), rather than $Q^{\mathrm{contra}}$ or $Q^{\mathrm{ipsi}}$ (Fig. 2c–h). We identified many neurons whose activity was correlated with one or both of these variables (example neurons: Fig. 3b,c).

**Extended Data Figure 7:**
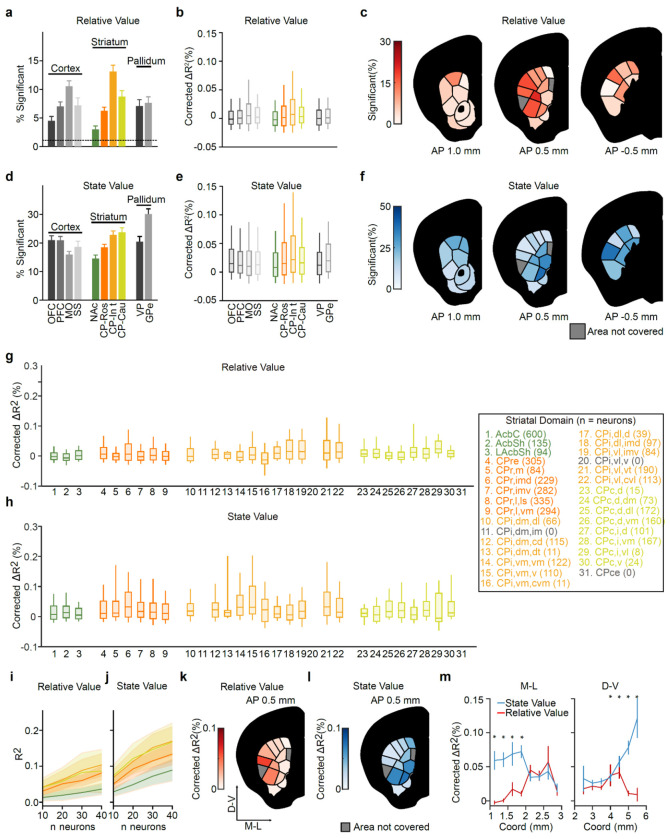
Supplementary information on state and relative value representations in striatum. **a,** Percentage of neurons significantly encoding relative value, **b,** and mean null corrected explained variance increase (ΔR^2^) for adding the relative value gain to the encoding model across cortical, striatal and pallidal regions. Bar plots show mean ± s.e.m. Box plots represent the 10th, 25th, 50th, 75th, and 90th percentiles (*n* = 829 NAc, 1529 DS-Ros, 958 DS-Int, 720 DS-Cau). Corrected ΔR^2^ are computed by subtracting the mean ΔR^2^ of a session-specific null distribution from the ΔR^2^ of the encoding-model applied to the observed behavioral data (see Methods for details). **c,** Coronal sections with color saturation indicating the percentage of neurons significantly encoding relative value in each striatal domain. Grey areas were not sufficiently covered by the recordings. **d,e,f,** Same as (**a-c**) but for state value. **g,h,** Distribution of ΔR^2^ values for (**g**) relative or (**h**) state value encoding in each striatal domain, grouped by striatal subregion. Box plots represent the 10th, 25th, 50th, 75th, and 90th percentiles. Legend displays relation between numbers in x-axis and striatal domain names. **i, j,** Population (20 neurons) decoding performance (R^2^) for (**i**) relative and (**j**) state value as a function of neuron count and striatal subregion. Line and shading represent mean ± s.e.m. across sessions with at least 40 neurons in that region (*n* = 10 NAc, 17 CP-Ros, 13 CP-Int, and 9 CP-Cau sessions). **k, l,** Coronal sections illustrating analyzed striatal domains within CP-Int, the subregion where we found the strongest value encoding. Saturation indicates the local corrected ΔR^2^ from adding (**k**) relative or (**l**) state value gains. Grey areas were not sufficiently covered by the recordings. **m,** Average corrected ΔR^2^ from adding relative value (red) and state value (blue) gains to the encoding model along the mediolateral (ML; left), and dorsoventral (DV; right) axes within intermediate CP. Line and error bar represent mean ± binomial s.e.m (*P < 0.05, Mann–Whitney U test).

Similar to $Q^{\mathrm{contra}}$ and $Q^{\mathrm{ipsi}}$ (Fig. 2), relative (Fig. 3d and Extended Data Fig. 7a–c,g) and state (Fig. 3e and Extended Data Fig. 7d–f,h) values were preferentially encoded in the CP relative to the NAc. Intermediate CP had significantly more neurons encoding relative value than all other striatal subregions (P<1e−13vs. NAc, P<1e−8vs. CP-Ros, and P<0.01vs. CP-Cau by $x^{2}$ tests), whereas NAc had fewer neurons encoding relative value than any other subregion (P<0.001 vs. CP-Ros, P<1e−13 vs. CP-Int, and P<1e−5vs. CP-Cau by $x^{2}$ tests; Fig. 3d). A similar proportion of neurons significantly encoded state value in intermediate and caudal CP (*P* = 0.67 by χ^2^ test; Fig. 3e), however the magnitude of this state value coding, based on the explained variance ($\Delta R^{2}$) of neural activity across the entire population, was significantly stronger in intermediate CP (<0.01 by Mann-Whitney U test; Extended Data Fig. 7e). Once again, NAc had fewer neurons encoding state value than any other striatal subregion (<0.05 vs. CP-Ros and P<1e−5 vs. CP-Int and CP-Cau by $X^{2}$ tests; Fig. 3e).

We next trained population-level decoders for both relative and state value (Fig. 3f,g and Extended Data Fig. 7i,j). Relative value decoders were trained using only contralateral trials to control for representations of the action itself, while state value decoders were trained using only rewarded trials to control for the response to the outcome itself. These population-level analyses supported the preferential localization of both relative (Fig. 3f and Extended Data Fig. 7i) and state (Fig. 3g and Extended Data Fig. 7j) values to the CP in comparison to the NAc. Moreover, state value decoding in the CP was apparent earlier in the trial than in the NAc (from the first timepoint tested, 500–400 ms before CS+ onset; Fig. 3g). Thus, not only action values ($Q^{\mathrm{contra}}$ and $Q^{\mathrm{ipsi}}$ ), but also relative and state values (ΔQ and V ), are most prominently represented in the CP relative to the NAc.

Within the intermediate CP, relative and state value representations had distinct spatial organizations. Specifically, relative values were more prominently encoded in dorsolateral domains of the intermediate CP, whereas state values were more strongly represented in ventromedial domains (Fig. 3h–j and Extended Data Fig. 7k–m). To investigate how these spatial patterns relate to the topography of cortical inputs to the striatum, we turned to the Allen Projection Atlas^95^ (Fig. 3k–m). Interestingly, the encoding of relative value — a variable which could be used for action selection — was positively correlated with the projection pattern from the MO (*r* = 0.715 Pearson correlation; Fig. 3k,l). The MO is of course important for generating actions^96–106^ and, in our data, was the cortical region that best encoded the animals’ choice (Fig. 2i). In contrast, the encoding of state value was positively correlated with the OFC projection pattern (*r* = 0.317 Pearson correlation; Fig. 3k,m), a cortical region implicated in value^40,41,107^ and state^108–110^ representations.

### Value coding is strongest in intermediate CP across learning and forgetting rates

**Figure 4.**
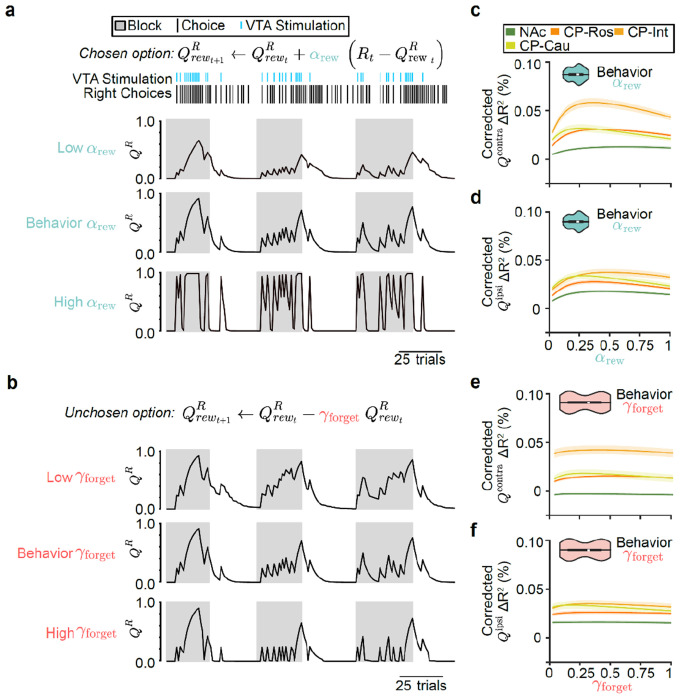
Across learning and forgetting rates, value coding is strongest in intermediate CP **a,** Right action values ($Q^{R}$) of an example session for the behaviorally fit learning rate ($\alpha_{\mathrm{rew}}$, middle panel), a high learning rate (0.95; bottom panel), and a low learning rate (0.1; top panel). Black vertical ticks represent individual right choices. Blue vertical ticks indicate VTA stimulation. Shaded grey areas indicate blocks where the right choice had the higher probability of reward. **b,** Same as (**a**) but for forgetting rates ($\gamma_{\mathrm{forget}}$). **c,** Average change in corrected explained variance (corrected $\Delta R^{2}$) for the single-neuron encoding model as a function of the learning rate parameter ($\alpha_{\mathrm{rew}}$) for $Q^{\mathrm{contra}}$. Colored lines indicate different striatal subregions. Violin plots illustrate the distribution of $\alpha_{\mathrm{rew}}$ obtained from fitting the behavior (plot limits described in Fig. 1j). Corrected $\Delta R^{2}$ computed by subtracting the mean $\Delta R^{2}$ of pseudosession-based null distribution from the $\Delta R^{2}$ of the encoding model applied to the observed behavioral data (see Methods for details). **d,** Same as (**c**) but for $Q^{ipsi}$, action values. **e,f,** Same as (**c,d**) but for the forgetting parameter ($\gamma_{\mathrm{forget}}$) instead of $\alpha_{\mathrm{rew}}$. Lines and shading represent mean ± s.e.m. across sessions.

We next explored the hypothesis that a mismatch in the timescale of value computation between the NAc and behavior could potentially explain why value representations appear relatively weak in the NAc (Figs. 2,3). Action values depend on parameters that determine the timescale over which previous rewards are integrated (learning rate: $\alpha_{\mathrm{rew}}$; Fig. 4a) and forgotten (forgetting rate: $\gamma_{\mathrm{forget}}$; Fig. 4b). We so far have considered neural correlates of action values (Figs. 2,3) based on the values of these parameters that best explain the animals’ behavior (Fig. 1). However, previous fiber photometry recordings suggest that DA release in the NAc and CP may reflect lower and higher learning rates, respectively^64^. Inspired by this result and other findings about temporal scales across the dopamine system^69,111,112^, we wondered whether individual neurons in the NAc may in fact integrate (or forget) rewards more slowly than the animals’ behavioral policy — and than neurons in the CP.

**Extended Data Figure 8.**
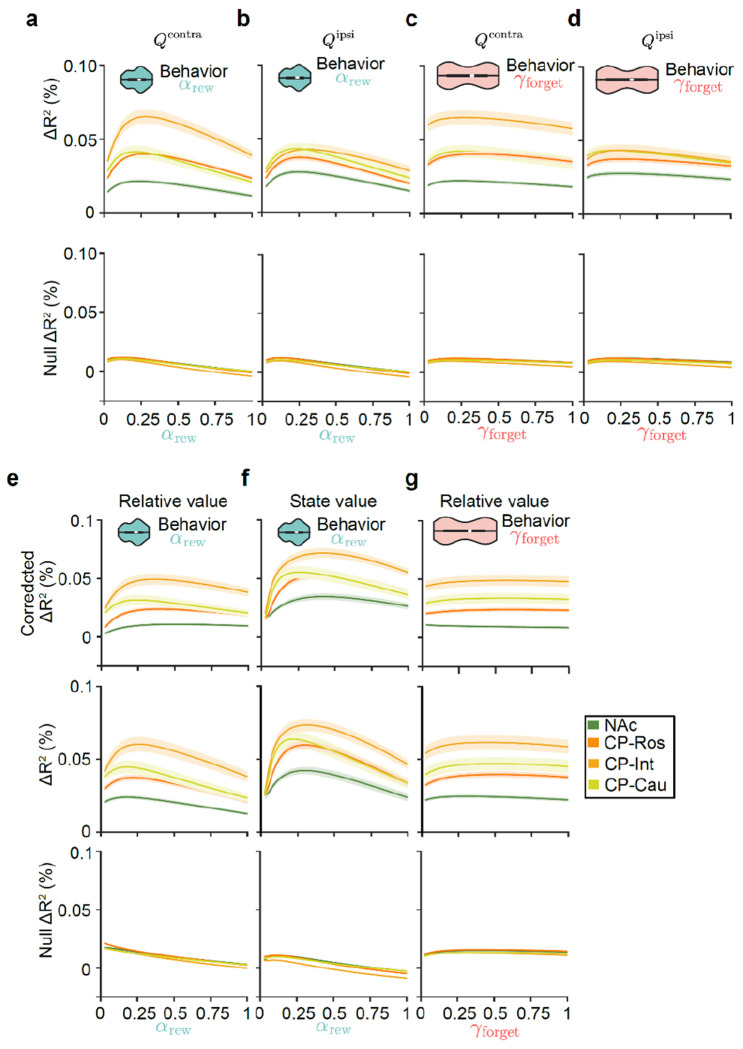
Across learning and forgetting rates, relative and state value coding is strongest in intermediate CP. **a,b,** Improvement in uncorrected explained variance ($\Delta R^{2}$) in the encoding model as a function of the learning rate parameter $\left(\alpha_{\mathrm{rew}}\right)$ used to generate the (**a**) $Q^{\mathrm{contra}}$, and (**b**) $Q^{ipsi}$ values inputted into the model for the real session (top) or null distribution of pseudosessions (bottom). Colored lines indicate different striatal subregions. **c,d,** Same as (**a,b**) but when varying the forgetting parameter ($\gamma_{\mathrm{forget}}$) used to generate the (**c**) $Q^{\mathrm{contra}}$ and (**d**) $Q^{\mathrm{ipsi}}$ values instead of $\alpha_{\mathrm{rew}}$. **e-g,** Top: Improvement in corrected explained variance ($\Delta R^{2}$) as a function of the learning rate parameter (**e,f**, αRew) or forgetting parameter (**g,** $\gamma_{\mathrm{forget}}$) used to generate the (**e,g**) relative, and (**f**) state values inputted into the model. No $\gamma_{\mathrm{forget}}$ equivalent of (**f**) is plotted since the state value update rule does not contain a forgetting term. Middle: Same as top row but for uncorrected $\Delta R^{2}$. Bottom: Same as middle row but for the pseudosession-based null distribution. Corrected $\Delta R^{2}$ (top row) is computed by subtracting the mean $\Delta R^{2}$ of the pseudosession-based null distribution (bottom row) from the $\Delta R^{2}$ of the encoding model applied to the observed behavioral data (middle row; see Methods for details). For all plots lines and shading represent mean ± s.e.m. across sessions. Violin plots illustrate the distribution of $\alpha_{\mathrm{rew}}$ or $\gamma_{\mathrm{forget}}$ obtained from fitting the behavior. Plot limits are the limits of the observed data, the boxplot within the violin marks the median (white) and IQR (thick black line).

To test this idea, we asked what learning rate best explains individual neurons’ activity across the striatum. To do this, we refit encoding models to the neural activity using trial-by-trial action values for different learning rates ($\alpha_{\mathrm{rew}}$ varying between 0 and 1). This revealed that the CP — especially the intermediate CP — more strongly encoded both contralateral and ipsilateral action values across all learning rates (Fig. 4c,d and Extended Data Fig. 8a,b).

Within the CP, in particular for contralateral action value (Fig. 4c), the learning rate that best explained trial-by-trial variations in neural activity was similar to the learning rate that best described the animals’ choice behavior (Q-learning model $\alpha_{\mathrm{rew}}=0.24$, mean across sessions). We repeated the same analysis for relative and state value, instead of action values, and observed the same phenomenon (Extended Data Fig. 8e–g).

We also performed an equivalent analysis in which we varied the forgetting rate ($\gamma_{\mathrm{forget}}$), rather than the learning rate (Fig. 4b). Again, we found that the CP more strongly encoded both contralateral and ipsilateral action values across all potential forgetting rates (Fig. 4e,f and Extended Data Fig. 8c,d). However, we observed that varying the Q-learning model’s forgetting rate had very little impact on the neural encoding of action values^113^, implying that learning rate is better reflected in neural activity than forgetting rate.

Together, these analyses suggest that the relatively weak DA-driven action value correlates in the NAc is not because neurons in this part of the striatum are representing value using a slower learning rate than the animal’s behavioral policy. Instead, across learning and forgetting rates, value coding is strongest in the intermediate CP, with neurally estimated learning rates roughly matching the behaviorally estimated learning rates.

### VTA DA stimulation endows the CS+ with value, which the mice then work for in our task

So far, we have shown that across different types of value and different learning and forgetting rates, VTA DA stimulation produces value representations that are strongest in the intermediate CP. The fact that these representations are strongest in this subregion — which is not the major direct target of the stimulated VTA DA neurons^2,3^ (Extended Data Fig. 6a–e) — suggests an indirect path between VTA DA stimulation and the formation of action value representations.

What could mediate an indirect path between VTA DA stimulation and action values? We hypothesized that rather than directly producing action values, this stimulation could contribute to learning the value of a stimulus^30,31,114,115^ — the CS+ in our task — and this valuable stimulus could in turn serve as a conditioned reinforcer to produce valuable actions. This hypothesis was inspired by previous work on conditioned reinforcement, in which repeatedly pairing a stimulus with DA stimulation in the NAc (but not the CP) leads to the stimulus itself being able to reinforce actions^30^, as well as the observation that a sensory stimulus can potentiate self-stimulation of VTA DA neurons^116^.

This hypothesis makes two core predictions, which we test below: 1) That even though action value representations are relatively sparse in the NAc, responses to the CS+ will be readily apparent; and 2) That the mice are working for the CS+ itself in our task, and not directly for the VTA DA stimulation.

**Extended Data Figure 9.**
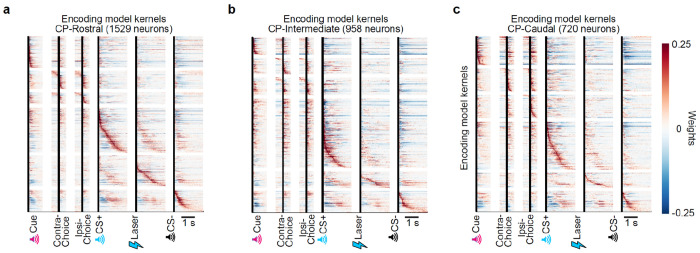
Outcome coding ending is prevalent across striatal subregions. **a-c,** Time-locked normalized kernels from the encoding model for individual neurons in (**a**) CP-Rostral, (**b**) CP-Intermediate, (**c**) CP-Caudal. Neurons are sorted by the peak across their kernels.

**Figure 5.**
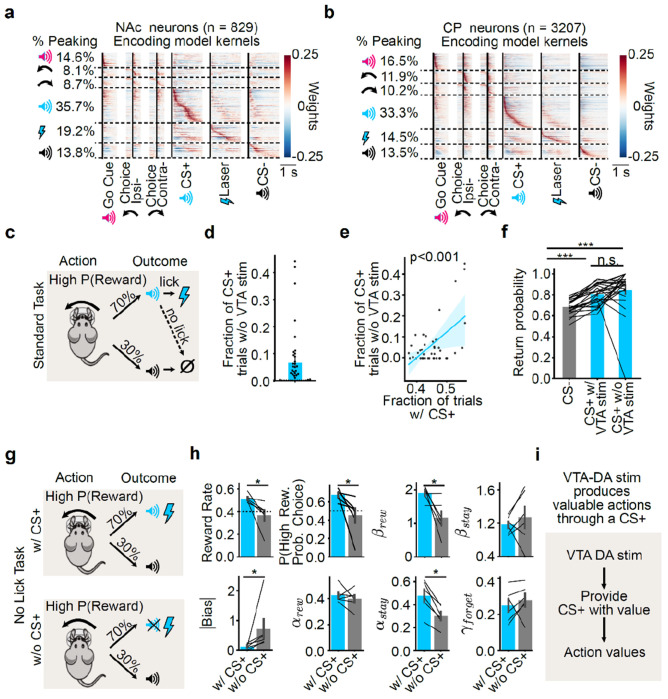
VTA DA stimulation endows the CS+ with value, which the mice then work for in our task. **a,** Encoding model kernels for individual neurons in the NAc, with neurons sorted by the peak across their kernels. “% peaking” on the left represents the fraction of neurons with the peak kernel response for each task event. **b,** Same as (**a**) but for CP. **c,** Schematic of the outcome contingency in the probabilistic reversal learning task, highlighting the licking requirement for the acquisition of the VTA stimulation. **d,** Fraction of CS+ trials without VTA DA stimulation. Individual points represent sessions. *n* = 41 sessions from 8 mice. **e,** Correlation across sessions between the fraction of CS+ trials without VTA stimulations (CS+ trials without VTA stimulations/total CS+ trials) and overall performance (CS+ trials/total trials). Line represents linear regression (see Extended Data Table 7 for statistical details). *n* = 41 sessions from 8 mice. **f,** Average probability of repeating the previous choice (left or right) as a function of trial outcome. Individual lines represent sessions where there were at least 5 CS+ trials without VTA DA stimulation (**P < 0.001, paired Wilcoxon signed-ranked test with Bonferroni-Holm’s correction). *n* = 24 sessions from 8 mice. **g,** “No Lick” task for examining the role of the CS+ on VTA DA seeking behavior, in which the licking requirement is removed and the CS+ and VTA DA are presented simultaneously (w/ CS+), or else the CS+ is omitted (w/o CS+). **h,** On sessions with and without the CS+ presentation, bar plots of the average reward rate, fraction of high reward probability choices (P(high reward probability choice)), and behavioral model parameters $\left(\beta_{\mathrm{rew}},\beta_{\mathrm{stay}},Bias,\alpha_{\mathrm{rew}},\alpha_{\mathrm{stay}},\gamma_{\mathrm{forget}}\right)$. Lines represent individual mice (*P < 0.05, see Extended Data Table 7 for statistical details). n = 6 animals. **i,** Overall, our data points towards a model where VTA DA stim produces action values indirectly, by first providing a CS+ with value (i.e., making a “conditioned reinforcer”), which is then used to generate action value representations in the CP. All bar plots show mean ± s.e.m. n.s. signifies p>0.05.

Indeed, even though the NAc had relatively few neurons significantly encoding action values (13.6%, Fig. 2f,g), encoding of the CS+ was very common (55.9%; see Methods for details). Based on the kernels of our encoding model, which distinguish neural responses between task events (including the CS+ from VTA DA stimulation), the plurality of NAc neurons (35.7%) were most strongly activated by the CS+ relative to other task events (Fig. 5a). This predominant response to the CS+ was widespread across striatum: the plurality of neurons in all subregions were most strongly activated by the CS+ (Fig. 5b and Extended Data Fig. 9a–c).

Next, we tested the idea that mice in our task are working for the CS+ itself, rather than for VTA DA stimulation. Upon hearing the CS+ on rewarded trials, VTA DA stimulation must be “collected” by licking a dry spout (Fig. 5c,d). We hypothesized that if animals were working for the CS+, and not for VTA DA stimulation itself, then they might at times forgo collecting the VTA DA stimulation and that, when doing so, their subsequent choice behavior would be unaffected. We found support for this hypothesis. In the sessions with the highest performance, mice tended to have a lower VTA DA stimulation collection rate (*P* < 0.001; Fig. 5e). This suggests that high-performing mice may indeed be working to receive the VTA DA stimulation-paired CS+, rather than the stimulation itself.

**Extended Data Figure 10.**
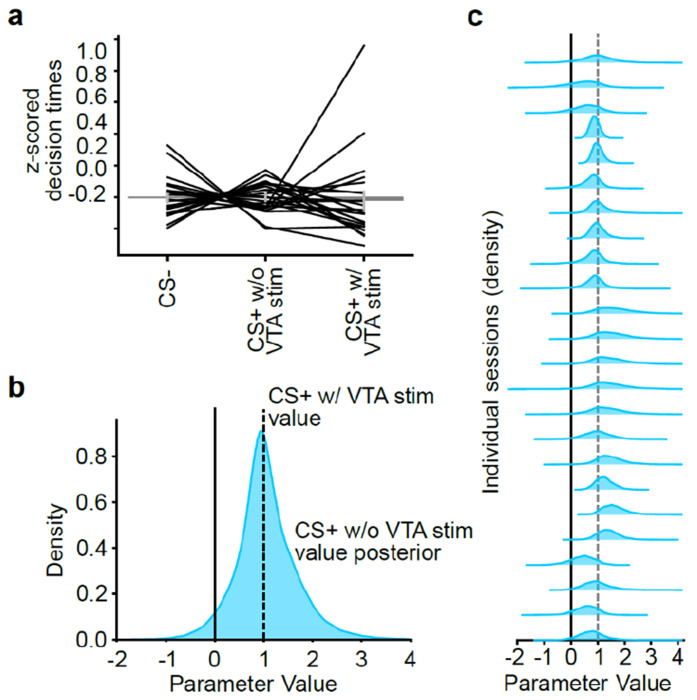
CS+ fitted value is significantly greater than 0, and similar to CS+ w/ VTA stimulation value. **a,** Average normalized decision times (Time from Go Cue to decision) after trials with different outcomes. Individual lines represent sessions. Bar plots show mean ± s.e.m. **b,** Posterior distribution of the CS+ w/o laser parameter (comparison to 1 which is the pre-set value for the CS+ with laser that is then scaled by $\beta_{\mathrm{rew}}$, See Equations 24 and 25 in Methods for details) across sessions. **c,** Same as (**b**) but for individual sessions. For all panels we included all sessions with at least 5 trials in each outcome condition (*n* = 24 sessions from 8 mice), so that we were able to estimate the value of the CS+ w/o VTA stim.

Moreover, following CS+ trials with versus without VTA DA stimulation, the animals’ behavior was similar, which implies that VTA DA stimulation did not impact their next trial behavior. This was true for the probability of repeating a choice (Fig. 5f) as well as the next trial latency (a readout of motivation; Extended Data Fig. 10a).

The fact that behavior was similar after a CS+ trial regardless of whether VTA DA stimulation was received (Fig. 5f) suggests that action values may be updated in a similar manner across these outcomes. To test this, we re-fit our Q-learning model, this time leaving the value of the CS+ without VTA DA stimulation as a free parameter. If the CS+ is rewarding (without VTA DA stimulation), we would expect the fitted value of this parameter to be positive. Furthermore, if its value is similar to the value of the CS+ with VTA DA stimulation (which is preset to 1), then we would expect this parameter to be close to 1 as well. We found that this was indeed the case. The fitted value of the CS+ without VTA DA stimulation was positive and extremely close to 1 both on average (Extended Data Fig. 10b) and on individual sessions (Extended Data Fig. 10c).

Together, these analyses indicate that mice update their choice behavior and action values equally after CS+ trials regardless of whether VTA DA stimulation was actually collected. This suggests that the animals were working for the CS+ and not for VTA DA stimulation itself.

To test this idea more directly, we next examined the effect on performance of experimentally removing the CS+. We trained a separate cohort of mice on a variant of our task in which VTA DA stimulation and the CS+ co-occured immediately after the choice (i.e., we removed the licking requirement to obtain VTA DA stimulation so it began simultaneously with the CS+). Then, we compared performance on sessions with and without the CS+ (“No Lick Task with CS+” versus “No Lick Task without CS+”; Fig. 5g). Removing the CS+ led to a dramatic reduction in task performance, which was evident both from the animals’ reward rates and from the estimated weight of reward-seeking (*β_rew_*) in our behavioral model (*P* < 0.05 by Mann–Whitney U test; Fig. 5h). Note that the CS+ and the VTA DA stimulation occurred simultaneously in this task variant, and therefore the importance of the CS+ to performance cannot be attributed to a shorter or stronger temporal relationship between the action and the CS+ (compared to the VTA DA stimulation).

Thus, our behavioral data (Fig. 5c–h) are consistent with a model in which mice are working for the VTA DA stimulation-associated CS+ rather than for VTA DA stimulation itself. Together with our neural data (Figs. 2–4 and Fig. 5a,b), this suggests that VTA DA activity may support action learning indirectly. First, DA release (potentially in the NAc) endows stimuli with value as animals discover which stimuli are temporally associated with VTA DA neuron activation. Then, these valuable stimuli in turn produce action value representations in downstream sites, such as the intermediate CP (Fig. 5i).

## DISCUSSION

Here, we provide the first experimental support for the long-standing theory that midbrain DA neurons can produce action value representations.

We also provide evidence for an indirect path between the firing of VTA DA neurons and the generation of such action values. First, even though the CP is not a major projection target of VTA DA neurons, action value correlates (including state and relative value) are strongest there. In addition, mice do not directly work for the VTA DA stimulation, and instead rely on a conditioned stimulus to reinforce their actions. Finally, this VTA DA-paired conditioned stimulus is encoded widely throughout the brain (including in the NAc, which is a major target of the VTA DA system), more so than any other task event.

Together, this suggests a conditioned reinforcer-based model of DA-mediated learning, where, rather than directly producing action value representations, VTA DA neuron activity produces valuable stimuli^30,114,117,118^. These stimulus value representations in turn produce action value representations downstream of the VTA DA target sites, in particular where we observed the strongest value correlates within the CP. This downstream influence could potentially be mediated by the ascending spiral architecture of the basal ganglia^119,120^.

This framework differs from the traditional actor-critic model of the basal ganglia^115^ because state value, which in this task fluctuates on a trial-by-trial basis with the CS+ delivery, is preferentially located in CP, rather than the NAc (Fig. 3), and because this state value does not play a role in modulating the action values. Instead, the CS+ can be seen as criticizing the actions. The CS+ value, which is (relatively) constant in this task, might be computed in NAc^30^.

This framework could help resolve many questions posed by our data and by the field. It explains why mice will not perform the task without the conditioned stimulus (Fig. 5), as they are actually working for that stimulus. It also explains why action value correlates are weak in the NAc (Figs. 2–4), while conditioned stimulus responses are strong there (Fig. 5a). This framework also appears to fit with our behavioral modeling (Fig. 1), in which the version of Q-learning with the CS+ as a reward slightly outperformed the other models, including the version where the VTA DA stimulation served as an RPE directly. Moreover, this framework is consistent with a recent paper^30^ that demonstrated DA release in the NAc (but not in the CP) creates valuable stimuli for which an animal will work. Finally, it aligns with a classic view of the NAc as fundamental for Pavlovian associations^121–123^, while also clarifying how it can indirectly impact operant behavior or action selection, such as in the present task, by potentially endowing stimuli with a form of what has been called incentive salience^124,125^.

These conclusions depended on using VTA DA stimulation instead of a natural reward in our task. This is because a natural reward would have sensory properties (e.g., taste, sound, smell, sight) — in other words, a natural reward would have its own conditioned stimuli. Moreover, a natural reward would activate DA neurons not only in VTA but also in the substantia nigra (SNc), which project directly to the CP and can likely reinforce actions directly^36^, without the intermediary of a conditioned stimulus.

Here, we focused on the longer-run, learning-based effects of VTA DA neuron activity. There are also shorter-term influences of VTA DA neurons over action, motivation, and forms of model-based valuation^85,126–129^, as well as affective heterogeneity in the VTA DA system^130^, which are beyond the scope of the present work.

In sum, our work clarifies the power of VTA DA stimulation to mediate learning based on trial-and-error, along with limits to that power. Compelling prospects for future studies include dissecting the circuitry mediating the influence of VTA DA stimulation-induced conditioned reinforcers on action selection, as well as parallel experiments focusing on SNc (rather than VTA) DA stimulation.

## METHODS

### ANIMALS:

11 male and 6 female adult heterozygous DAT::cre (Strain #006660, Jackson Laboratory) mice crossed with wild-type C57BL/6J (Strain #000664, Jackson Laboratory) were used in all experiments. Mice were maintained on a reversed 12 h light cycle and all experiments were conducted during the dark phase. Mice were between 3 and 8 months of age during surgery, training, and testing. Ambient temperature was maintained at 21-26 °C and humidity at 30-70% at all times. All experimental procedures were approved by the Princeton University Institutional Animal Care and Use Committee following the NIH Guide for the Care and Use of Laboratory Animals.

### STEREOTAXIC SURGERY:

All surgeries were performed under isoflurane anaesthesia (5% for inductions, 1-2% for maintenance). All mice receive preoperative antibiotics (5 mg/kg, Baytril). Analgesia (10 mg/kg, Ketofen) was provided preoperatively, and postoperatively for 2 days.

Mice underwent the following surgical schedules, depending on the experiment:

- Neuropixels recordings (Figs. 1–5; Extended Data Fig. 1–5,7–10; 6 male and 2 female):
  1st surgery (Pre Training, 3 months old mice): Initial surgery where animals were headplated, viruses were injected, optical fibers and ground pins implanted, and the recording well prepared.
  2nd-4th Surgery (Post Training, 4-6 months old mice): Individual craniotomies for acute Neuropixels recordings. This surgery was repeated 1-2 more times to access extra recording locations.
- Fiber photometry (Extended Data Fig. 6; 3 female):
  1st surgery (Pre Training, 2 months old mice): Initial surgery where viruses were injected.
  2nd Surgery (3-4 months old mice): Surgery where animals were implanted with a headplate and optical fibers implanted.
- No Lick task (Fig. 5; 5 male and 1 female):
  1st surgery (Pre Training, 3 months old mice): Surgery where animals were headplated, viruses were injected, optical fibers and ground pins implanted.

#### Headplate implant:

All mice had a headplate implanted as described in the IBL standardized protocol^131,132^. Briefly, the scalp and underlying periosteum was removed. Bregma and lambda were leveled, and a small steel headbar was centered at −6.9 mm anterior-posterior (A-P) relative to bregma and cemented to the skull with Metabond (Parkell).

#### Virus injections:

For animals used in Neuropixels recordings with optogenetic stimulation of the VTA, AAV2/5-EF1a-DIO-ChRmine-mScarlet-WPRE-hGHpA (titer: 9e12 genome copies/ml, Princeton Neuroscience Institute viral core) or AAV2/5-EF1a-DIO-hChR2-WPRE-hGHpA (titer: 2.65e12 genome copies/ml, Princeton Neuroscience Institute viral core) was injected bilaterally in the VTA (± 0.75 mm M-L, −3.1 mm A-P, −4.75 mm D-V). 500 nl were infused in each hemisphere at a speed of 100 nl/min. For the fiber photometry recordings, 500 nl of AAV2/5-EF1a-DIO-hChR2-WPRE-hGHpA (titer: 2.65e12 genome copies/ml, Princeton Neuroscience Institute viral core) was injected bilaterally in the VTA (± 0.75 mm M-L, −3.1 mm A-P, −4.75 mm D-V) and 250 nl of AAVdj-hSyn-GRAB-rDA3m-WPRE-hGHpA (titer: 5e12 genome copies/ml, Princeton Neuroscience Institute viral core) was injected in the NAc into each of 2 injection sites (1st: ± 0.75 mm M-L, −3.1 mm A-P, −4.75 mm D-V, 2nd: − 0.75 mm M-L, −3.1 mm A-P, −4.25 mm D-V) and CP (1st: − 2.5 mm M-L, 0 mm A-P, −3 mm D-V, 2nd: −2.5 mm M-L, 0 mm A-P, −2.5 mm D-V). All viruses were infused at a speed of 100 nl/min (Nanofil syringes with a UMP3 ultramicro pump, WPI). At the end of the injection, we waited a minimum of 5 minutes before retracting the syringe.

#### Optical fiber implants:

Optical implants used for optogenetic stimulation were made in house. The fiber (∅300 mm core, 0.39 NA, 2.5 mm ferrule, Thorlabs) was polished and tested for an even transmission profile and a minimum transmission efficiency of 80%. For optogenetic stimulation of the VTA, optic fibers were implanted bilaterally at the following coordinates: ± 0.4 mm medial-lateral (M-L), −3.1 mm anterior-posterior (A-P), −4.15 mm dorsal-ventral (D-V). These locations were reached with a 10° M-L/D-V rotation (rotated coordinates: −3.1 mm A-P, ± 1.1 mm M-L, 4 mm D-V).

For fiber photometry recordings, low-autofluorescence optical fibers encased in a ferrule (∅200 mm core, 0.37 NA, 1.25 mm ferrule, Neurophotometrics) were implanted unilaterally at each of the following locations (fiber tip location relative to bregma): DLS (−2.6 mm M-L, 0 mm A-P, −2.8 mm D-V) and NAc (−1 mm M-L, 1.45 mm A-P, −4.5 mm D-V).

#### Recording well preparation:

For animals used for Neuropixels recordings, at the time of headplate implantation, a well was built with cement around the perimeter of the dorsal skull and covered with UV transparent glue (Norland Optical Adhesives #81, Norland) and a small ground pin (82K7797, Newark electronics) was inserted on the right hemisphere. This UV glue protected the exposed skull and was removed on the first craniotomy surgery for recordings.

### BEHAVIOR:

#### Behavioral apparatus:

We utilized the International Brain Laboratory’s standard behavioral setup, with comprehensive information on its parts and operation available in their documentation ^132,133^. Detailed component lists and setup instructions for the training and electrophysiology rigs can be found in ^134^ and ^135^, respectively.

The behavioral setups included “training” rigs that were not set up for electrophysiology, as well as an “electrophysiology” rig that was set up for electrophysiology. The training rigs were constructed with Thorlabs pieces inside a small soundproof cabinet (9U acoustic wall cabinet 600 3 600, Orion). The rigs featured an LCD screen (LP097Q, LG), a custom 3D-printed mouse holder to headfix the mouse in front of the screen, and a steering wheel (86652 and 32019, LEGO) positioned beneath its forepaws. A spout, connected to a water reservoir and controlled by a solenoid valve ( 225P011-21, NResearch), delivered water without obstructing the mouse’s view. The rigs also included a speaker (HPD-40N16PET00-32,Peerless by Tymphany) above the screen for generating sounds. A rotary encoder (05.2400.1122.1024, Kluber) tracked wheel movements. Visual stimulus timing was measured using a photodiode (Bpod Frame2TTL, Sanworks), in order to detect screen pixel flips. All task-related devices were controlled by a Sanworks Bpod State Machine, with task logic programmed in Python and visual stimulus/video handled by Bonsai and BonVision ^136^. The electrophysiology rig was a modified version of the training rig, situated on an air table (VH3048W-OPT Newport) within a larger custom soundproof cabinet to accommodate electrophysiology equipment. It differed by using a USB camera (CM3-U3-13Y3M-CS, Point Grey) for lick detection, a custom speaker (Champalimaud Foundation, V1.1), and 4x micromanipulators sitting in platforms above the mouse holder (uMp-4, Sensapex) outside the mouse and camera’s field of view. In the electrophysiology rig, behavior, video, audio and neural (electrophysiology or phometry) signals were synchronized through TTLs sent to a PXI system (National Instruments) ^133^.

All electrophysiological and photometry recordings, as well as pharmacological inactivation experiments, were conducted in the electrophysiology rig. Both rig types were used for training in the probabilistic reversal learning task.

#### Probabilistic reversal learning task:

Mice were trained to perform a probabilistic reversal learning task for VTA stimulation, which required them to track their history of rewards and choices to select the choice with higher reward probability (Fig. 1–4,5a–f). To initiate each trial, animals were required to hold the wheel still for a quiescent period that varied between 0.2 to 0.5 s. After this quiescent period, two identical Gabor patches (100% contrast, 0.1 cycles/degrees, vertical orientation, random phase) were presented on the left and right sides of a screen. Choices were made by rotating the wheel. Wheel movement was coupled in closed-loop fashion to the horizontal position of the Gabor patches with a gain of 4 visual degrees per mm of wheel displacement (2.17 visual degrees per degree of wheel rotation). A choice was registered when the mouse centered one of the two Gabor patches on the screen (35°). Importantly, the patches gave no information about reward probabilities. Clockwise and counterclockwise movements were classified as left and right choices respectively. When a choice was registered, the animal was either rewarded or unrewarded and the selected Gabor (left or right) froze in the center for 4 s regardless of the choice’s outcome. If a choice earned a potential VTA stimulation, a 100 ms duration, ~ 65 dB narrowband sound (12-13 kHz) indicated VTA stimulation availability, and served as a positive conditioned stimulus (CS+).Upon the onset of this tone, VTA stimulation was contingent on the animal’s licking of a dry spout, with licking detected using a video camera ^137^. If the mouse licked the spout at any point during the 1 s period following this sound, stimulation was delivered. The VTA stimulation consisted of a 1 s train of laser stimulation (ChR2: 447 nm , ~8 mW, 5 ms pulse width, 20 Hz; ChRmine: 532 nm , ~0.5 mW, 5 ms pulse width, 20 Hz) generated by a DPSS laser (Shanghai Laser and Optics & Co) controlled with a Pulse Pal (Sanworks). Rewards were not baited. Unrewarded trials were signaled by 0.5 s of ~ 65 dB white noise, which served as a negative conditioned stimulus (CS −). A 4-5 s inter-trial interval (ITI), following the CS+/CS− onset, followed each trial regardless of its outcome. If no choice was completed within 60 s from the go cue, the CS− was played and a new trial started after the ITI. Whether a choice was rewarded was dependent on a probabilistic block structure. Within each block, one choice (left or right) was designated as the high-reward probability choice (70% reward probability), while the other choice was designated as the low-reward probability choice (10% reward probability). Block lengths had a default random length between 20 and 40 trials, drawn from a uniform distribution. At each block transition, the high- and low-reward probability sides were switched. To prevent side biases, a debiasing mechanism was implemented such that blocks did not advance until the mouse made at least 7 choices toward the high-reward probability side within the preceding 10. This debiasing procedure could therefore extend block length beyond its default 40 trial upper limit.

All recordings were performed with mice that were already fully trained to perform the task. Sessions were only included for analysis when mice completed at least 9 blocks and showed win-staying behavior, that is when animals were more likely to repeat a choice when rewarded (after a CS+). This quality control measure was not applied in experiments not using the standard probabilistic learning task (No Lick Task, Fig. 5g–h). Sessions lasted between 60-90 minutes (546.5 ± 16.3 trials per session, mean ± s.e.m.).

#### Training in the probabilistic learning task:

To train on the task, mice underwent a shaping procedure consisting of 4 stages. During shaping, the animals received 4ul of 10% sucrose solution as a reward, instead of optogenetic stimulation. While the same CS− as in the final task signaled unrewarded trials, the sucrose reward itself was accompanied by the sound of the solenoid valve but importantly was not paired with the 12-13 kHz narrowband CS+ used in the final task. In Stage 1, the high- and low-probability choices had a 100% and 0% probability of reward, respectively. Block lengths in this stage were 10 to 30 trials. Unrewarded choices had a 10s ITI. To facilitate the association of turning the wheel with the selection of the left or right Gabor patch, in this stage, the two patches had different spatial frequencies (0.1 cycles/degrees for the left Gabor and 0.5 cycles/degrees for the right Gabor). Once mice reached 70% correct choices in Stage 1 (14.3 ± 3 sessions on average), they advanced to Stage 2 . Stage 2 was identical to Stage 1, but the left and right Gabor patches had the same spatial frequency of 0.1 cycles/degrees (as in the final task). Upon reaching 70% correct choices in Stage 2 (2.83 ± 0.542 sessions on average), mice progressed to Stage 3, where the high- and low-reward probability choices had a reward probability of 70% and 10%, respectively, as in the final task. Equally, ITI for both rewarded and unrewarded trials were 4-5 s, as in the final task. Once mice could complete at least 9 blocks and showed win-staying behavior (17.9 ± 4.6 sessions on average), they moved to Stage 4 (as long as a minimum of 4 weeks from viral injection had passed to ensure ChR2/ChRmine expression). Stage 4 consisted of one to two 30-min conditioning sessions to learn to lick for the VTA DA stimulation reward. During these sessions, mice received a 1 s train of VTA DA optogenetic stimulation (1 s train, 5 ms pulse width, 20 Hz) in response to licking a dry spout within a 1s window following the sound of the CS+ from the final task (an unheard sound up to this point in training). After this conditioning stage, mice were moved to the final task (probabilistic learning task).

#### Water restriction:

Water access was restricted from 1 week prior to the start of training until the end of the experiment. Initial water restriction was necessary as mice worked for water rewards during early shaping. Later, when reward identity changed to optogenetic DA stimulation, mice were kept on water restriction to maintain a comparable internal state between testing and training. Weights never dropped more than 20% from their pre-water restriction weight, and mice consumed a daily minimum of 1 ml of water per 25 g of body weight. During the training phases of the probabilistic learning task (where mice received water rewards) most mice were able to obtain the majority of their daily allocation of water through the task alone. When this minimum was not achieved during the task, mice were supplemented at the end of the day. Once the mice moved to optogenetic rewards, they were provided with water at the end of every day.

#### No Lick Task:

In order to understand how the presence of a CS+ affected choice behavior, we performed an experiment (Fig. 5g–h) where mice worked for VTA DA optogenetic stimulation paired with an auditory CS+ (“No Lick Task with CS+”) or without it (“No Lick Task without CS+”). In our standard probabilistic reversal learning task, the CS+ indicated the presence of a potential optogenetic reward that could be obtained by licking an empty spout during the 1s following the CS+. This introduced a potential confound: Without removing this lick requirement any change in task performance in the no CS+ task could be due to a reduction in the collection of optogenetic stimulation rewards and not the absence of the CS+, since the animal might not know when to lick after making a rewarding choice. To prevent this, in this experiment optogenetic stimulation delivery was automatically provided after making a rewarding choice, removing the lick requirement. Additionally, the co-occurance of the CS+ and the VTA DA stimulation in this task variant ensured that the importance of the CS+ to performance cannot be attributed to a shorter or stronger temporal relationship between the action and the CS+ (compared to the action and VTA DA stimulation). Animals used in this task were first trained in the “No Lick Task with CS+” following the shaping steps described for the standard probabilistic reversal learning task. Once trained, mice first experienced the“ No Lick Task with CS+” followed by the “No Lick Task without CS+” in the following session.

### BEHAVIORAL MODELS:

To understand the decision-making processes of mice in our task, we built and tested three families of models: 1) Q-learning, 2) policy gradient (REINFORCE), and its extension 3) the actor-critic (Fig. 1.; Extended Data Fig. 2.; Extended Data Table 1). All models were designed to predict trial-by-trial choices based on slightly different assumptions about how mice learn from reward and experience. All models were designed to predict trial-by-trial choices based on different assumptions about how mice learn from reward and experience. Parameters were fit in a hierarchical Bayesian manner, allowing variability to be modelled at three distinct levels: the population, individual subjects, and experimental sessions.

We start by detailing the model families; we then describe the fitting procedure, describe the comparison and quality controls we used and finally how we derived point estimates of the decision variables that we then used for neural analyses.

#### Models:

##### Models 1-2: Extended Q-learning models:

We tested two versions of Q-learning models ^138,139^ (Extended Data Table 1), one where the CS+ was treated as a reward regardless of whether the VTA DA stimulation reward was collected (Model 1), and a version where VTA DA stimulation was treated as an RPE (Model 2). Both models were extended to incorporate mechanisms to account for forgetting (an exponential decay of unchosen option values) and perseveration (the tendency to repeat previous choices irrespective of outcome) which are known factors to affect decisions ^140^.

###### Model 1: Extended Q-learning model with the CS+ as reward

Q values for each choice (*a_t_* ∈ {0 if the choice was left, 1 if the choice was right}) were initialized at 0 at the start of each session. In every trial, t, the Q value for the chosen option ($a_{t}$) was updated according to the following equations:

(Equation 1) $$\text{Rewarded trials:}\;Q_{rew_{t+1}}^{a_{t}}\leftarrow Q_{rew_{t}}^{a_{t}}+\alpha_{rew}\left(1-Q_{rew_{t}}^{a_{t}}\right)$$

(Equation 2) $$\text{Unrewarded trials:}\;Q_{rew_{t+1}}^{a_{t}}\leftarrow Q_{rew_{t}}^{a_{t}}+\alpha_{rew}\cdot \left(0-Q_{rew_{t}}^{a_{t}}\right)$$

Here, $\alpha_{\mathrm{rew}}$ was a free learning rate parameter. Trials where a CS+ was delivered, were treated as *rewarded* independently of whether the VTA stimulation was later consumed. *Unrewarded* trials only included the trials where a CS− was delivered.

To account for the possibility that the value of unchosen options might decay over time, we introduced a forgetting mechanism into the model ^38^. For the unchosen option, ${\tilde{a}}_{t}=1-a_{t}$, the Q-value was updated according to:

(Equation 3) $$\text{Unchosen option}:\;Q_{rew_{t+1}}^{{\tilde{a}}_{t}}\leftarrow Q_{rew_{t}}^{{\tilde{a}}_{t}}-\gamma_{forget}\cdot Q_{rew_{t}}^{{\tilde{a}}_{t}}$$

Where $\gamma_{\mathrm{forget}}$ is a second learning rate on the unchosen option’s value.

To capture the observed tendency of subjects to repeat choices regardless of their outcome, we incorporated a perseveration term ^140–143^. This was done by introducing $Q_{{\mathrm{stay}}_{t+1}}^{a_{t}}$, which represented a weighted average of the mouse’s past choices. This ‘stay’ Q-value was updated as follows:

(Equation 4) $$\text{Chosen Option}:\;Q_{stay_{t+1}}^{a_{t}}\leftarrow Q_{stay_{t}}^{a_{t}}+\alpha_{stay}\left(1-Q_{stay_{t}}^{a_{t}}\right)$$

(Equation 5) $$\text{Unchosen Option}:\;Q_{stay_{t+1}}^{{\tilde{a}}_{t}}\leftarrow Q_{stay_{t+1}}^{{\tilde{a}}_{t}}+\alpha_{stay}\left(0-Q_{rew_{t+1}}^{{\tilde{a}}_{t}}\right)$$

Here $\alpha_{\mathrm{stay}}$ is a free learning rate specific to $Q_{{\mathrm{stay}}_{t+1}}^{a_{t}}$.

Finally, the probability of choosing a given option (${\mathrm{choice}}_{t,}$) at trial t, was then determined by passing the reward-based Q-values ($Q_{rew_{t+1}}^{a_{t}}$) and the perseveration Q-values ($Q_{{\mathrm{stay}}_{t+1}}^{a_{t}}$) through a sigmoid function:

(Equation 6) $$P_{t}\left(a_{t}\right)=sigmoid\left(Bias+\beta_{rew}\cdot \text{Δ}Q_{rew_{t}}+\beta_{stay}\cdot \text{Δ}Q_{stay_{t}}\right)$$

Where $\Delta Q_{{\mathrm{rew}}_{t}}=Q_{{\mathrm{rew}}_{t}}^{1}-Q_{{\mathrm{rew}}_{t}}^{0};\Delta Q_{{\mathrm{stay}}_{t}}=Q_{{\mathrm{stay}}_{t}}^{1}-Q_{{\mathrm{stay}}_{t}}^{0}$ are the relative preferences for choosing R over L. Here $\beta_{\mathrm{rew}}$ and $\beta_{\mathrm{stay}}$ are free parameters acting as weights for the value and perseveration components of the decision, respectively. *Bias* is a free parameter accounting for the subject’s innate choice bias in favor of a right choice.

###### Model 2: Extended Q-learning model with VTA DA stimulation as an RPE

Given that DA is often thought to serve as a reward prediction error (RPE), not a reward, we also considered a version of Q-learning where a) the DA stimulation took the role of an RPE and b) trials where a CS+ was delivered but the VTA DA stimulation was not collected were treated as unrewarded ^85^. Therefore in this model we substituted the $Q_{rew_{t+1}}^{a_{t}}$ update rule for the chosen rewarded action described in Equation 1 with:

(Equation 7) $$Q_{rew_{t+1}}^{a_{t}}\leftarrow Q_{rew_{t}}^{a_{t}}+\alpha_{rew}\cdot (1)$$

The unrewarded and unchosen options continued to be updated by the rules in Equation 2 and 3 respectively. The perseveration component was modelled as in Model 1 (Equation 4–5) and choice probabilities were calculated as described in Equation 6.

##### Model 3: REINFORCE:

We tested a version of a policy gradient-based model, ‘REINFORCE’ ^144^, which directly learns a decision variable that influences the probability of choice. This model operates on the principle of directly updating a decision variable or action propensity based on the outcome of a trial, rather than computing Q-values for actions. This model had no perseveration component since it was non-beneficial in early testing.

For each trial t, the propensity for choosing R over L(π) was updated based on the trial’s outcome:

(Equation 8) $$\text{Rewarded trials}:\;\pi_{t+1}\leftarrow \left(1-\alpha_{win}\right)\cdot \pi_{t}+\left(2a_{t}-1\right)\cdot \beta_{win}\cdot \left(1-P_{t}\left(a_{t}\right)\right)$$

(Equation 9) $$\text{Unrewarded trials}:\;\pi_{t+1}\leftarrow \left(1-\alpha_{loss}\right)\cdot \pi_{t}+\left(2a_{t}-1\right)\cdot \beta_{loss}\cdot \left(1-P_{t}\left(a_{t}\right)\right)$$

Here, $2a_{t}-1$ represents the action taken in trial t (+1 for Right, −1 for Left). The parameters $\alpha_{\mathrm{win}}$ and $\alpha_{\mathrm{loss}}$ are free forgetting rates specific to rewarded and unrewarded trials, respectively. Similarly, $\beta_{\mathrm{win}}$ and $\beta_{\mathrm{loss}}$ are free parameters that modulate the influence of the action prediction error $\left(1-P_{t}\left(a_{t}\right)\right)$ on the update for rewarded (CS+ delivered) and unrewarded outcomes (CS− delivered).

The probability of choosing a specific option $P_{t}\left(a_{t}\right)$ was determined by passing π through a sigmoid function:

(Equation 10) $$P_{t}\left(a_{t}=1\right)=\text{sigmoid}\left(Bias+\pi_{t}\right)$$

Where *Bias* is a free parameter accounting for any intrinsic preference for a right choice. π effectively accumulates evidence for right over left choices, with its magnitude and sign directly influencing the choice probability.

##### Model 4: Actor-Critic:

We also implemented an Actor-Critic model extending from the REINFORCE model^84^. This architecture combines a ‘Critic’ component, which learns to predict the value of states, with an ‘Actor’ component, which uses these value predictions to update its policy (i.e., its decision-making strategy). This framework allows for learning through both reward prediction and direct policy adjustments based on observed outcomes.

The Critic component was responsible for learning the expected value of the current trial, denoted as *V_t_*. In each trial *t*, the Critic first computed a reward prediction error (*δ_t_*), which quantifies the difference between the actual reward received (modeled using the CS+ as a reward) and the value (*V_t_*) of the trial:

(Equation 11) $$\text{Rewarded trials}:\;\delta_{t}=1-V_{t}$$

(Equation 12) $$\text{Unrewarded trials}:\;\delta_{t}=0-V_{t}$$

The trial value $V_{t}$ was then updated based on this prediction error and a learning rate, $\alpha_{\mathrm{rew}}$:

(Equation 13) $$V_{t+1}\leftarrow V_{t}+\alpha_{rew}\cdot \delta_{t}$$

At the start of each session $V_{t}$ was set to 0.

The Actor component was responsible for learning the π that directly determined the probability of choosing an action, similar to the REINFORCE model. Critically, its updates were modulated by the $\delta_{t}$ from the Critic, which acted as an adaptive baseline.

The π was updated depending on whether the trial was rewarded or unrewarded.


(Equation 14) $$\text{Rewarded trials}:\;\pi_{t+1}\leftarrow \left(1-\alpha_{win}\right)\cdot \pi_{t}+\left(2a_{t}-1\right)\cdot \beta_{win}\cdot \left(1-P_{t}\left(a_{t}\right)\right)\cdot \delta_{t}$$

(Equation 15) $$\text{Unrewarded trials}:\;\pi_{t+1}\leftarrow \left(1-\alpha_{loss}\right)\cdot \pi_{t}+\left(2a_{t}-1\right)\cdot \beta_{loss}\cdot \left(1-P_{t}\left(a_{t}\right)\right)\cdot \delta_{t}$$

As before, $2a_{t}-1$ signifies the action taken (+1 for Right, −1 for Left). The parameters $\alpha_{\mathrm{win}}$ and $\alpha_{\mathrm{loss}}$ are learning rates for the π update specific to rewarded and unrewarded outcomes, respectively. Similarly, $\beta_{\mathrm{win}}$ and $\beta_{\mathrm{loss}}$ are free parameters modulating the influence of the prediction error $\left(1-P_{t}\left(a_{t}\right)\right)$ and the Critic’s $\delta_{t}$ on the Actor’s update. The critical distinction here is the multiplication by $\delta_{t}$, which scales the learning based on the magnitude and sign of the reward prediction error.

The probability of choosing a specific option ($P_{t}\left(a_{t}\right)$) was then calculated using a sigmoid function, incorporating π and a *Bias*term as previously described in in Equation 10:

#### Fitting procedure:

Each model was fitted using Hamiltonian Markov chain Monte Carlo (MCMC) sampling in Stan (2.36.0) ^145^. We employed the standard methodology of generating latent parameter values from Gaussian distributions, and then using non-linear transformations to map them into the appropriate spaces for each model (e.g., using log-odds transform for parameters confined to the interval [0, 1], Extended Data Table 2).

We fitted the model hierarchically which allowed us to model variability simultaneously at three distinct levels: the population, individual subjects, and experimental sessions. By sharing information across these levels, we aimed to obtain more robust and precise estimates of the underlying parameters. The general hierarchical structure of the modelled parameters was as follows:

##### Population Level:


(Equation 16) $$\mu_{k}∼N(M,\tau ),\mu_{k}\in \mathbb{R}$$

(Equation 17) $$\text{ln}\left(\sigma_{k}\right)∼N(M,\tau ),\sigma_{k}\in \mathbb{R}$$

Where μ and σ represent the means and standard deviations for the k hyperparameters governing the distributions at the individual level. M is the mean and τ the standard deviation for the prior (see Extended Data Table 2 for the full list of priors for each parameter).

##### Individual Level:


(Equation 18) $$\theta_{k,i}∼N\left(\mu_{k},\sigma_{k}\right),\theta_{k,i}\in \mathbb{R}$$

Here, θ represents the individual-level means for subject i (where i=1,…,n, and n is the number of subjects) for each parameter k. These individual-level parameters were drawn from distributions parameterized by the population-level hyperparameters μ and σ.

##### Session Level:


(Equation 19) $$p\left(s_{k,j}\right)\propto 1,\;s_{k,j}\geq 0$$

(Equation 20) $$\psi_{k,i,j}∼N\left(\theta_{k,i},s_{k,j}\right),\psi_{k,i,j}\in \mathbb{R}$$

At the session level, ψ represents the session-specific parameter k, for subject i during session j (where j=1,…,z) where z represents the number of sessions for subject i. These session-level parameters were drawn from distributions parameterized by the individual-level means $\theta_{k,i}$ and variance $s_{k,j}.s_{k,j}$ was sampled from an uniform positive prior (Equation 19).

##### Prior Distributions:

Finally, we employed weakly informative prior distributions for all parameters (see Extended Data Table 2 for details) to allow the data to largely drive the posterior inference. At the population and individual level we ensured positivity of all standard deviations by applying a exp(.) transformation during sampling (see Extended Data Table 2 for details).

##### Inference:

For each model, we ran four independent chains, each with 1850 iterations, including a warm-up period of 350 iterations and a parameter adapt_delta set to 0.99. Posterior inference was based on the samples obtained after the warm-up period. Samples from the posterior distribution were extracted using the Python package PyStan.

#### Quality controls:

##### Posterior analysis:

Convergence of the MCMC chains was assessed by ensuring that the R^ statistic for all parameters was below 1.01. We also assessed the shape of the posterior with histograms to inspect the shape, central tendency and variance of the parameter estimates^146^.

##### Recovery analysis:

We performed recovery analysis to ensure that the fitted parameters were identifiable ^147^, that is to test whether the behavior can be best explained by a single set of parameters. To do this we generated a set of simulated data with the fitted parameters as the ground-truth and then fitted the data to these models. By comparing whether the fitted parameters closely followed the ground truth parameters we determined whether the parameter fits were identifiable (Extended Data Fig. 2b,c).

##### Confusion analysis:

While the recovery analysis detects whether the parameters within a model are meaningful, the confusion analysis tested whether different models are distinguishable ^147^. To do this we generated simulated data from the best model of each family (Q-learning with the CS+ modeled as a reward, REINFORCE and the Actor-Critic). We then fit each model to each simulated datasets. Finally, we tested whether the ground-truth model was significantly better at capturing the behavior than the other models (Extended Data Fig. 2b,c).

#### Decision Variable Estimation for Neural Analysis:

Finally, to correlate with neural activity, we estimated decision variables from our best-performing model, as determined by model comparison (i.e., the Q-values from Model 1: Extended Q-learning model with the CS+ as reward, Fig. 1c,d and Extended Data Table 1). These estimates were derived by running the fitted model “off-policy.” This involved feeding the mice’s actual choices and rewards into the model to update its Q values. For each set of post-warm up parameter draws (1500) from the posterior, we calculated the value of different decision variables for each model. This process generated a posterior distribution for the decision variable itself. Our final point estimate for the decision variable – which we used for neural analyses – was the median of this posterior distribution. The four four primary behavioral variables extracted were: Contralateral (QContra) and Ipsilateral (QIpsi) Action Values, Relative (ΔQ) and State Value (V).

##### Action values (Figs. 2,4; Extended Data Figs. 4, 5, 8):

Contra- and Ipsilateral values were calculated using the following equation:

(Equation 21) $$Q^{R/L}=\beta_{rew}*Q_{rew}^{R/L}+\beta_{stay}*Q_{stay}^{R/L}$$

Where each value component ($Q_{\mathrm{rew}}^{R/L}$ and $Q_{\mathrm{stay}}^{R/L}$) were calculated using Equations 1–5. R or L was labelled Contralateral or Ipsilateral depending on the recording location (e.g. $Q^{R}$ was $Q^{\mathrm{Contra}}$ for recording in the left hemisphere).

##### Relative and State Value (Fig. 3; Extended Data Fig. 7):

The Relative Value (ΔQ) represents the model’s subjective preference for one choice over the other, incorporating both reward-based value and perseverative tendencies. It was calculated as:

(Equation 22) $${\text{Δ}}_{Q_{t}}=\beta_{rew}\cdot \text{Δ}Q_{rew_{t}}+\beta_{stay}\cdot \text{Δ}Q_{stay_{t}}$$

The components $\Delta Q_{{\mathrm{rew}}_{t}}$ (difference in reward-based Q-values) and $\Delta Q_{{\mathrm{stay}}_{t}}$ (difference in perseverative Q-values) were updated on every trial according to the rules described in the Q-learning model section (Equations 1–5).

The State Value (*V*) represents the model’s expectation of future reward from the current trial. This variable reflects the overall reward expectation in trial-by-trial bases. It was calculated as:

(Equation 23) $$\delta_{t}=R_{t}-V_{t}\;V_{t+1}\leftarrow V_{t}+\alpha_{rew}\cdot \delta_{t}$$

Where $R_{t}$ indicates if a reward was given a trial t and $V_{t}$ is initialized at 0.

#### Investigating the value of the CS+ (Extended Data Fig. 10):

To compare how mice adapted their behavior after choices resulting in the CS+ or the CS+ accompanied with VTA DA stimulation, we fitted a new version of Model 1 (Equations 1–6). In this version every update rule was maintained from the original model except for the governing of rewarded chosen options (Equation 1), which we instead modelled as follows:

- Outcome: CS+ with VTA DA stimulation
(Equation 24) $$Q_{rew_{t+1}}^{a_{t}}\leftarrow Q_{rew_{t}}^{a_{t}}+\alpha_{rew}\left(1-Q_{rew_{t}}^{a_{t}}\right)$$
- Outcome: CS+ without VTA DA stimulation
(Equation 25) $$Q_{rew_{t+1}}^{a_{t}}\leftarrow Q_{rew_{t}}^{a_{t}}+\alpha_{rew}\left(\kappa -Q_{rew_{t}}^{a_{t}}\right)$$

Here κ was a fitted free parameter representing the value of the CS+ in the absence of VTA DA stimulation. The prior for this parameter was set to κ∼N(0,1),κ∈R. Since not all sessions had trials with uncollected VTA DA stimulation rewards, for this analysis we only fitted sessions with at least 5 trials resulting in a CS+ without VTA DA stimulation. We then compared how close the posterior estimate for κ in each session was to 1 (preset value of CS+ with VTA DA stimulation) and away from 0 (preset value for trials resulting in a CS−, Equation 2) .

## NEUROPIXELS RECORDINGS

### Recording strategy:

We targeted all major striatal subregions and their associated cortical inputs and pallidal outputs. Recordings were done bilaterally, maximizing the number of distinct simultaneously recorded regions. To maximize the number of simultaneously inserted probes, all insertions were made with a combination of 10°, 15° and 30° zenith angles from probes positioned at 0°, 45°, 135° and 180° azimuth. Recording craniotomies (0.5 mm radius) across animals spanned from −1 to 3 mm A-P, and ± 0.2 - 4 mm M-L. Up to three recording sessions were performed per craniotomy; for each new session, the probe insertion site was moved by approximately 300 μm in the M-L and A-P axes, allowing us to easily associate probe histological reconstruction to recording/sessions. See Extended Data Table 4 for a summary of all craniotomies and recording trajectories tried and Extended Data Table 5 for a summary of regions targeted in each subject.

### Craniotomies:

On the day prior to the first recording, animals were anesthetized, head-fixed and the UV glue covering the skull was carefully removed with a scalpel. The skull surface was cleaned with saline and dried with surgical cotton tipped applicators. Small craniotomies were made over the desired recording locations with a dental drill (¼ drill bit). No durotomy was performed. Craniotomies were covered with a small layer of Neomycin antibiotic ointment (Thermofisher) and the skull surface was then covered with a silicone elastomer (Kwikcast or Kwikseal, WPI). Up to 4 craniotomies were performed in each animal. Craniotomies could be penetrated with Neuropixels probes for up to 4 days. After this point, the dura hardened, preventing new insertions. Therefore, depending on the desired recording locations, craniotomy surgeries were separated in time (4-5 days apart) to maximize the amount of recordings possible per animal. A maximum of three craniotomy surgeries were made in each mouse^133,148,149^.

### Neuropixels insertion and recordings:

All probes were grounded to the same ground pin that had been fixed during the headplating surgery into the cerebellum. Probes were connected to a Neuropixels PXI board (IMEC). The probes generated a 1 Hz square pulse sent to a I/O PXI module (National Instruments) to synchronize to behavioral and video streams. Recordings were acquired with the SpikeGLX software at 30 kHz. If noise levels were high (RMS >20 μV), grounding was set to internal referencing from the tip of the probes. All recordings were from the bottom 384 channels of each probe.

On each recording day, the tips of Neuropixels 1.0 probes (IMEC) were dipped in a drop of CM-DiI (1 μg/μl in ethanol) as previously described ^150^. Animals were head-fixed, the silicone elastomer was removed from the skull, and the craniotomies were rinsed with saline. Up to 4 probes targeting both hemispheres were inserted in each session. To avoid obstructing the visual field of adjacent craniotomies, probes were inserted in a two-stage process. First, all probes were lowered sequentially to a depth of ~1 mm. Subsequently, each probe was advanced individually to its final target depth. Each probe was lowered into the brain at a speed of 2-5 μm/s using a micromanipulator system (uMp, Sensapex). Once all probes reached their final depth, to prevent the craniotomies from drying out and to further stabilize the probe, the skull was covered with Dow-Sil to fill the craniotomies and engulf the probe shanks. To minimize drift during the recording session, the probes were left in position at their final depth for 5-10 min before the start of the recording. After this waiting period, recording and the behavioral task (probabilistic reversal learning task) started, which lasted 60-90 minutes. At the end of the recording session, the probes were carefully and sequentially removed at a speed of ~ 100 μm/s, the skull was cleaned, neomycin gel reapplied to the craniotomies and the skull was resealed with silicone elastomer.

### Spike sorting and manual curation:

We used the standardized IBL pipeline for pre-processing and spike sorting ^133,151^. Briefly, raw electrophysiological recordings underwent three main pre-processing steps:

1. *Correction for ‘sample shift’ along the length of the probe by aligning the samples with a frequency domain approach*: This correction accounts for the small lag in between the sampling of each channel.
2. *Automatic detection, rejection and interpolation of failing channels on the probe*: Due to channel or tissue damage some probes channels are ‘dead’ or excessively noisy producing data that could corrupt the spike sorting procedure. This correction removes data from these channels.
3. *Application of a spatial ‘de-striping’ filter*: This correction removes noise common to several channels by applying a high-pass spatial DC filter prior to spike sorting.

After these preprocessing steps, spike sorting was performed using ibl-sorter^152^, a Python port of the Kilosort 2.5 algorithm^153^ that includes modifications to preprocessing. At this step, we applied registration, clustering, and spike deconvolution. Finally, data was manually curated with the Phy2 software (https://github.com/cortex-lab/phy). Manual curation focused on the removal of units that contained electrical noise or missing spikes (inferred based on a truncated distribution of spike amplitudes), and the splitting of clusters with clearly separable spike sources. Manual curation labels were then combined with automated metrics that differed for single units and multi-unit activity (MUAs).

### Inclusion criteria and pre-processing:

For single units, we required the following values of our automated metrics: firing rate ≥0.01 sp/s, amplitude >30 μV, refractory period violations <10%, and spiking spanning at least 90% of trials in the behavioral session (defined as the fraction of total trials covered by the time from a neuron’s first to last spike). Single units were used for all analyses.

For MUAs, we required the following values: firing rate ≥0.01 sp/s, amplitude >30 μV, spiking in >75% of 10 s bins across the entire recording. All firing rates were estimated using only the time bins covered by the event kernels in the encoding model (described below). MUAs were only included in decoding analyses.

For all neural analyses, we binned and normalized each single unit’s spiking activity. First, we discretized each neuron’s spiking into 10 ms time-bins for each trial of the behavioral session with the bin edges aligned to the onset of the go cue. We then identified all of the time-bins that are covered by the event kernels in the encoding model (described below) and calculated the mean, *μ*, and standard deviation, *σ*, across those time-bins. We then *z*-scored the spiking activity for each neuron as:

(Equation 26) $$Sp(t,T)=\frac{sp(t,T)-\mu }{\sigma }$$

where sp(t,T) is the raw binned spiking activity at time t on trial T and Sp(t,T) is the normalized binned spiking activity.

For all neural analyses, we excluded the first and last ten trials of each behavioral session as well as any trials on which the animal did not make a choice or for which the time of wheel movement onset could not be identified post-hoc (described below).

For all neural analyses we used the raw firing rates from each neuron without applying any smoothing. For the PSTHs in Fig. 2.b,d and Fig. 3b,c, we applied a causal half-Gaussian filter with a 50 ms standard deviation to the neurons’ firing rates binned at 10 ms.

## NEURAL ANALYSES

### Neural encoding model

#### Basic encoding model:

To determine how activity of single units was modulated by task events (Figs. 2–4 and Extended Data Figs. 3–5,7–9), we built linear encoding models to relate neural activity to each event^154,155^. We used multiple linear regression analyses of each neuron with the *z*-scored 10 ms binned spiking activity as the dependent variable and behavioral events as the independent variables. To account for temporal offsets in the relationship between changes in spiking and behavioral events, independent variables were generated by convolving linearly spaced cosine basis sets (1 cosine per 25 ms of kernel duration) with binary vectors of event times (1 at the time of the behavioral event and 0 otherwise). To account for spike history effects^156^, we also included each neuron’s spike history as a set of additional independent variables that were generated by convolving a cosine basis set with log-scaling of the time axis (10 cosines spanning from 10 ms to 1 s in the past) with each neuron’s *z*-scored spiking activity. The cosine basis sets were generated with the MATLAB *raisedCosineBasis* package (https://github.com/pillowlab/raisedCosineBasis). The full model is:

(Equation 27) $$Sp(t,T)=\beta_{0}+\sum_{i=1}^{n_{\text{events}}}\sum_{j=1}^{n_{\text{dof}}}\beta_{ij}S_{j}(t)*c_{i}(t,T)+\sum_{k=1}^{n_{\text{dof}}}\beta_{k}S_{k}(t)*Sp(t,T)+\epsilon$$

where Sp(t,T) is the z-scored spiking activity at time t on trial $T,\beta_{0}$ is the intercept, ϵ is the noise, $n_{\text{events}}$ is the number of behavioral events, $\beta_{ij}$ is the regression coefficient for the *j*^th^ cosine basis function and *i*^th^ event, $S_{j}$ is the *j*^th^ cosine basis function, $n_{\text{dof}}$ is the number of cosines basis functions (i.e., degrees of freedom) for the *i*^th^ event, $c_{i}(t,T)$ is a binary vector the same length as Sp(t,T) that is 1 at the time of event i and 0 otherwise, $\beta_{k}$ is the regression coefficient for the *k*^th^ cosine basis function of the spike history kernel, $S_{k}$ is the *k*^th^ cosine basis function of the spike history kernel, and ∗ represents the convolution operation. Thus, spiking activity is modeled as the convolution of the event time vector $c_{i}(t,T)$, with a temporal kernel, $K_{i}(t)$, summed across events:

(Equation 28) $$Sp(t,T)=\beta_{0}+\sum_{i=1}^{n_{\text{events}}}K_{i}(t)*c_{i}(t,T)+K_{sp}(t)*Sp(t,T)+\epsilon$$

where the temporal kernel for event i is defined as:

(Equation 29) $$K_{i}(t)=\sum_{j=1}^{n_{\text{dof}}}\beta_{ij}S_{j}(t)$$

and the spike history kernel, $K_{sp}(t)$, is defined analogously.

We included temporal kernels for the following behavioral events: go cue (all trials), left movement onset (only left choice trials), right movement onset (only right choice trials), CS+ (only trials with CS+), laser stimulation onset (only trials with CS+ and laser stimulation), and CS− (only trials with CS−). The kernel for the go cue spanned from 0 to +1 sec. The kernels for left and right movement onset spanned from −0.5 s to +0.5 s. The kernels for CS+, laser stimulation onset, and CS− spanned from 0 to +2 sec. We estimated these temporal kernels with linear regression and lasso ($\ell_{1}$) regularization using the MATLAB *glmnet* package^157^ (https://github.com/junyangq/glmnet-matlab). The regularization parameter, λ, was chosen from a logarithmically spaced vector of 251 values from 10^−5^ to 10^5^ based on the minimum mean squared error using five-fold cross validation. For all encoding model analyses, trials were randomly assigned to cross validation folds with the constraint of approximately balancing block probability (left vs right high probability block), choice (left vs right), and reward (CS+ vs CS−) across folds.

We identified the onset of wheel movement using a strategy similar to ref. ^148^. First, we coarsely detected periods of movement as those with an average wheel speed (200 ms moving average) of ≥1 cm/sec. We next merged adjacent periods of movement that were separated by <100 ms and then removed periods of movement that were <50 ms duration. To precisely define movement onset times, for each movement detected above we identified the first moment at which the instantaneous wheel speed (5 ms moving average) was ≥0.2 cm/sec. The movement onset time for each trial was defined as the first movement onset time that occurred from 0.2 s before the time of the go cue to the time of the CS+ or CS−.

We classified choices and their associated Q-values as contralateral or ipsilateral with respect to each hemisphere of the brain based on the direction of wheel movement. For neurons on the left hemisphere, contralateral choices were defined as counterclockwise wheel movements (i.e., right choices) by the mouse and ipsilateral choices were defined as clockwise wheel movements (i.e., left choices). This is consistent with the definitions used for the IBL brain-wide map dataset^151^, which was collected in an identical recording and behavioral set up. Conversely, for recordings in the right hemisphere, contralateral choices were defined as clockwise wheel movements and ipsilateral choices were defined as counterclockwise wheel movements.

To identify significant modulation of neural activity by a particular event or set of events (Extended Data Fig. 3g–k; details for specific models below), we calculated an F-statistic comparing the fit of the full model to that of a reduced model refit without the predictors for that event. The F-statistic was calculated by comparing the predicted spiking activity, from five-fold cross validation, to the real spiking activity of each model:

(Equation 30) $$F=\frac{\text{ΔSSE}}{\text{ΔDOF}\;\cdot \;{\text{MSE}}_{\text{full}}}$$

where ΔSSE is the difference in sum squared error between the full and reduced models, ΔDOF is the difference in degrees of freedom (i.e., number of predictors) between the full and reduced models, and ${\mathrm{MSE}}_{\text{full}}$ is the mean squared error of the full model. Because the binned spiking data could be autocorrelated and therefore not i.i.d., we calculated P-values by comparing these F-statistics to those from a null distribution that preserved much of the same autocorrelation^89^. To do this, 200 instances of null spiking data were generated by circularly shifting the binned spiking activity from each session by a random integer (noting the problems with this described in ref. ^89^). ^89^e did not adopt a “pseudosession” approach^89,90^ here because the time-course of the neural responses to task events should be less susceptible to drift across a session (that may lead to spurious significance) than the trial-by-trial-modulation in amplitude or gain of the neural response (described below). P-values were calculated as the fraction of F-statistics from the null dataset that were greater than the real F-statistic. Neurons with P≤0.01 were considered to significantly encode that event.

We first used this strategy to identify all neurons that were significantly modulated by any task event. We compared the full model to a reduced model with all behavioral kernels removed and only the spike history kernel and intercept remaining. For all downstream encoding analyses, we only included neurons for which the full model was significantly better than this reduced model (Extended Data Fig. 3d; referred to as “task-modulated” in the main text; 4,036 out of 5,109 (79.0%) striatal single units).

We then used this strategy to classify neurons as significantly encoding (1) go cue (Extended Data Fig. 3g), by removing only the go cue kernel in the reduced model; (2) movement (Extended Data Fig. 3h), by removing both the left and right movement onset kernels; (3) outcome (Extended Data Fig. 3i), by removing the CS+, laser stimulation, and CS− kernels; (4) choice (Extended Data Fig. 3j), by replacing the left and right movement onset kernels with a single movement onset kernel that was not choice-specific; and (5) reward (Extended Data Fig. 3k), by replacing the CS+ and CS− kernels with a single CS kernel that was not reward-specific and also removing the laser stimulation onset kernel. We also used this strategy to specifically examine encoding of the CS+ by comparing the full model to a reduced model with only the CS+ kernel removed.

#### Bilinear encoding model:

To determine how the transient, event-related responses of striatal neurons were modulated by trial-by-trial fluctuations in value derived from the Q-learning model described above (Extended Q-learning model with the CS+ as reward), we fit bilinear models that include value-dependent multiplicative gains that scale the temporal kernels for each event on a trial-by-trial basis^44,82^. The trial-by-trial gain for event *i* is:

(Equation 31) $$G_{i}(T)=\beta_{i0}^{\text{gain}}+\sum_{k=1}^{n_{\text{gains}}}\beta_{ik}^{\text{gain}}U_{k}(T)$$

where $\beta_{i0}^{\text{gain}}$ is the gain offset for event $i,n_{\text{gains}}$ is the number of trial-by-trial variables, $\beta_{ik}^{\text{gain}}$ is the gain coefficient for trial-by-trial variable k and event i, and $U_{k}(T)$ is the value of trial-by-trial variable k (for example, ΔQ or V) on trial T. Thus, the spiking activity at time t on trial T is modeled as:

(Equation 32) $$Sp(t,T)=\beta_{0}+\sum_{i=1}^{n_{\text{events}}}G_{i}(T)K_{i}(t)*c_{i}(t,T)+K_{sp}(t)*Sp(t,T)+\epsilon$$

where $c_{i}(t,T)$ is a binary indicator of event times for event i and $K_{i}(t)$ and $K_{sp}(t)$ are the behavioral event and spike history kernels described above.

To fit the bilinear model defined in equation (Equation 32), we iteratively estimated the temporal kernels and intercept – $K_{i}(t),K_{sp}(t)$, and $\beta_{0}$ – and the value-dependent gains – $G_{i}(T)$. On each iteration, we first kept the value-dependent gains fixed and estimated the temporal kernels and intercept with linear regression and lasso ($\ell_{1}$) regularization using the MATLAB *glmnet* package. After fitting, the temporal kernels were normalized by dividing the coefficients for the cosine basis set, $\beta_{ij}$, by the Euclidean norm of the kernel. On the first iteration, $G_{i}(T)$ were all set to 1 and on subsequent iterations were based on the gain coefficients estimated in the previous iteration. We next kept the temporal kernels and intercept fixed and estimated the value-dependent gains with linear regression and ridge ($\ell_{2}$) regularization using the same MATLAB package. When fitting the value-dependent gains, the intercept, $\beta_{0}$, and spike history term, $K_{sp}(t)*Sp(t)$, fitted in the previous step were provided to the model as offset terms. The regularization parameters, λ, for both steps were chosen from a logarithmically spaced vector of 251 values from 10^−5^ to 10^5^ based on the minimum mean squared error using five-fold cross validation on the first iteration and then held constant at the same values for the remaining iterations. This process was repeated until the model converged (defined as three consecutive iterations for which every β changed by ≤0.001 compared to the previous iteration) or for 100 iterations.

We fit two separate bilinear models to evaluate the influence of contralateral and ipsilateral choice value (Fig. 2,4; Extended Data Fig. 4) or of relative and state value (Fig. 3; Extended Data Fig. 7,8) derived from the Extended Q-learning model with the CS+ as reward. In the contra/ipsi value encoding model (Fig. 2), the trial-by-trial variables were:

(Equation 33) $$Q^{contra/ipsi}(T)=\beta_{rew}Q_{rew}^{contra/ipsi}(T)+\beta_{stay}Q_{stay}^{contra/ipsi}(T)$$

In the relative/state value encoding model (Fig. 3), the trial-by-trial variables were:

(Equation 34) $$\text{Δ}Q(T)=\beta_{rew}\left(Q_{rew}^{contra}(T)-Q_{rew}^{ipsi}(T)\right)+\beta_{stay}\left(Q_{stay}^{contra}(T)-Q_{stay}^{ipsi}(T)\right)$$

and V(T) from equation (Equation 23). For both models, each gain was permitted to multiply every kernel and so there were a total of 18 predictors (2 trial-by-trial variable gains and 1 gain offset each for 6 kernels).

We next compared the full model with all trial-by-trial variable gains present to reduced models with some or all of these gains removed. For both the full and reduced models, the gains were refit using the kernels from the final iteration of the bilinear encoding model. The regularization parameter, λ, for ridge ($\ell_{2}$), was chosen independently for each model from a logarithmically spaced vector of 251 values from 10^−5^ to 10^5^ based on the minimum mean squared error using five-fold cross validation. For the bilinear model with $Q^{\mathrm{contra}}$ and $Q^{\mathrm{ipsi}}$, we fit reduced models with either or both of these trial-by-trial variables removed from every kernel (three reduced models total). For the bilinear model with ΔQ and V, we fit reduced models with either or both of these trial-by-trial variables removed from every kernel (three reduced models total). To quantify how strongly each neuron encoded a particular trial-by-trial variable or combination of variables, we calculated the difference in cross-validated variance explained, $\Delta R^{2}$, by the full model compared to the reduced models.

To identify significant modulation of neural activity by a particular trial-by-trial variable or combination of variables (Figs. 2–4; Extended Data Fig. 4,7,8), we calculated an F-statistic comparing the fit of the full and reduced models as in equation (Equation 30) and then compared this F-statistic to an appropriate null distribution. To generate the null distribution, we then calculated the same F-statistic for the same comparison after refitting the full bilinear model and reduced models for 200 instances of pseudosession data. Our goal was to preserve the temporal auto-correlations within the neural data as well as within the trial-by-trial value estimates — while breaking the temporal relationship between these two variables in the pseudosessions — to control for spurious correlations and significance in our encoding model that could potentially arise due to slow but unrelated drift in spiking and/or value^89,90^. These pseudosessions were created by concatenating the trial-by-trial variables from all behavioral sessions and then randomly selecting chunks of consecutive trials the same length as the real behavioral session; the event times from the real behavioral session were used when fitting the models. The same pseudosessions were used for every neuron. The regularization parameter, λ, for ridge ($\ell_{2}$) was chosen independently for each full or reduced model and for each instance of pseudosession data. P-values were calculated as the fraction of F-statistics from the null dataset that were greater than the real F-statistic. Neurons with P≤0.01 were considered to significantly encode that trial-by-trial variable or combination of variables. Similarly, we report the change in variance explained for each model comparison as corrected $\Delta R^{2}$, in which the mean $\Delta R^{2}$ across all pseudosessions in the null distribution is subtracted from the $\Delta R^{2}$ fit to the real behavioral data.

#### Learning rate analysis:

In Fig. 4, we used a variant of the bilinear encoding model to test how the learning rates in the behavioral model ($\alpha_{\mathrm{rew}}$ and $\gamma_{\mathrm{forget}}$) impact neural encoding of estimated trial-by-trial $Q_{\mathrm{rew}}^{\mathrm{contra}}$ and $Q_{\mathrm{rew}}^{\mathrm{ipsi}}$ values from the Extended Q-learning model with the CS+ as reward (Model 1). These learning rates only impact the reward and not stay component of the Q-values, so we considered only the contribution of reward to action value ($Q_{\mathrm{rew}}^{\mathrm{contra}/\mathrm{ipsi}}$) rather than the full estimate ($Q^{\mathrm{contra}/\mathrm{ipsi}}$ from Equation 34) in this encoding analysis.

First, we calculated $Q_{\mathrm{rew}}^{\mathrm{contra}}$ and $Q_{\mathrm{rew}}^{\mathrm{ipsi}}$, values for every trial of every session using Model 1 but with the $\alpha_{\mathrm{rew}}$ learning rate parameter set to a new value ($\gamma_{\mathrm{forget}}$ remains fixed at the original value). We then refit trial-by-trial gains for each neuron using the kernels from the final iteration of the bilinear encoding model described above but replacing the trial-by-trial $Q_{\mathrm{rew}}^{\mathrm{contra}}$ and $Q_{\mathrm{rew}}^{\mathrm{ipsi}}$ values with those refit using the new $\alpha_{\mathrm{rew}}$ value. The regularization parameter, λ, for ridge ($\ell_{2}$) was chosen independently for each value of the learning rate $\alpha_{\mathrm{rew}}$ from a logarithmically spaced vector of 251 values from 10^−5^ to 10^5^ based on the minimum mean squared error using five-fold cross validation. We also refit reduced models with either or both of the $\Delta Q_{\mathrm{rew}}$ and V gains removed for every kernel, refitting the regularization parameter independently for each reduced model. We then calculated the difference in cross-validated variance explained, $\Delta R^{2}$, for the model with either the $Q_{\mathrm{rew}}^{\mathrm{contra}}$ or the $Q_{\mathrm{rew}}^{\mathrm{ipsi}}$ gains present compared to the reduced model with no trial-by-trial gains. We then repeated this analysis for 40 linearly spaced values of $\alpha_{\mathrm{rew}}$ ranging from 0.025 to 1 (Fig. 4c,d, Extended Data Fig. 8a,b).

We used an analogous approach to test how varying $\gamma_{\mathrm{forget}}$ impacts neural encoding of the estimated trial-by-trial $Q_{\mathrm{rew}}^{\mathrm{contra}}$ and $Q_{\mathrm{rew}}^{\mathrm{ipsi}}$ values. In this case, we repeated the analysis above for 40 linearly spaced values of $\gamma_{\mathrm{forget}}$ ranging from 0.025 to 1 with $\alpha_{\mathrm{rew}}$ remaining fixed at the original value (Fig. 4e,f, Extended Data Fig. 8c,d).

We repeated this entire process for the relative/state value encoding model using the estimated trial-by-trial $\Delta Q_{\mathrm{rew}}$ and V values as gains (Extended Data Fig. 8e–g). Here we once again considered only the contribution of reward, and not the full estimate including the stay parameter.

We found that estimates of $V,Q_{\mathrm{rew}}^{\mathrm{contra}}$ , and $Q_{\mathrm{rew}}^{\mathrm{ipsi}}$ each monotonically increased throughout a behavioral session for low learning and forgetting rates, which could result in spurious correlations with slow but unrelated drift in spiking across a session. Therefore, for all encoding analyses above we detrended the raw estimates of $V,Q_{\mathrm{rew}}^{\mathrm{contra}}$, and $Q_{\mathrm{rew}}^{\mathrm{ipsi}}$ before fitting the encoding models. Specifically, we fit a linear regression of trial number (independent variable) and $V,Q_{\mathrm{rew}}^{\mathrm{contra}}$, or $Q_{\mathrm{rew}}^{\mathrm{ipsi}}$ (dependent variable) using the MATLAB *fitlm* function and then used the residuals from this model as the trial-by-trial variable in the encoding models described above. We performed this operation separately for $V,Q_{\mathrm{rew}}^{\mathrm{contra}}$, and $Q_{\mathrm{rew}}^{\mathrm{ipsi}}$ derived from each value of $\alpha_{\mathrm{rew}}$ or $\gamma_{\mathrm{forget}}$.

We used a pseudosession approach, analogous to that described above for the bilinear encoding model, to control for spurious correlations and significance in our encoding model that could potentially arise due to slow but unrelated drift in spiking and/or value^89,90^. These pseudosessions were created by concatenating the trial-by-trial variables from all behavioral sessions and then randomly selecting chunks of consecutive trials the same length as the real behavioral session. The same trial order was used for the pseudosession for each value of $\alpha_{\mathrm{rew}}$ and $\gamma_{\mathrm{forget}}$. We then report the change in variance explained for each model comparison as corrected $\Delta R^{2}$, in which the mean $\Delta R^{2}$ across all pseudosessions in the null distribution is subtracted from the $\Delta R^{2}$ fit to the real behavioral data.

#### Statistical comparisons across brain regions:

We used the matlab *chi2cont* function (https://www.mathworks.com/matlabcentral/fileexchange/45203-chi2cont) to compare the proportion of significantly value-modulated neurons across pairs of brain regions (Fig. 2g–i and Fig. 3d,e) or within M/L or D/V bins of intermediate CP (Fig. 3j) with $x^{2}$ tests. We used the built-in matlab *signrank* function to compare the strength ($\Delta R^{2}$) of value-modulation across intermediate and caudal CP (Extended Data Fig. 4a and Extended Data Fig. 7e) or within M/L or D/V bins of intermediate CP (Extended Data Fig. 7m) with Mann-Whitney U tests. Detailed results of all statistical comparisons are shown in Extended Data Table 7.

### Neural decoders

To investigate the neural representation of the modeled behavioral variables, we employed a decoding approach. We trained linear decoders to predict four key variables derived from our computational models: Contralateral choice value ($Q^{\mathrm{contra}}$) and Ipsilateral choice value ($Q^{\mathrm{ipsi}}$) (Fig. 2j–n; Extended Data Fig. 4f–i,5) or Relative Value (ΔQ) and State Value (V) (Fig. 3f,g; Extended Data Fig. 7i,j).

Decoders were trained using varying numbers of neurons (curated units and multiunits) to assess the robustness of the neural encoding, with combinations of 10, 20, 30 and 40 neurons. For each n neurons decoder, the decoding procedure was run 100 times on random combinations of neurons and summarized by its mean performance. All neurons selected for a given decoder were drawn from simultaneously recorded neurons from the same striatal or cortical subregion (for subregion designation for all decoding analyses please see Extended Data Table 3). Neural activity was binned into 100 ms intervals for decoding, with a separate decoder trained for each time bin. The temporal windows for decoding were chosen to align with relevant task events: for State Value (V), neural activity was analyzed from 0.5 s *before* the outcome event to 2 s after the outcome. Critically, we predicted the pre-update value (i.e., the value before the reward was obtained on that trial). For Relative Value (ΔQ), activity was analyzed from 0.5 s *before* the Go cue to 0.5 s *after* the Go cue.

To mitigate potential confounds arising from the inherent correlation of Relative Value with choice and State Value with outcome, only trials where the mouse made a contralateral choice were included for Relative Value decoding and only rewarded trials were used for State Value decoding.

Our decoders predicted trial by trial value at each time point based on the population activity using linear regression with Lasso (L1) regularization and an intercept. We performed 5-fold nested stratified cross-validation ^158^ to evaluate model performance and optimize regularization strength (λ) for each decoder (i.e., for each time bin and neuron combination). The procedure consists of an inner and outer loop both having 5 stratified folds. The outer folds are used for model evaluation and the inner folds for λ selection (λ = {10^−6^, 10^−5^, 10^−4^, 10^−3^, 10^−2^, 10^−1^, 10^0^, 10^1^). Briefly, (i) trials were first discretized into bins of uniform width using a Freedman-Diaconis rule based on the trials value (e.g. $Q^{\mathrm{contra}}$). Once trials were divided in bins, (ii) trials within each bin were shuffled and (iii) divided into 5 outer folds with a similar number of trials from each bin. One fold serves as a test set for evaluation and the other four make up the training set. (iv) For each outer training set, an inner cross-validation procedure is used to select an optimal λ. This inner cross-validation procedure also consists of 5 folds. A decoder was trained with a specific λ on 4 of the inner folds and validated on the remaining inner fold. This was repeated 5 times, and the average validation score across the 5 inner folds is stored. This was repeated for every tested λ. From this, a winner λ (best R^2^) is selected. (v) Finally, the model is trained with the winner λ in the entire outer training set and evaluated on the held-out outer test set. Steps iv-v are repeated 4 more times for the remaining outer fold sets. The final reported performance is the average score across the 5 outer test sets.

To account for nonsense correlations ^89^ we corrected the R^2^ values reported in all time-resolved decoders (Fig. 2k–n, Fig. 3f,g, Extended Data Fig. 5.) by subtracting the mean $R^{2}$ obtained from decoding a null distribution consisting of 200 “pseudosessions” from the R^2^ on the real session^151,159^. These pseudosessions were created by concatenating all actual Qcontra, Qipsi or State or Relative Values from all experimental sessions and then randomly selecting chunks of the same length as the original session data. To compare performance across regions (Fig. 2k–n, Fig. 3f,g) we statistically compared the corrected $R^{2}s$ obtained from running the decoders in NAc vs all other CP subregions.

## ALLEN ATLAS PROJECTION INTENSITY ANALYSIS:

For Fig. 3 k–m, we analyzed cortical viral injection experiments and projection images from the Allen Institute’s Mouse Connectivity Atlas (http://connectivity.brain-map.org/) ^95^ to determine which cortical areas projected to different striatal subregions and domains, and to correlate those patterns with the encoding of state and action value.

We queried the API for experiments from wild-type mice (n = 58) and several Cre lines targeting specific cortical layers. In selecting Cre lines, we focused on lines that target layer 5 and layers 2/3. Lines that instead target layer 4 and layer 6 were excluded because these layers do not project to striatum ^95,160^. The Cre lines analyzed were: A930038C07Rik-Tg1-Cre (targets layer 5b neurons, n = 30) ^161^, Rbp4-Cre_KL100 (targets layer 5 pyramidal neurons, n = 43 experiments) ^95,162^, Tlx3-Cre_PL56 (targets layer 5a IT neurons, n = 27 experiments) ^95,162^, Cux2-IRES-Cre (targets layers 2/3 and 4, n = 39 experiments) ^95,163^, Emx1-IRES-Cre (targets cortical excitatory neurons, n = 8 experiments) ^95,164^, and Grp-Cre_KH288 (targets layers 2/3 and 4, n = 14 experiments) ^95,162^. The full list and details of used experiments selected for analysis can be found in Extended data table 6.

For each experiment, we first normalized projection intensity by dividing by the total injection intensity in the source cortical structure. Specifically, we used data provided by the Allen Institute to normalize: the value ‘sum_pixel_intensity’ given by structure_unionizes for the injection region (https://allensdk.readthedocs.io/en/latest/unionizes.html). We grouped injections together by region (PFC, ACC, OFC, Insula, SS and MO), and averaged across experiments.

For Fig. 3k, we then scaled the mean projection intensity from 0 to 1 at the voxel level on this average data for each source region and subdivided the striatum into the 31 different domains. For each striatal domain, we averaged the normalized mean projection intensity in that subregion’s voxels. For each cortical area, we then quantified the correlation (Pearson *r*) between the mean projection intensity in each domain versus the percentage of neurons significantly encoding state or relative value in each domain for which we had recorded at least 20 neurons (n = 25 domains).

For Fig. 3l,m visualizations of projections from source data in MO and OFC, we averaged across allen atlas slices at the AP locations 48, 49 and 50 ARA (spanning 200 μm), corresponding to the subregion CP-int.

## FIBER PHOTOMETRY RECORDINGS:

We simultaneously recorded rGRAB-DA^165^ signals from dorsolateral striatum (DLS) and nucleus accumbens core (NAcc) with a multi-fiber photometry system (FP3002, Neurophotometrics) at the same time we stimulated VTA DA cell bodies expressing ChR2 in naive mice not performing any task (Extended Data Fig. 6). We waited a minimum of 1 month from viral injection for expression before starting photometry recordings. Stimulation and imaging were controlled with the Bonsai Neurophotometrics module. The photometry system consisted of an LED for excitation (560 nm light of 10 ms width pulses at 20 Hz) and a CMOS camera acquiring fluorescence emissions. The camera acquisition epochs were synchronous with the LED flashes. We used a low autofluorescence patch cord with 3 bundled branches (∅200/220 mm fiber core/cladding diameter, 0.37 NA, 1.25 mm ferrule diameter, Doric) to be able to image DLS and NAcc simultaneously (one branch was unused). On the recording day and prior to the recording, we passed 0.5 mW 470 nm light through the patch cord for 1 hour in order to photobleach autofluorescence within the patch cord, and improve recording quality. 1s light trains of optogenetic stimulation (20 Hz, 8 mW, 447 nm, 5ms pulse width) was delivered bilaterally with a separate patch cord (∅200/220 mm fiber core/cladding diameter, 0.39 NA, 2.5 mm ferrule diameter, Doric) connected to optical fibers located over the VTA (0.39 NA, ∅300 mm core, 2.5 mm ferrule, Thorlabs). To prevent optical bleedthrough from the stimulation light to the recording system, imaging and stimulation were interleaved such that optogenetic stimulation occurred in the periods that the CMOS was not acquiring

Fluorescence signals recorded during each session from each location were transformed to ΔF/F_0_ using the following formula:

(Equation 34) $$\frac{\text{Δ}F}{F}=\frac{F-F_{0}}{F_{0}}$$

F_0_ signal was the ± 30 s rolling average of the raw fluorescence signal. Finally, ΔF/F_0_ signals were z-scored per-session, using a mean and standard deviation calculated based on all the data from each session.

## HISTOLOGY:

Mice were anesthetized with pentobarbital sodium (2 mg/kg, Euthasol) and transcardially perfused first with 10 ml of ice-cold phosphate buffered saline (PBS) followed by 30 ml of 4% paraformaldehyde (PFA) in PBS. Brains were then dissected and post-fixed in 4% PFA overnight at 4°C. After fixation, brains were either: a) shipped to the Sainsbury Wellcome Centre for whole brain serial sectioning 2-photon microscope (for Neuropixels experiments in Figs. 1–5, Extended Data Figs. 1–5,7–10) or b) imaged with an automated slide scanner (NanoZoomer S60, Hamamatsu) (for photometry experiments in Extended Data Fig. 6).

### Whole-brain serial two photon imaging:

Brains were embedded in agarose and then serially sectioned and imaged using a 4 kHz resonant scanning two-photon system immersed in 50 mM PBS ^166,167^. The microscope was operated with ScanImage Basic (Vidrio Technologies, USA), and imaging parameters were configured through BakingTray, a custom-developed software (https://github.com/SWC-Advanced-Microscopy/BakingTray). Individual image segments were stitched together into 2D planes with StitchIt (https://github.com/SWC-Advanced-Microscopy/StitchIt). Complete coronal brain image stacks were imaged at a resolution of 4.4 x 4.4 x 25.0 μm (XYZ) using a Nikon 16x objective (NA 0.8) and a 920 nm laser with approximately 150 mW power at the tissue. A vibrating blade microtome (VT1000 vibratome, Leica) created 50 μm slices. Optical imaging within each slice captured two focal planes at depths around 30 μm and 55 μm from the top surface with a piezo objective scanner (PIFOC, PI). Two fluorescence channels were simultaneously recorded using Hamamatsu R10699 multialkali PMTs: ‘Green’ (525 nm center, 50 nm bandwidth; Chroma ET525/50m) and ‘Red’ (570 nm long-pass filter; Chroma ET570lp).

### Registration and localization of Neuropixels probes and optical fibers from whole-brain stacks:

The obtained whole brain images from serial two-photon imaging were first downsampled to 25 μm isotropic voxels. Then, they were spatially registered to the adult mouse Allen Common Coordinate Framework ^168^ via BrainRegister (https://github.com/stevenjwest/brainregister), a registration procedure based on elastix ^169^ with optimized settings for mouse brain data. Samples were registered to the CCF template image and the CCF template was also registered to the sample.

Neuropixels probe tracks were then manually traced on image stacks registered to the Allen CCF using the python package Lasagna (https://github.com/SainsburyWellcomeCentre/lasagna). Tracing was performed on merged stacks of the autofluorescence and CM-DiI channel using coronal and horizontal views. Neuropixels channels were mapped to the traced tracks (now in Allen CCF space) and further aligned based on electrophysiological features using a custom alignment tool ^170^.

While the Allen CCF groups all caudoputamen regions into a single entity, we required finer anatomical resolution for precise localization of our recording sites. To achieve this, we utilized a striatum atlas ^92^, which is based on the cortico-striatal projectome ^91^. This specialized atlas, also defined within the AllenCCF space, subdivides the caudoputamen into 27 distinct CP “domains”. These domains are further organized into “subregions” (Rostral, Intermediate, and Caudal) based on their anterior-posterior positions (for details see Fig. 2e, Extended Data Fig. 3). To assign our Neuropixels recording channels to these domains, we located each channel’s closest 100 μm coronal slice in the specialized striatal atlas and assigned the corresponding anatomical label for that M-L x D-V location. As both atlases are in AllenCCF space, no further alignment was necessary.

For locating optical fibers in mice doing the probabilistic learning task (Extended Data Fig. 1), optical fiber location was determined as the coordinates of the center of the lesion tip in the whole-brain stacks registered to the AllenCCF.

### Cryostat sectioning and Slide scanning (Nanozoomer):

Brains were dehydrated in 30% sucrose for cryoprotection. The brain was mounted within OCT and cryosectioned in 50 μm slices with a cryostat. Sections were mounted with DAPI Fluoromount-G (Southern Biotech) and imaged with an automated slide scanner (NanoZoomer S60, Hamamatsu).

### Localization of optical fibers from Nanozoomer stacks:

Coronal cryosections sections containing the most ventral lesion made by the optical fiber were manually aligned to the striatum atlas (see previous section). Alignment was based on anatomical landmarks such as the thickness and shape of the corpus callosum and size of the lateral ventricles. The lesion tip was used as the reference coordinates for fiber or injection location.

## STATISTICS:

No statistical methods were used to predetermine sample sizes. Sample sizes were chosen based on previous studies^55,82,148^ and on the availability of animals. Replication: All attempts at replication were successful. Most experiments were replicated in multiple independent cohorts of animals with all experimental groups present in each cohort. We used multiple independent experimental approaches, and multiple independent analyses within each experiment, to confirm our findings whenever possible. Randomization: For experiments with multiple groups, individual animals or entire cages were randomly assigned to a group either at the time of surgery or at the beginning of behavioral testing Blinding: Automated analyses and experimental hardware and software, without manual intervention, were used whenever possible. Experimenters were not blinded to the group assignments of the animals. Statistical tests and data analysis were performed in MATLAB (R2021a), Stan (2.36) and Python (3.8.10) as described above.. Individual data points are shown when practical (and always for n ≤ 10), and box plots show the data distribution for larger sample sizes. Sample sizes, statistical tests, exact P-values, error bars, shading, and box plots are defined in the figure captions, Methods, and Extended Data Table 7.

### Single neuron statistics:

To determine if neurons are significantly modulated by *any* task event, we first compared the full linear encoding model for each neuron to a reduced model with all behavioral kernels removed. We then used a similar strategy to classify neurons as significantly encoding *individual* task events, by comparing the full linear encoding model for each neuron to reduced models with the relevant behavioral kernels (e.g., the go cue kernel or the action kernels) removed. For each statistical test, we compared the *F*-statistic from modeling the real spiking and behavioral data to a null distribution of *F*-statistics that was created for each neuron using circularly shifted spiking data. We considered neurons with *P* ≤ 0.01 to be significantly modulated by a given task event. See the “Basic encoding model” section of the neural encoding model Methods for details.

To determine if neurons were significantly modulated (P≤0.01) by trial-by-trial estimates of value, we compared the full bilinear encoding model for each neuron to reduced models with the relevant trial-by-trial gains (e.g., $Q^{\mathrm{contra}}$ and/or $Q^{\mathrm{ipsi}}$) removed. For each statistical test, we compared the F-statistic from modeling the real spiking and behavioral data to a null distribution of F-statistics that was created for each neuron using pseudosessions of behavioral data. We considered neurons with P≤0.01 to significantly encode value. See the “Bilinear encoding model” section of the neural encoding model Methods for details.

All other statistical comparisons are described in the “Statistical comparisons across brain regions” section of the neural encoding model Methods.

### Decoding model statistics:

To statistically compare the decoding performance for the different studied value variables (e.g. $Q^{\mathrm{contra}}$) between CP regions and the NAc, we compared the null corrected R^2^ (see Methods: Neural Decoders for details on the null correction) for every *Subregion* x *Session* x *Time point* using a Mann-Whitney U rank sum test. For comparing decoding performance for different value variables ($Q^{\mathrm{contra}}$ vs. $Q^{\mathrm{ipsi}}$) within a region, we used the same approach but using a paired Wilcoxon signed rank test instead.

## Extended Data

## Supplementary Material

Supplement 1

## Data Availability

All data is scheduled for public release on November 30th 2025 and will be available through the International Brain Laboratory interface and data repository (https://www.internationalbrainlab.com/data). Final code for every visualization and analysis will be released upon publication on GitHub.

## References

[1] Montague P. R., Dayan P. & Sejnowski T. J. A framework for mesencephalic dopamine systems based on predictive Hebbian learning. J. Neurosci. 16, 1936–1947 (1996). 8774460 10.1523/JNEUROSCI.16-05-01936.1996 PMC6578666

[2] Beckstead R. M., Domesick V. B. & Nauta W. J. Efferent connections of the substantia nigra and ventral tegmental area in the rat. Brain Res. 175, 191–217 (1979). 314832 10.1016/0006-8993(79)91001-1

[3] Swanson L. W. The projections of the ventral tegmental area and adjacent regions: a combined fluorescent retrograde tracer and immunofluorescence study in the rat. Brain Res. Bull. 9, 321–353 (1982). 6816390 10.1016/0361-9230(82)90145-9

[4] Schultz W., Dayan P. & Montague P. R. A neural substrate of prediction and reward. Science 275, 1593–1599 (1997). 9054347 10.1126/science.275.5306.1593

[5] Cohen J. Y., Haesler S., Vong L., Lowell B. B. & Uchida N. Neuron-type-specific signals for reward and punishment in the ventral tegmental area. Nature 482, 85–88 (2012). 22258508 10.1038/nature10754 PMC3271183

[6] Pan W.-X., Schmidt R., Wickens J. R. & Hyland B. I. Dopamine cells respond to predicted events during classical conditioning: evidence for eligibility traces in the reward-learning network. J. Neurosci. 25, 6235–6242 (2005). 15987953 10.1523/JNEUROSCI.1478-05.2005 PMC6725057

[7] Hart A. S., Rutledge R. B., Glimcher P. W. & Phillips P. E. M. Phasic dopamine release in the rat nucleus accumbens symmetrically encodes a reward prediction error term. J. Neurosci. 34, 698–704 (2014). 24431428 10.1523/JNEUROSCI.2489-13.2014 PMC3891951

[8] Gershman S. J. Explaining dopamine through prediction errors and beyond. Nat. Neurosci. 27, 1645–1655 (2024). 39054370 10.1038/s41593-024-01705-4

[9] Jiang L. & Litwin-Kumar A. Models of heterogeneous dopamine signaling in an insect learning and memory center. PLoS Comput. Biol. 17, e1009205 (2021). 34375329 10.1371/journal.pcbi.1009205 PMC8354444

[10] Steinberg E. E. Positive reinforcement mediated by midbrain dopamine neurons requires D1 and D2 receptor activation in the nucleus accumbens. PLoS One 9, e94771 (2014). 24733061 10.1371/journal.pone.0094771 PMC3986242

[11] Witten I. B. Recombinase-driver rat lines: tools, techniques, and optogenetic application to dopamine-mediated reinforcement. Neuron 72, 721–733 (2011). 22153370 10.1016/j.neuron.2011.10.028 PMC3282061

[12] Stauffer W. R. Dopamine Neuron-Specific Optogenetic Stimulation in Rhesus Macaques. Cell 166, 1564–1571.e6 (2016). 27610576 10.1016/j.cell.2016.08.024 PMC5018252

[13] Willmore L., Cameron C., Yang J., Witten I. B. & Falkner A. L. Behavioural and dopaminergic signatures of resilience. Nature 611, 124–132 (2022). 36261520 10.1038/s41586-022-05328-2 PMC10026178

[14] Covey D. P. & Cheer J. F. Accumbal dopamine release tracks the expectation of dopamine neuron-mediated reinforcement. Cell Rep. 27, 481–490.e3 (2019). 30970251 10.1016/j.celrep.2019.03.055 PMC6481661

[15] Hamid A. A. Mesolimbic dopamine signals the value of work. Nat Neurosci 19, 117–126 (2016). 26595651 10.1038/nn.4173 PMC4696912

[16] Fischbach S. & Janak P. H. Decreases in cued reward seeking after reward-paired inhibition of mesolimbic dopamine. Neuroscience 412, 259–269 (2019). 31029728 10.1016/j.neuroscience.2019.04.035 PMC6858844

[17] Parker N. F. Reward and choice encoding in terminals of midbrain dopamine neurons depends on striatal target. Nat. Neurosci. 19, 845–854 (2016). 27110917 10.1038/nn.4287 PMC4882228

[18] Chang C. Y. Brief optogenetic inhibition of dopamine neurons mimics endogenous negative reward prediction errors. Nat. Neurosci. 19, 111–116 (2016). 26642092 10.1038/nn.4191 PMC4696902

[19] Reynolds J. N. J. Coincidence of cholinergic pauses, dopaminergic activation and depolarisation of spiny projection neurons drives synaptic plasticity in the striatum. Nat Commun 13, 1296 (2022). 35277506 10.1038/s41467-022-28950-0 PMC8917208

[20] Reynolds J. N. J. & Wickens J. R. Dopamine-dependent plasticity of corticostriatal synapses. Neural Netw 15, 507–521 (2002). 12371508 10.1016/s0893-6080(02)00045-x

[21] Calabresi P., Picconi B., Tozzi A. & Di Filippo M. Dopamine-mediated regulation of corticostriatal synaptic plasticity. Trends Neurosci 30, 211–219 (2007). 17367873 10.1016/j.tins.2007.03.001

[22] Surmeier D. J., Plotkin J. & Shen W. Dopamine and synaptic plasticity in dorsal striatal circuits controlling action selection. Curr. Opin. Neurobiol. 19, 621–628 (2009). 19896832 10.1016/j.conb.2009.10.003 PMC2818437

[23] Cerovic M., d’Isa R., Tonini R. & Brambilla R. Molecular and cellular mechanisms of dopamine-mediated behavioral plasticity in the striatum. Neurobiol. Learn. Mem. 105, 63–80 (2013). 23827407 10.1016/j.nlm.2013.06.013

[24] Fuchsberger T. Dopamine increases protein synthesis in hippocampal neurons enabling dopamine-dependent LTP. (2025) doi:10.7554/eLife.100822. PMC11893101 40063079

[25] Molina-Luna K. Dopamine in motor cortex is necessary for skill learning and synaptic plasticity. PLoS One 4, e7082 (2009). 19759902 10.1371/journal.pone.0007082 PMC2738964

[26] Bao S., Chan V. T. & Merzenich M. M. Cortical remodelling induced by activity of ventral tegmental dopamine neurons. Nature 412, 79–83 (2001). 11452310 10.1038/35083586

[27] Macedo-Lima M. & Remage-Healey L. Dopamine modulation of motor and sensory cortical plasticity among vertebrates. Integr. Comp. Biol. 61, 316–336 (2021). 33822047 10.1093/icb/icab019 PMC8600016

[28] Handler A. Distinct dopamine receptor pathways underlie the temporal sensitivity of associative learning. Cell 178, 60–75.e19 (2019). 31230716 10.1016/j.cell.2019.05.040 PMC9012144

[29] Engel L. Dopamine neurons drive spatiotemporally heterogeneous striatal dopamine signals during learning. Curr. Biol. 34, 3086–3101.e4 (2024). 38925117 10.1016/j.cub.2024.05.069 PMC11279555

[30] Saunders B. T., Richard J. M., Margolis E. B. & Janak P. H. Dopamine neurons create Pavlovian conditioned stimuli with circuit-defined motivational properties. Nat. Neurosci. 21, 1072–1083 (2018). 30038277 10.1038/s41593-018-0191-4 PMC6082399

[31] Steinberg E. E. A causal link between prediction errors, dopamine neurons and learning. Nature Neuroscience vol. 16 966–973 Preprint at 10.1038/nn.3413 (2013). 23708143 PMC3705924

[32] Tsai H.-C. Phasic firing in dopaminergic neurons is sufficient for behavioral conditioning. Science 324, 1080–1084 (2009). 19389999 10.1126/science.1168878 PMC5262197

[33] Fraser K. M., Pribut H. J., Janak P. H. & Keiflin R. From prediction to action: Dissociable roles of ventral tegmental area and substantia nigra dopamine neurons in instrumental reinforcement. J. Neurosci. 43, 3895–3908 (2023). 37185097 10.1523/JNEUROSCI.0028-23.2023 PMC10217998

[34] Millard S. J. Cognitive representations of intracranial self-stimulation of midbrain dopamine neurons depend on stimulation frequency. Nat. Neurosci. 27, 1253–1259 (2024). 38741021 10.1038/s41593-024-01643-1 PMC11239488

[35] Shin E. J. Robust and distributed neural representation of action values. Elife 10, (2021). 10.7554/eLife.53045 PMC8104958 33876728

[36] Markowitz J. E. Spontaneous behaviour is structured by reinforcement without explicit reward. Nature 614, 108–117 (2023). 36653449 10.1038/s41586-022-05611-2 PMC9892006

[37] Hayden B. Y. & Platt M. L. Neurons in anterior cingulate cortex multiplex information about reward and action. J. Neurosci. 30, 3339–3346 (2010). 20203193 10.1523/JNEUROSCI.4874-09.2010 PMC2847481

[38] Samejima K., Ueda Y., Doya K. & Kimura M. Representation of action-specific reward values in the striatum. Science 310, 1337–1340 (2005). 16311337 10.1126/science.1115270

[39] O’Doherty J. Dissociable roles of ventral and dorsal striatum in instrumental conditioning. Science 304, 452–454 (2004). 15087550 10.1126/science.1094285

[40] Padoa-Schioppa C. & Assad J. A. Neurons in the orbitofrontal cortex encode economic value. Nature 441, 223–226 (2006). 16633341 10.1038/nature04676 PMC2630027

[41] Ballesta S., Shi W., Conen K. E. & Padoa-Schioppa C. Values encoded in orbitofrontal cortex are causally related to economic choices. Nature 588, 450–453 (2020). 33139951 10.1038/s41586-020-2880-x PMC7746614

[42] Kable J. W. & Glimcher P. W. The neural correlates of subjective value during intertemporal choice. Nat. Neurosci. 10, 1625–1633 (2007). 17982449 10.1038/nn2007 PMC2845395

[43] Paton J. J., Belova M. A., Morrison S. E. & Salzman C. D. The primate amygdala represents the positive and negative value of visual stimuli during learning. Nature 439, 865–870 (2006). 16482160 10.1038/nature04490 PMC2396495

[44] Lak A. Dopaminergic and prefrontal basis of learning from sensory confidence and reward value. Neuron 105, 700–711.e6 (2020). 31859030 10.1016/j.neuron.2019.11.018 PMC7031700

[45] Hattori R., Danskin B., Babic Z., Mlynaryk N. & Komiyama T. Area-specificity and plasticity of history-dependent value coding during learning. Cell 177, 1858–1872.e15 (2019). 31080067 10.1016/j.cell.2019.04.027 PMC6663310

[46] Ito M. & Doya K. Validation of decision-making models and analysis of decision variables in the rat basal ganglia. J. Neurosci. 29, 9861–9874 (2009). 19657038 10.1523/JNEUROSCI.6157-08.2009 PMC6666589

[47] FitzGerald T. H. B., Friston K. J. & Dolan R. J. Action-specific value signals in reward-related regions of the human brain. J. Neurosci. 32, 16417–23a (2012). 23152624 10.1523/JNEUROSCI.3254-12.2012 PMC3549268

[48] Cai X., Kim S. & Lee D. Heterogeneous coding of temporally discounted values in the dorsal and ventral striatum during intertemporal choice. Neuron 69, 170–182 (2011). 21220107 10.1016/j.neuron.2010.11.041 PMC3034314

[49] Ito M. & Doya K. Distinct neural representation in the dorsolateral, dorsomedial, and ventral parts of the striatum during fixed- and free-choice tasks. J. Neurosci. 35, 3499–3514 (2015). 25716849 10.1523/JNEUROSCI.1962-14.2015 PMC4339358

[50] Ito M. & Doya K. Parallel representation of value-based and finite state-based strategies in the ventral and dorsal striatum. PLoS Comput. Biol. 11, e1004540 (2015). 26529522 10.1371/journal.pcbi.1004540 PMC4631489

[51] Kim H., Sul J. H., Huh N., Lee D. & Jung M. W. Role of striatum in updating values of chosen actions. J. Neurosci. 29, 14701–14712 (2009). 19940165 10.1523/JNEUROSCI.2728-09.2009 PMC6666000

[52] Kim S., Cai X., Hwang J. & Lee D. Prefrontal and striatal activity related to values of objects and locations. Front. Neurosci. 6, 108 (2012). 22822390 10.3389/fnins.2012.00108 PMC3398315

[53] Lau B. & Glimcher P. W. Value representations in the primate striatum during matching behavior. Neuron 58, 451–463 (2008). 18466754 10.1016/j.neuron.2008.02.021 PMC2427158

[54] Wang A. Y., Miura K. & Uchida N. The dorsomedial striatum encodes net expected return, critical for energizing performance vigor. Nat. Neurosci. 16, 639–647 (2013). 23584742 10.1038/nn.3377 PMC3651899

[55] Ottenheimer D. J., Hjort M. M., Bowen A. J., Steinmetz N. A. & Stuber G. D. A stable, distributed code for cue value in mouse cortex during reward learning. eLife (2023) doi:10.7554/elife.84604.2. PMC10328514 37382590

[56] Engelhard B. Specialized coding of sensory, motor and cognitive variables in VTA dopamine neurons. Nature 570, 509–513 (2019). 31142844 10.1038/s41586-019-1261-9 PMC7147811

[57] Choi J. Y. A comparison of dopaminergic and cholinergic populations reveals unique contributions of VTA dopamine neurons to short-term memory. Cell Rep. 33, 108492 (2020). 33326775 10.1016/j.celrep.2020.108492 PMC8038523

[58] Lerner T. N. Intact-brain analyses reveal distinct information carried by SNc dopamine subcircuits. Cell 162, 635–647 (2015). 26232229 10.1016/j.cell.2015.07.014 PMC4790813

[59] Howe M. W. & Dombeck D. A. Rapid signalling in distinct dopaminergic axons during locomotion and reward. Nature 535, 505–510 (2016). 27398617 10.1038/nature18942 PMC4970879

[60] Kremer Y., Flakowski J., Rohner C. & Lüscher C. Context-dependent multiplexing by individual VTA dopamine neurons. J. Neurosci. 40, 7489–7509 (2020). 32859713 10.1523/JNEUROSCI.0502-20.2020 PMC7511185

[61] Verharen J. P. H., Zhu Y. & Lammel S. Aversion hot spots in the dopamine system. Curr. Opin. Neurobiol. 64, 46–52 (2020). 32146296 10.1016/j.conb.2020.02.002 PMC7483159

[62] Cai L. X. Distinct signals in medial and lateral VTA dopamine neurons modulate fear extinction at different times. Elife 9, (2020). 10.7554/eLife.54936 PMC7363446 32519951

[63] Hamid A. A., Frank M. J. & Moore C. I. Wave-like dopamine dynamics as a mechanism for spatiotemporal credit assignment. Cell 184, 2733–2749.e16 (2021). 33861952 10.1016/j.cell.2021.03.046 PMC8122079

[64] Mohebi A., Wei W., Pelattini L., Kim K. & Berke J. D. Dopamine transients follow a striatal gradient of reward time horizons. Nat. Neurosci. 27, 737–746 (2024). 38321294 10.1038/s41593-023-01566-3 PMC11001583

[65] Eshel N., Tian J., Bukwich M. & Uchida N. Dopamine neurons share common response function for reward prediction error. Nat. Neurosci. 19, 479–486 (2016). 26854803 10.1038/nn.4239 PMC4767554

[66] Menegas W., Akiti K., Amo R., Uchida N. & Watabe-Uchida M. Dopamine neurons projecting to the posterior striatum reinforce avoidance of threatening stimuli. Nat. Neurosci. 21, 1421–1430 (2018). 30177795 10.1038/s41593-018-0222-1 PMC6160326

[67] Greenstreet F. Action prediction error: a value-free dopaminergic teaching signal that drives stable learning. bioRxiv (2022) doi:10.1101/2022.09.12.507572.

[68] Moss M. M., Zatka-Haas P., Harris K. D., Carandini M. & Lak A. Dopamine Axons in Dorsal Striatum Encode Contralateral Visual Stimuli and Choices. J. Neurosci. 41, 7197–7205 (2021). 34253628 10.1523/JNEUROSCI.0490-21.2021 PMC8387116

[69] Sousa M. A multidimensional distributional map of future reward in dopamine neurons. Nature 642, 691–699 (2025). 40468078 10.1038/s41586-025-09089-6

[70] Coddington L. T., Lindo S. E. & Dudman J. T. Mesolimbic dopamine adapts the rate of learning from action. Nature 614, 294–302 (2023). 36653450 10.1038/s41586-022-05614-z PMC9908546

[71] Kishida K. T. Sub-second dopamine detection in human striatum. PLoS One 6, e23291 (2011). 21829726 10.1371/journal.pone.0023291 PMC3150430

[72] Phillips P. E. M., Robinson D. L., Stuber G. D., Carelli R. M. & Wightman R. M. Real-time measurements of phasic changes in extracellular dopamine concentration in freely moving rats by fast-scan cyclic voltammetry. Methods Mol. Med. 79, 443–464 (2003). 12506716 10.1385/1-59259-358-5:443

[73] Kuhr W. G. & Wightman R. M. Real-time measurement of dopamine release in rat brain. Brain Res. 381, 168–171 (1986). 3489505 10.1016/0006-8993(86)90707-9

[74] Wightman R. M., May L. J. & Michael A. C. Detection of dopamine dynamics in the brain. Anal. Chem. 60, 769A–779A (1988). 10.1021/ac00164a001 3063135

[75] Beyene M., Carelli R. M. & Wightman R. M. Cue-evoked dopamine release in the nucleus accumbens shell tracks reinforcer magnitude during intracranial self-stimulation. Neuroscience 169, 1682–1688 (2010). 20600644 10.1016/j.neuroscience.2010.06.047 PMC2918713

[76] Lak A., Stauffer W. R. & Schultz W. Dopamine prediction error responses integrate subjective value from different reward dimensions. Proc. Natl. Acad. Sci. U. S. A. 111, 2343–2348 (2014). 24453218 10.1073/pnas.1321596111 PMC3926061

[77] Kamath T. Hunger modulates exploration through suppression of dopamine signaling in the tail of the striatum. Neuron (2025) doi:10.1016/j.neuron.2025.09.009. PMC12712896 41043421

[78] Zolin A. Context-dependent representations of movement in Drosophila dopaminergic reinforcement pathways. Nat. Neurosci. 24, 1555–1566 (2021). 34697455 10.1038/s41593-021-00929-y PMC8556349

[79] Golden C. E. M. Estrogenic control of reward prediction errors and reinforcement learning. Neuroscience (2023).

[80] Howe M. W., Tierney P. L., Sandberg S. G., Phillips P. E. M. & Graybiel A. M. Prolonged dopamine signalling in striatum signals proximity and value of distant rewards. Nature 500, 575–579 (2013). 23913271 10.1038/nature12475 PMC3927840

[81] Phillips P. E. M., Stuber G. D., Heien M. L. A. V., Wightman R. M. & Carelli R. M. Subsecond dopamine release promotes cocaine seeking. Nature 422, 614–618 (2003). 12687000 10.1038/nature01476

[82] Cox J. A neural substrate of sex-dependent modulation of motivation. Nat. Neurosci. 26, 274–284 (2023). 36646878 10.1038/s41593-022-01229-9 PMC12232987

[83] Grossman C. D., Bari B. A. & Cohen J. Y. Serotonin neurons modulate learning rate through uncertainty. Curr. Biol. 32, 586–599.e7 (2022). 34936883 10.1016/j.cub.2021.12.006 PMC8825708

[84] Sutton R. S. & Barto A. G. Reinforcement Learning, Second Edition: An Introduction. (MIT Press, 2018).

[85] Carter F. Does phasic dopamine release cause policy updates? Eur. J. Neurosci. 59, 1260–1277 (2024). 38039083 10.1111/ejn.16199

[86] Wickens J. R., Begg A. J. & Arbuthnott G. W. Dopamine reverses the depression of rat corticostriatal synapses which normally follows high-frequency stimulation of cortex in vitro. Neuroscience 70, 1–5 (1996). 8848115 10.1016/0306-4522(95)00436-m

[87] Joel D., Niv Y. & Ruppin E. Actor-critic models of the basal ganglia: new anatomical and computational perspectives. Neural Netw. 15, 535–547 (2002). 12371510 10.1016/s0893-6080(02)00047-3

[88] Jun J. J. Fully integrated silicon probes for high-density recording of neural activity. Nature 551, 232–236 (2017). 29120427 10.1038/nature24636 PMC5955206

[89] Harris K. D. Nonsense correlations in neuroscience. bioRxiv (2020) doi:10.1101/2020.11.29.402719.

[90] Elber-Dorozko L. & Loewenstein Y. Striatal action-value neurons reconsidered. Elife 7, (2018). 10.7554/eLife.34248 PMC6008056 29848442

[91] Hintiryan H. The mouse cortico-striatal projectome. Nat. Neurosci. 19, 1100–1114 (2016). 27322419 10.1038/nn.4332 PMC5564682

[92] Chon U., Vanselow D. J., Cheng K. C. & Kim Y. Enhanced and unified anatomical labeling for a common mouse brain atlas. Nat. Commun. 10, 5067 (2019). 31699990 10.1038/s41467-019-13057-w PMC6838086

[93] Paninski L. Maximum likelihood estimation of cascade point-process neural encoding models. Network 15, 243–262 (2004). 15600233

[94] Paninski L., Pillow J. & Lewi J. Statistical models for neural encoding, decoding, and optimal stimulus design. Prog. Brain Res. 165, 493–507 (2007). 17925266 10.1016/S0079-6123(06)65031-0

[95] Oh S. W. A mesoscale connectome of the mouse brain. Nature 508, 207–214 (2014). 24695228 10.1038/nature13186 PMC5102064

[96] Erlich J. C., Bialek M. & Brody C. D. A cortical substrate for memory-guided orienting in the rat. Neuron 72, 330–343 (2011). 22017991 10.1016/j.neuron.2011.07.010 PMC3212026

[97] Siniscalchi M. J., Phoumthipphavong V., Ali F., Lozano M. & Kwan A. C. Fast and slow transitions in frontal ensemble activity during flexible sensorimotor behavior. Nat. Neurosci. 19, 1234–1242 (2016). 27399844 10.1038/nn.4342 PMC5003707

[98] Murakami M., Vicente M. I., Costa G. M. & Mainen Z. F. Neural antecedents of self-initiated actions in secondary motor cortex. Nat. Neurosci. 17, 1574–1582 (2014). 25262496 10.1038/nn.3826

[99] Li N., Chen T.-W., Guo Z. V., Gerfen C. R. & Svoboda K. A motor cortex circuit for motor planning and movement. Nature 519, 51–56 (2015). 25731172 10.1038/nature14178

[100] Sul J. H., Jo S., Lee D. & Jung M. W. Role of rodent secondary motor cortex in value-based action selection. Nat. Neurosci. 14, 1202–1208 (2011). 21841777 10.1038/nn.2881 PMC3164897

[101] Schall J. D., Stuphorn V. & Brown J. W. Monitoring and control of action by the frontal lobes. Neuron 36, 309–322 (2002). 12383784 10.1016/s0896-6273(02)00964-9

[102] Tanji J. & Evarts E. V. Anticipatory activity of motor cortex neurons in relation to direction of an intended movement. J. Neurophysiol. 39, 1062–1068 (1976). 824409 10.1152/jn.1976.39.5.1062

[103] Lawrence D. G. & Hopkins D. A. The development of motor control in the rhesus monkey: evidence concerning the role of corticomotoneuronal connections. Brain 99, 235–254 (1976). 825185 10.1093/brain/99.2.235

[104] Wise S. P. The primate premotor cortex: past, present, and preparatory. Annu. Rev. Neurosci. 8, 1–19 (1985). 3920943 10.1146/annurev.ne.08.030185.000245

[105] Lemon R. N. An enduring map of the motor cortex. Exp. Physiol. 93, 798–802 (2008). 18562475 10.1113/expphysiol.2007.039081

[106] Churchland M. M. Neural population dynamics during reaching. Nature 487, 51–56 (2012). 22722855 10.1038/nature11129 PMC3393826

[107] Hattori R. Meta-reinforcement learning via orbitofrontal cortex. Nat. Neurosci. 26, 2182–2191 (2023). 37957318 10.1038/s41593-023-01485-3 PMC10689244

[108] Stalnaker T. A., Raheja N. & Schoenbaum G. Orbitofrontal state representations are related to choice adaptations and reward predictions. J. Neurosci. 41, 1941–1951 (2021). 33446521 10.1523/JNEUROSCI.0753-20.2020 PMC7939081

[109] Wilson R. C., Takahashi Y. K., Schoenbaum G. & Niv Y. Orbitofrontal cortex as a cognitive map of task space. Neuron 81, 267–279 (2014). 24462094 10.1016/j.neuron.2013.11.005 PMC4001869

[110] Costa K. M. The role of the lateral orbitofrontal cortex in creating cognitive maps. Nat. Neurosci. 26, 107–115 (2023). 36550290 10.1038/s41593-022-01216-0 PMC9839657

[111] Tanaka S. C. Prediction of immediate and future rewards differentially recruits cortico-basal ganglia loops. Nat. Neurosci. 7, 887–893 (2004). 15235607 10.1038/nn1279

[112] Masset P. Multi-timescale reinforcement learning in the brain. Nature 642, 682–690 (2025). 40468072 10.1038/s41586-025-08929-9 PMC13254605

[113] Wilson R. C. & Niv Y. Is model fitting necessary for model-based fMRI? PLoS Comput. Biol. 11, e1004237 (2015). 26086934 10.1371/journal.pcbi.1004237 PMC4472514

[114] Campbell M. G. A hardwired neural circuit for temporal difference learning. bioRxiv (2025) doi:10.1101/2025.09.18.677203.

[115] Suri R. E. & Schultz W. A neural network model with dopamine-like reinforcement signal that learns a spatial delayed response task. Neuroscience 91, 871–890 (1999). 10391468 10.1016/s0306-4522(98)00697-6

[116] Wolff A. R. & Saunders B. T. Sensory cues potentiate VTA dopamine mediated reinforcement. eNeuro 11, ENEURO.0421–23.2024 (2024). 38238080 10.1523/ENEURO.0421-23.2024 PMC10875637

[117] Decot H. K. Coordination of brain-wide activity dynamics by dopaminergic neurons. Neuropsychopharmacology 42, 615–627 (2017). 27515791 10.1038/npp.2016.151 PMC5240174

[118] Lawen A. Dopamine release effects on striatal blood oxygenation and whole brain plasticity underlying associative learning. Neuroscience (2025).

[119] Joel D. & Weiner I. The connections of the dopaminergic system with the striatum in rats and primates: an analysis with respect to the functional and compartmental organization of the striatum. Neuroscience 96, 451–474 (2000). 10717427 10.1016/s0306-4522(99)00575-8

[120] Haber S. N. The primate basal ganglia: parallel and integrative networks. J. Chem. Neuroanat. 26, 317–330 (2003). 14729134 10.1016/j.jchemneu.2003.10.003

[121] Parkinson J. A., Robbins T. W. & Everitt B. J. Selective excitotoxic lesions of the nucleus accumbens core and shell differentially affect aversive Pavlovian conditioning to discrete and contextual cues. Psychobiology (Austin, Tex.) 27, 256–266 (1999).

[122] Day J. J. & Carelli R. M. The nucleus accumbens and Pavlovian reward learning. Neuroscientist 13, 148–159 (2007). 17404375 10.1177/1073858406295854 PMC3130622

[123] Saunders B. T. & Robinson T. E. The role of dopamine in the accumbens core in the expression of Pavlovian-conditioned responses. Eur. J. Neurosci. 36, 2521–2532 (2012). 22780554 10.1111/j.1460-9568.2012.08217.x PMC3424374

[124] Berridge K. C. The debate over dopamine’s role in reward: the case for incentive salience. Psychopharmacology (Berl.) 191, 391–431 (2007). 17072591 10.1007/s00213-006-0578-x

[125] McClure S. M., Daw N. D. & Montague P. R. A computational substrate for incentive salience. Trends Neurosci. 26, 423–428 (2003). 12900173 10.1016/s0166-2236(03)00177-2

[126] Ikemoto S. & Panksepp J. The role of nucleus accumbens dopamine in motivated behavior: a unifying interpretation with special reference to reward-seeking. Brain Res. Brain Res. Rev. 31, 6–41 (1999). 10611493 10.1016/s0165-0173(99)00023-5

[127] Coddington L. T. & Dudman J. T. Learning from action: Reconsidering movement signaling in midbrain dopamine neuron activity. Neuron 104, 63–77 (2019). 31600516 10.1016/j.neuron.2019.08.036

[128] Salamone J. D. Mesolimbic dopamine and the regulation of motivated behavior. Curr. Top. Behav. Neurosci. 27, 231–257 (2016). 26323245 10.1007/7854_2015_383

[129] Lex A. & Hauber W. Dopamine D1 and D2 receptors in the nucleus accumbens core and shell mediate Pavlovian-instrumental transfer. Learn. Mem. 15, 483–491 (2008). 18626092 10.1101/lm.978708 PMC2505315

[130] de Jong J. W. A neural circuit mechanism for encoding aversive stimuli in the mesolimbic dopamine system. Neuron 101, 133–151.e7 (2019). 30503173 10.1016/j.neuron.2018.11.005 PMC6317997

[131] International Brain Laboratory. A standardized and reproducible method to measure decision-making in mice: Appendix 1: IBL protocol for headbar implant surgery in mice. figshare (2020) doi:10.6084/m9.figshare.11634726.v1.

[132] International Brain Laboratory Standardized and reproducible measurement of decision-making in mice. Elife 10, (2021). 10.7554/eLife.63711 PMC8137147 34011433

[133] International Brain Laboratory Reproducibility of in vivo electrophysiological measurements in mice. eLife (2024) doi:10.7554/elife.100840.1. PMC12068871 40354112

[134] International Brain Laboratory. A standardized and reproducible method to measure decision-making in mice: Appendix 3: IBL protocol for setting up the behavioral training rig. 10.6084/m9.figshare.11634732.v1 (2020).

[135] International Brain Laboratory. Appendix 1: IBL electrophysiological (Ephys Neuropixels) rig setup instructions: Hardware and Software. figshare (2022) doi:10.6084/M9.FIGSHARE.19694446.V3.

[136] Lopes G. Creating and controlling visual environments using BonVision. Elife 10, (2021). 10.7554/eLife.65541 PMC8104957 33880991

[137] International Brain Laboratory. Video hardware and software for the International Brain Laboratory. Preprint at 10.6084/M9.FIGSHARE.19694452.V1 (2022).

[138] Watkins C. J. Learning from Delayed Rewards. (University of Cambridge, 1989).

[139] Watkins C. J. C. H. & Dayan P. Q-learning. Mach. Learn. 8, 279–292 (1992).

[140] Miller K. J., Botvinick M. M. & Brody C. D. From predictive models to cognitive models: Separable behavioral processes underlying reward learning in the rat. bioRxiv 461129 (2018) doi:10.1101/461129.

[141] Lebedeva A. Dorsal prefrontal cortex drives perseverative behavior in mice. bioRxiv (2024). 10.1038/s41467-026-71664-w PMC13324276 42034646

[142] Lee R. S., Mattar M. G., Parker N. F., Witten I. B. & Daw N. D. Reward prediction error does not explain movement selectivity in DMS-projecting dopamine neurons. Elife 8, (2019). 10.7554/eLife.42992 PMC6464606 30946008

[143] Gao J., Wong-Lin K., Holmes P., Simen P. & Cohen J. D. Sequential effects in two-choice reaction time tasks: decomposition and synthesis of mechanisms. Neural Comput. 21, 2407–2436 (2009). 19548803 10.1162/neco.2009.09-08-866 PMC2751646

[144] Williams R. J. Simple statistical gradient-following algorithms for connectionist reinforcement learning. Mach. Learn. 8, 229–256 (1992).

[145] Carpenter B. Stan: A probabilistic programming language. J. Stat. Softw. 76, 1–32 (2017). 36568334 10.18637/jss.v076.i01 PMC9788645

[146] Gelman A. & Rubin D. B. Inference from iterative simulation using multiple sequences. Stat. Sci. 7, 457–472 (1992).

[147] Wilson R. C. & Collins A. G. Ten simple rules for the computational modeling of behavioral data. Elife 8, (2019). 10.7554/eLife.49547 PMC6879303 31769410

[148] Steinmetz N. A., Zatka-Haas P., Carandini M. & Harris K. D. Distributed coding of choice, action and engagement across the mouse brain. Nature 576, 266–273 (2019). 31776518 10.1038/s41586-019-1787-x PMC6913580

[149] Chen S. Brain-wide neural activity underlying memory-guided movement. Cell 187, 676–691.e16 (2024). 38306983 10.1016/j.cell.2023.12.035 PMC11492138

[150] Liu L. Painting Neuropixels probes and other silicon probes for electrophysiological recordings. protocols.io https://www.protocols.io/view/painting-neuropixels-probes-and-other-silicon-prob-wxqffmw (2019).

[151] International Brain Laboratory A brain-wide map of neural activity during complex behaviour. Nature 645, 177–191 (2025). 40903598 10.1038/s41586-025-09235-0 PMC12408349

[152] International Brain Laboratory. Spike sorting pipeline for the International Brain Laboratory. Preprint at 10.6084/M9.FIGSHARE.19705522.V4 (2024).

[153] Pachitariu M. MouseLand/Kilosort: Kilosort 2.5. (Zenodo, 2021). doi:10.5281/ZENODO.4482749.

[154] Pillow J. W. Spatio-temporal correlations and visual signalling in a complete neuronal population. Nature 454, 995–999 (2008). 18650810 10.1038/nature07140 PMC2684455

[155] Park I. M., Meister M. L. R., Huk A. C. & Pillow J. W. Encoding and decoding in parietal cortex during sensorimotor decision-making. Nature Neuroscience 17, 1395–1403 (2014). 25174005 10.1038/nn.3800 PMC4176983

[156] Pillow J. W., Paninski L., Uzzell V. J., Simoncelli E. P. & Chichilnisky E. J. Prediction and decoding of retinal ganglion cell responses with a probabilistic spiking model. J. Neurosci. 25, 11003–11013 (2005). 16306413 10.1523/JNEUROSCI.3305-05.2005 PMC6725882

[157] Friedman J., Hastie T. & Tibshirani R. Regularization paths for generalized linear models via coordinate descent. J. Stat. Softw. 33, 1–22 (2010). 20808728 PMC2929880

[158] Cawley G. & Talbot N. L. C. On over-fitting in model selection and subsequent selection bias in performance evaluation. J. Mach. Learn. Res. 11, 2079–2107 (2010).

[159] Findling C. Brain-wide representations of prior information in mouse decision-making. Nature 645, 192–200 (2025). 40903597 10.1038/s41586-025-09226-1 PMC12408363

[160] Briggs F. Organizing principles of cortical layer 6. Front Neural Circuits 4, 3 (2010). 20179784 10.3389/neuro.04.003.2010 PMC2826182

[161] Madisen L. A robust and high-throughput Cre reporting and characterization system for the whole mouse brain. Nature Neuroscience 13, 133–140 (2009). 20023653 10.1038/nn.2467 PMC2840225

[162] Gerfen C. R., Paletzki R. & Heintz N. GENSAT BAC cre-recombinase driver lines to study the functional organization of cerebral cortical and basal ganglia circuits. Neuron 80, 1368–1383 (2013). 24360541 10.1016/j.neuron.2013.10.016 PMC3872013

[163] Cubelos B. Cux1 and Cux2 regulate dendritic branching, spine morphology and synapses of the upper layer neurons of the cortex. Neuron 66, 523 (2010). 20510857 10.1016/j.neuron.2010.04.038 PMC2894581

[164] Gorski J. A. Cortical excitatory neurons and glia, but not GABAergic neurons, are produced in the Emx1-expressing lineage. J Neurosci 22, 6309–6314 (2002). 12151506 10.1523/JNEUROSCI.22-15-06309.2002 PMC6758181

[165] Zhuo Y. Improved green and red GRAB sensors for monitoring dopaminergic activity in vivo. Nat. Methods 21, 680–691 (2024). 38036855 10.1038/s41592-023-02100-w PMC11009088

[166] Economo M. N. A platform for brain-wide imaging and reconstruction of individual neurons. Elife 5, e10566 (2016). 26796534 10.7554/eLife.10566 PMC4739768

[167] Ragan T. Serial two-photon tomography for automated ex vivo mouse brain imaging. Nat. Methods 9, 255–258 (2012). 22245809 10.1038/nmeth.1854 PMC3297424

[168] Wang Q. The Allen Mouse Brain Common Coordinate Framework: A 3D reference atlas. Cell 181, 936–953.e20 (2020). 32386544 10.1016/j.cell.2020.04.007 PMC8152789

[169] Klein S., Staring M., Murphy K., Viergever M. A. & Pluim J. P. W. Elastix: A toolbox for intensity-based medical image registration. IEEE Trans. Med. Imaging 29, 196–205 (2010). 19923044 10.1109/TMI.2009.2035616

[170] Liu L. D. Accurate localization of linear probe electrode arrays across multiple brains. eNeuro 8, ENEURO.0241–21.2021 (2021). 10.1523/ENEURO.0241-21.2021 PMC8597948 34697075

