# Supplementary material for: VTA dopamine neuron activity produces spatially organized value representations
Source: bioRxiv. 2025 Nov 6:2025.11.04.685995. Preprint. [Version 1] doi: 10.1101/2025.11.04.685995 (PMC12637434; doi:10.1101/2025.11.04.685995)
Supplement: Supplement 1 [file media-1.pdf]

Extended Data Tables

Model family	Model	Parameters	Model number (Fig. 1.)
Value based	Extended Q-learning model with the CS+ as reward	α_{rew} α_{stay} γ_{forget} β_{rew} β_{stay} $Bias$	1
	Extended Q-learning model with VTA DA stimulation is an RPE	α_{rew} α_{stay} γ_{forget} β_{rew} β_{stay} $Bias$	2
Policy based	REINFORCE (CS+ is the reward)	α_{win} α_{loss} β_{rew} β_{loss} $Bias$	3
	Actor-Critic (CS+ is the reward)	α_{rew} α_{win} α_{loss} β_{rew} β_{loss} $Bias$	4

Extended Data Table 1. Summary of behavioral models tested.

Parameter	Constraints	Population level prior	M, τ	Transformation
α_{rew} , α_{stay}, α_{stay}, γ_{forget}, α_{win}, α_{loss}	$\alpha, \gamma \in (0,1)$	$\tilde{\alpha} \sim N(M, \tau)$ $\tilde{\gamma} \sim N(M, \tau)$	$M = 0.5$ $\tau = 1$	$\alpha = logit^{-1}(\tilde{\alpha})$ $\gamma = logit^{-1}(\tilde{\gamma})$
β_{rew}, β_{stay}, β_{win}, β_{loss}	$\beta \in \mathbb{R}$	$\beta \sim N(M, \tau)$	$M = 0.5$ $\tau = 1$	NA
$Bias$	$Bias \in \mathbb{R}$	$Bias \sim N(M, \tau)$	$M = 0$ $\tau = 1$	NA
All standard deviations	$\sigma \in (0, \infty)$	$\tilde{\sigma} \sim N(M, \tau)$	$M = 0$ $\tau = 1$	$\sigma = exp(\tilde{\sigma})$

Extended Data Table 2. Summary of priors used for each parameter.

Allen Brain Atlas	Chon et al. 2019 Labels	Decoders Groupings	Projection Analysis Groupings
SSp-bfd5		SS	SS
SSp-bfd6a		SS	SS
SSp-bfd6b		SS	SS
SSp-n6a		SS	SS
SSp-n6b		SS	SS
SSp-un2/3		SS	SS
SSp-un4		SS	SS
SSp-un5		SS	SS
SSp-un6a		SS	SS
SSp-un6b		SS	SS
SSp-bfd1		SS	SS
SSp-bfd2/3		SS	SS
SSp-bfd4		SS	SS
SSp-ll5		SS	SS
SSp-ll6a		SS	SS
SSp-ll6b		SS	SS
SSp-m4		SS	SS
SSp-m5		SS	SS
SSp-m6a		SS	SS
SSp-m6b		SS	SS
SSp-n4		SS	SS
SSp-n5		SS	SS
SSp-ul2/3		SS	SS
SSp-ul4		SS	SS
SSp-ul5		SS	SS
SSp-ul6a		SS	SS
SSp-un6b		SS	SS
SSp-ul6b		SS	SS
SSp-n2/3		SS	SS
MOp1		MO	MO
MOp2/3		MO	MO
MOp5		MO	MO
MOp6a		MO	MO
MOp6b		MO	MO
MOs2/3		MO	MO
MOs5		MO	MO
MOs6a		MO	MO
MOs6b		MO	MO
MOs1		MO	MO

DP		PFC	PFC
ILA1		PFC	PFC
ILA2/3		PFC	PFC
ILA5		PFC	PFC
ILA6a		PFC	PFC
PL1		PFC	PFC
PL2/3		PFC	PFC
PL5		PFC	PFC
PL6a		PFC	PFC
PL6b		PFC	PFC
ACAd5		PFC	ACC
ACAd6a		PFC	ACC
ACAv2/3		PFC	ACC
ACAv5		PFC	ACC
ACAv6a		PFC	ACC
ACAd6b		PFC	ACC
ACAd2/3		PFC	ACC
ORBl1		OFC	OFC
ORBl2/3		OFC	OFC
ORBl5		OFC	OFC
ORBl6a		OFC	OFC
ORBl6b		OFC	OFC
ORBm2/3		OFC	OFC
ORBm5		OFC	OFC
ORBvl1		OFC	OFC
ORBvl2/3		OFC	OFC
ORBvl5		OFC	OFC
ORBm6a		OFC	OFC
ORBvl6a		OFC	OFC
FRP5		OFC	OFC
FRP6a		OFC	OFC
CP	Caudoputamen- rostral extreme	Rostral	
CP	Caudoputamen- rostral, medial	Rostral	
CP	Caudoputamen- rostral, intermediate, dorsal	Rostral	
CP	Caudoputamen- rostral, intermediate, ventral	Rostral	
CP	Caudoputamen- rostral, lateral, lateral strip	Rostral	
CP	Caudoputamen- rostra,l lateral, ventromedial	Rostral	
CP	Caudoputamen- intermediate, dorsomedial, dorsolateral	Intermediate	
CP	Caudoputamen- intermediate, dorsomedial, intermedial	Intermediate	

CP	Caudoputamen- intermediate, dorsomedial, central dorsal	Intermediate	
CP	Caudoputamen- intermediate, dorsomedial, dorsal tip	Intermediate	
CP	Caudoputamen- intermediate, ventromedial, ventromedial	Intermediate	
CP	Caudoputamen- intermediate, ventromedial, ventral	Intermediate	
CP	Caudoputamen- intermediate, ventromedial, central ventromedial	Intermediate	
CP	Caudoputamen- intermediate, dorsolateral, dorsal	Intermediate	
CP	Caudoputamen- intermediate, dorsolateral, intermedial dorsal	Intermediate	
CP	Caudoputamen- intermediate, ventrolateral, intermedial ventral	Intermediate	
CP	Caudoputamen- intermediate, ventrolateral, ventral	Intermediate	
CP	Caudoputamen- intermediate, ventrolateral, ventral tip	Intermediate	
CP	Caudoputamen- intermediate, ventrolateral, central ventrolateral	Intermediate	
CP	Caudoputamen- caudal, dorsal	Caudal	
CP	Caudoputamen- caudal, dorsal, dorsomedial	Caudal	
CP	Caudoputamen- caudal, dorsal, dorsolateral	Caudal	
CP	Caudoputamen- caudal, dorsal, ventromedial	Caudal	
CP	Caudoputamen- caudal, intermediate, dorsal	Caudal	
CP	Caudoputamen- caudal, intermediate, ventromedial	Caudal	
CP	Caudoputamen- caudal, intermediate, ventrolateral	Caudal	
CP	Caudoputamen- caudal, ventral	Caudal	
CP	Caudoputamen- caudal extreme	Caudal	
ACB	Accumbens nucleus, core region	NAc	
ACB	Accumbens nucleus, shell region	NAc	
ACB	Lateral accumbens, shell region	NAc	
GPe		GPe	
MA		VP	
SI		VP	

Extended Data Table 3. Group labels for recorded brain subregions.

Center of craniotomy coordinates	Probe Depth	Probe Angle	Targeted regions
1.94 mm AP, -0.76 mm ML	-4 mm DV	325/35° azimuth, 15° zenith	MO, PFC
1.84 mm AP, -2.06 mm ML	-5.12 mm DV	325/35° azimuth, 20° zenith	MO, CP, NAc, VP
2.43 mm AP, 1.72 mm ML	-4.82 mm DV	325/35° azimuth, 20° zenith	MO, OFC, NAc
0.16 mm AP, 2.58 mm ML	-4.04 mm DV	325/35° azimuth, 15° zenith	SS, CP, GPe
0.6 mm AP, 3.87 mm ML	-5.27 mm DV	90°/180° azimuth, 30° zenith	SS, CP, NAc, VP
1.6 mm AP, 0.3 mm ML	-5.37 mm DV	90°/180° azimuth, 20° zenith	PFC, NAc, VP
0.7 mm AP, 3.0 mm ML	-5.48 mm DV	90°/180° azimuth, 20° zenith	CP, NAc, VP
0.8 mm AP, -1.78 mm ML	-4.00 mm DV	90°/180° azimuth, 10° zenith	MO, CP
2.7 mm AP, -1.19 mm ML	-4.00 mm DV	90°/180° azimuth, 10° zenith	MO, OFC
1.3 mm AP, 0.3 mm ML	4.44 mm DV	90°/180° azimuth, 10° zenith	PFC, CP, NAc

Extended Data Table 4. Summary of coordinates and locations of recording targets.

Mouse Number	Mouse Name	Brain Regions Recorded	Number of recording sessions
1	dop_47	SS, MOp, CP, NAc, GPe, VP	4
2	dop_48	OFC, PFC, SS, MOs, CP, NAc, VP	4
3	dop_49	OFC, PFC, SS, MOs, CP, NAc, VP, GPe	8
4	dop_50	OFC, PFC, SS, MOs, CP, NAc, VP, GPe	6
5	dop_53	SS, MOs, NAc, CP, VP, GPe	5
6	dop_56	OFC, PFC, MO, CP, NAc, VP	5
7	dop_57	MO, CP, NAc	3
8	dop_59	PFC, MO, CP, NAc	6

Extended Data Table 5. Summary of Neuropixels recordings.

Experiment ID	Mouse line	Injection region
175372863	Wild-type	AAA
146593590	Wild-type	ACAd
112458114	Wild-type	ACAd
139426984	Wild-type	ACAd
166323896	A930038C07Rik-Tg1-Cre	ACAd
286774770	A930038C07Rik-Tg1-Cre	ACAd
171275617	A930038C07Rik-Tg1-Cre	ACAd
125833030	Rbp4-Cre_KL100	ACAd
496576666	Rbp4-Cre_KL100	ACAd
526783054	Rbp4-Cre_KL100	ACAd
293432575	Tlx3-Cre_PL56	ACAd
293431869	Tlx3-Cre_PL56	ACAd
539521918	Cux2-IRES-Cre	ACAd
522774029	Cux2-IRES-Cre	ACAd
583747816	Emx1-IRES-Cre	ACAd
478491090	Emx1-IRES-Cre	ACAd
478376911	Emx1-IRES-Cre	ACAd
584194481	Emx1-IRES-Cre	ACAd
501483880	Emx1-IRES-Cre	ACAd
183472596	Grp-Cre_KH288	ACAd
112514202	Wild-type	ACAv
139520203	Wild-type	ACAv
159319654	A930038C07Rik-Tg1-Cre	ACAv
298797288	A930038C07Rik-Tg1-Cre	ACAv
286482701	Rbp4-Cre_KL100	ACAv
161458737	Rbp4-Cre_KL100	ACAv
126117554	Rbp4-Cre_KL100	ACAv

294434161	Cux2-IRES-Cre	ACAv
571823196	Cux2-IRES-Cre	ACAv
183470468	Cux2-IRES-Cre	ACAv
183461297	Cux2-IRES-Cre	ACAv
265288825	Grp-Cre_KH288	ACAv
265291552	Grp-Cre_KH288	ACAv
184213701	Grp-Cre_KH288	ACAv
266583498	Grp-Cre_KH288	ACAv
112596790	Wild-type	AId
272737914	Wild-type	AId
267397941	A930038C07Rik-Tg1-Cre	AId
296048512	Rbp4-Cre_KL100	AId
294525229	Tlx3-Cre_PL56	AId
299783689	Cux2-IRES-Cre	AId
296047806	Cux2-IRES-Cre	AId
264076854	Cux2-IRES-Cre	AId
184168899	Grp-Cre_KH288	AId
286775476	A930038C07Rik-Tg1-Cre	AIp
183330908	Rbp4-Cre_KL100	AIp
272827141	Cux2-IRES-Cre	AIp
166153483	Rbp4-Cre_KL100	AIv
113144533	Wild-type	BLA
120282354	Wild-type	BLA
277710753	Wild-type	BLA
113935990	Wild-type	BMA
115958115	Wild-type	BMA
125832322	Wild-type	BMA
146012934	Wild-type	BMA

273028004	Wild-type	BMA
127761449	Wild-type	CEA
146795148	Wild-type	CEA
112459547	Wild-type	CEA
180404418	Wild-type	GU
292375491	A930038C07Rik-Tg1-Cre	GU
249402048	Rbp4-Cre_KL100	GU
516846757	Cux2-IRES-Cre	LA
100141780	Wild-type	MOp
180720175	Wild-type	MOp
127084296	Wild-type	MOp
584903636	Wild-type	MOp
126909424	Wild-type	MOp
100141273	Wild-type	MOp
100141563	Wild-type	MOp
272697944	Wild-type	MOp
166461193	A930038C07Rik-Tg1-Cre	MOp
287461719	A930038C07Rik-Tg1-Cre	MOp
166082842	Rbp4-Cre_KL100	MOp
591612976	Tlx3-Cre_PL56	MOp
182616478	Cux2-IRES-Cre	MOp
180709230	Wild-type	ORBl
112306316	Wild-type	ORBl
170721670	Wild-type	ORBl
180673746	Wild-type	ORBl
168164972	A930038C07Rik-Tg1-Cre	ORBl
156741826	Rbp4-Cre_KL100	ORBl
183471174	Cux2-IRES-Cre	ORBl

288324211	Grp-Cre_KH288	ORBl
112423392	Wild-type	ORBvl
158435116	Wild-type	ORBvl
167902586	Rbp4-Cre_KL100	ORBvl
287769286	Rbp4-Cre_KL100	ORBvl
480994108	Rbp4-Cre_KL100	ORBvl
293433283	Tlx3-Cre_PL56	ORBvl
522773270	Cux2-IRES-Cre	ORBvl
266564027	Grp-Cre_KH288	ORBvl
157711748	Wild-type	PL
292374777	A930038C07Rik-Tg1-Cre	PL
263106036	Rbp4-Cre_KL100	PL
545434921	Tlx3-Cre_PL56	PL
278433737	Cux2-IRES-Cre	PL
287494320	Cux2-IRES-Cre	PL
294396492	Cux2-IRES-Cre	PL
478376197	Emx1-IRES-Cre	PL
266582782	Grp-Cre_KH288	PL
112951804	Wild-type	SSp-bfd
126907302	Wild-type	SSp-bfd
100142655	Wild-type	SSp-bfd
127866392	Wild-type	SSp-bfd
112882565	Wild-type	SSp-bfd
100141473	Wild-type	SSp-bfd
182467026	A930038C07Rik-Tg1-Cre	SSp-bfd
177907797	A930038C07Rik-Tg1-Cre	SSp-bfd
643749624	A930038C07Rik-Tg1-Cre	SSp-bfd
644250774	A930038C07Rik-Tg1-Cre	SSp-bfd

647806688	Rbp4-Cre_KL100	SSp-bfd
648253235	Rbp4-Cre_KL100	SSp-bfd
511550172	Rbp4-Cre_KL100	SSp-bfd
272735030	Rbp4-Cre_KL100	SSp-bfd
183171679	Rbp4-Cre_KL100	SSp-bfd
297654263	Tlx3-Cre_PL56	SSp-bfd
288170549	Tlx3-Cre_PL56	SSp-bfd
670228985	Tlx3-Cre_PL56	SSp-bfd
560736273	Cux2-IRES-Cre	SSp-bfd
168003640	Cux2-IRES-Cre	SSp-bfd
266486371	Cux2-IRES-Cre	SSp-bfd
294435580	Cux2-IRES-Cre	SSp-bfd
583748537	Emx1-IRES-Cre	SSp-bfd
657041814	Emx1-IRES-Cre	SSp-bfd
112791318	Wild-type	SSp-ll
112935169	Wild-type	SSp-ll
114292355	Wild-type	SSp-ll
166323186	A930038C07Rik-Tg1-Cre	SSp-ll
264629246	A930038C07Rik-Tg1-Cre	SSp-ll
272698650	Rbp4-Cre_KL100	SSp-ll
297652799	Rbp4-Cre_KL100	SSp-ll
182803137	Rbp4-Cre_KL100	SSp-ll
591622344	Tlx3-Cre_PL56	SSp-ll
296052839	Tlx3-Cre_PL56	SSp-ll
584902900	Tlx3-Cre_PL56	SSp-ll
298830161	Cux2-IRES-Cre	SSp-ll
266644610	Cux2-IRES-Cre	SSp-ll
178284661	Cux2-IRES-Cre	SSp-ll

180717881	Wild-type	SSp-m
157654817	Wild-type	SSp-m
112936582	Wild-type	SSp-m
114290938	Wild-type	SSp-m
100149969	Wild-type	SSp-m
298796577	A930038C07Rik-Tg1-Cre	SSp-m
272928602	Rbp4-Cre_KL100	SSp-m
303785454	Rbp4-Cre_KL100	SSp-m
303614706	Tlx3-Cre_PL56	SSp-m
294397199	Cux2-IRES-Cre	SSp-m
278317945	Cux2-IRES-Cre	SSp-m
126908007	Wild-type	SSp-n
112162251	Wild-type	SSp-n
112373124	Wild-type	SSp-n
298326521	A930038C07Rik-Tg1-Cre	SSp-n
171276330	A930038C07Rik-Tg1-Cre	SSp-n
120875816	Rbp4-Cre_KL100	SSp-n
249327301	Rbp4-Cre_KL100	SSp-n
126852363	Wild-type	SSp-tr
100141495	Wild-type	SSp-tr
281459203	A930038C07Rik-Tg1-Cre	SSp-tr
298182842	Tlx3-Cre_PL56	SSp-tr
180718587	Wild-type	SSp-ul
112229814	Wild-type	SSp-ul
148964212	Wild-type	SSp-ul
292792016	A930038C07Rik-Tg1-Cre	SSp-ul
266249483	Rbp4-Cre_KL100	SSp-ul
286312782	Rbp4-Cre_KL100	SSp-ul

303615412	Tlx3-Cre_PL56	SSp-ul
298106713	Cux2-IRES-Cre	SSp-ul
286300594	Cux2-IRES-Cre	SSp-ul
180601025	Wild-type	SSp-un
112514915	Wild-type	SSs
117298988	Wild-type	SSs
174360333	Wild-type	SSs
156395997	A930038C07Rik-Tg1-Cre	SSs
286834976	A930038C07Rik-Tg1-Cre	SSs
168163498	Rbp4-Cre_KL100	SSs
298179622	Rbp4-Cre_KL100	SSs
120916102	Rbp4-Cre_KL100	SSs
286314623	Rbp4-Cre_KL100	SSs
303616833	Tlx3-Cre_PL56	SSs
288171256	Tlx3-Cre_PL56	SSs
297855879	Cux2-IRES-Cre	SSs
287951098	Cux2-IRES-Cre	SSs
577773267	Cux2-IRES-Cre	SSs

Extended Data Table 6. Summary of Allen Projection Data injections analyzed in Fig. 3l–m.

Figure	Description	Test	*P*-value	Test statistic	Sample size
Fig. 2g	Action value encoding across striatal subregions (proportion)	χ^2 test	• CP-Int vs. NAc: P = 1.2e-12<br>• CP-Int vs. CP-Ros: P = 2.6e-8<br>• CP-Int vs. CP-Cau: P = 0.054<br><br>• NAc vs. CP-Ros: P = 0.0072<br>• NAc vs. CP-Cau: P = 1.1e-6	• CP-Int vs. NAc: χ^2 = 50.44<br>• CP-Int vs. CP-Ros: χ^2 = 30.97<br>• CP-Int vs. CP-Cau: χ^2 = 3.73<br><br>• NAc vs. CP-Ros: χ^2 = 7.21<br>• NAc vs. CP-Cau: χ^2 = 23.80	NAc: n = 829<br><br>CP-Ros: n = 1529<br><br>CP-Int: n = 958<br><br>CP-Cau: n = 720
Ext. Data Fig. 3e	Firing rates in intermediate CP vs. NAc	Mann–Whitney U test (normal approximation)	• CP-Int vs. NAc: P = 9.3e-4	• CP-Int vs. NAc: Z = -3.31	NAc: n = 829<br><br>CP-Int: n = 958
Ext. Data Fig. 4a	Action value encoding across striatal subregions (ΔR^2)	Mann–Whitney U test (normal approximation)	• CP-Int vs. CP-Cau: P = 3.5e-7	• CP-Int vs. CP-Cau: Z = 5.09	CP-Int: n = 958<br><br>CP-Cau: n = 720
Fig. 2h	Action value encoding in cortex vs. intermediate CP (proportion)	χ^2 test	• CP-Int vs. OFC: P = 7.4e-9<br>• CP-Int vs. PFC: P = 1.5e-5<br>• CP-Int vs. MO: P = 1.0e-7<br>• CP-Int vs. SS: P = 0.0039	• CP-Int vs. OFC: χ^2 = 33.42<br>• CP-Int vs. PFC: χ^2 = 18.79<br>• CP-Int vs. MO: χ^2 = 28.34<br>• CP-Int vs. SS: χ^2 = 8.35	CP-Int: n = 958<br><br>OFC: n = 733<br><br>PFC: n = 996<br><br>MO: n = 1088<br><br>SS: n = 402
Fig. 2h	Action value encoding in pallidum vs.	χ^2 test	• CP-Int vs. VP: P = 0.038	• CP-Int vs. VP: χ^2 = 4.32	CP-Int: n = 958

	intermediate CP (proportion)		• CP-Int vs. GPe: *P* = 0.51	• CP-Int vs. GPe: χ^2 = 0.43	VP: *n* = 522<br><br>GPe: *n* = 615
Fig. 2i	Action identity encoding in MO vs. other regions (proportion)	χ^2 test	• CP-Int vs. MO: *P* = 0.0012<br><br>• MO vs. OFC: *P* < 1e-16<br>• MO vs. PFC: *P* < 1e-16<br>• MO vs. SS: *P* = 2.9e-8	• CP-Int vs. MO: χ^2 = 10.46<br><br>• MO vs. OFC: χ^2 = 132.57<br>• MO vs. PFC: χ^2 = 120.31<br>• MO vs. SS: χ^2 = 30.77	CP-Int: *n* = 958<br><br>OFC: *n* = 733<br><br>PFC: *n* = 996<br><br>MO: *n* = 1088<br><br>SS: *n* = 402
Fig. 2k-n	Action value decoding in CP vs NAc	Mann–Whitney U tests	*Contralateral trials*:<br>• Q^{contra} CP vs NAc (time bin, s) :<br>○ -0.4 : P = 0.238<br>○ -0.3 : P = 0.73<br>○ -0.2 : P = 0.074<br>○ -0.1 : P = 0.15<br>○ 0.0 : P = 0.02<br>○ 0.1 : P = 0.034<br>○ 0.2 : P = 0.047<br>○ 0.3 : P = 0.026<br>○ 0.4 : P = 0.044<br>○ 0.5 : P = 0.007<br>• Q^{ipsi} CP vs NAc :<br>○ -0.4 : P = 0.131<br>○ -0.3 : P = 0.025<br>○ -0.2 : P = 0.645<br>○ -0.1 : P = 0.463<br>○ -0.0 : P = 0.006<br>○ 0.1 : P = 0.158<br>○ 0.2 : P = 0.029<br>○ 0.3 : P = 0.127<br>○ 0.4 : P = 0.472<br>○ 0.5 : P = 0.092<br>*Ipsilateral trials:*	*Contralateral trials*:<br>• Q^{contra} CP vs NAc (time bin, s) :<br>○ -0.4 : U = -1.181<br>○ -0.3 : U = -0.346<br>○ -0.2 : U = -1.785<br>○ -0.1 : U = -1.44<br>○ 0.0 : U = -2.332<br>○ 0.1 : U = -2.116<br>○ 0.2 : U = -1.987<br>○ 0.3 : U = -2.232<br>○ 0.4 : U = -2.016<br>○ 0.5 : U = -2.678<br>• Q^{ipsi} CP vs NAc :<br>○ -0.4 : U = -1.512<br>○ -0.3 : U = -2.246<br>○ -0.2 : U = -0.461<br>○ -0.1 : U = -0.734<br>○ -0.0 : U = -2.764<br>○ 0.1 : U = -1.411<br>○ 0.2 : U = -2.188<br>○ 0.3 : U = -1.526<br>○ 0.4 : U = -0.72<br>○ 0.5 : U = -1.685<br>*Ipsilateral trials:*	NAc: n=18<br>CP: n=48

			• Q^{contra} CP vs NAc (time bin, s) :<br>○ -0.4 : P = 0.167<br>○ -0.3 : P = 0.142<br>○ -0.2 : P = 0.954<br>○ -0.1 : P = 0.32<br>○ -0.0 : P = 0.73<br>○ 0.1 : P = 0.404<br>○ 0.2 : P = 0.037<br>○ 0.3 : P = 0.314<br>○ 0.4 : P = 0.584<br>○ 0.5 : P = 0.614<br>• Q^{ipsi} CP vs NAc (time bin, s):<br>○ -0.4 : P = 0.195<br>○ -0.3 : P = 0.645<br>○ -0.2 : P = 0.584<br>○ -0.1 : P = 0.604<br>○ -0.0 : P = 0.227<br>○ 0.1 : P = 0.977<br>○ 0.2 : P = 0.21<br>○ 0.3 : P = 0.205<br>○ 0.4 : P = 0.404<br>○ 0.5 : P = 0.666	• Q^{contra} CP vs NAc (time bin, s) :<br>○ -0.4 : U = -1.382<br>○ -0.3 : U = -1.469<br>○ -0.2 : U = -0.058<br>○ -0.1 : U = -0.993<br>○ -0.0 : U = -0.346<br>○ 0.1 : U = -0.835<br>○ 0.2 : U = -2.088<br>○ 0.3 : U = -1.008<br>○ 0.4 : U = -0.547<br>○ 0.5 : U = 0.504<br>• Q^{ipsi} CP vs NAc (time bin, s):<br>○ -0.4 : U = -1.296<br>○ -0.3 : U = -0.461<br>○ -0.2 : U = -0.547<br>○ -0.1 : U = -0.518<br>○ -0.0 : U = -1.209<br>○ 0.1 : U = -0.029<br>○ 0.2 : U = -1.253<br>○ 0.3 : U = -1.267<br>○ 0.4 : U = -0.835<br>○ 0.5 : U = 0.432	
Fig. 2k-n	Action value decoding of chosen vs unchosen actions	Wilcoxon signed ranked test	*CP*:<br>• Q^{contra} at contra- trials vs Q^{ipsi} at contra- trials (time bin, s):<br>○ -0.4 : P = 0.593<br>○ -0.3 : P = 0.336<br>○ -0.2 : P = 0.016<br>○ -0.1 : P = 0.255<br>○ -0.0 : P = 0.001<br>○ 0.1 : P = 0.007<br>○ 0.2 : P = 0.0<br>○ 0.3 : P = 0.0<br>○ 0.4 : P = 0.002<br>○ 0.5 : P = 0.003<br>• Q^{ipsi} at ipsi- trials vs Q^{contra} at ipsi- trials (time bin, s):<br>○ -0.4 : P = 0.572	*CP*:<br>• Q^{contra} at contra- trials vs Q^{ipsi} at contra- trials (time bin, s):<br>○ -0.4 : W = 535.0<br>○ -0.3 : W = 493.0<br>○ -0.2 : W = 354.0<br>○ -0.1 : W = 476.0<br>○ -0.0 : W = 260.0<br>○ 0.1 : W = 328.0<br>○ 0.2 : W = 241.0<br>○ 0.3 : W = 203.0<br>○ 0.4 : W = 287.0<br>○ 0.5 : W = 301.0<br>• Q^{ipsi} at ipsi- trials vs Q^{contra} at ipsi- trials (time bin, s):<br>○ -0.4 : W = 532.0	NAc: n=18<br>CP:<br>n=48

			○ -0.3 : P = 0.923<br>○ -0.2 : P = 0.306<br>○ -0.1 : P = 0.741<br>○ -0.0 : P = 0.615<br>○ 0.1 : P = 0.134<br>○ 0.2 : P = 0.019<br>○ 0.3 : P = 0.001<br>○ 0.4 : P = 0.0<br>○ 0.5 : P = 0.039<br>*<u>NAc:</u>*<br>● Q^{contra} at contra- trials vs Q^{ipsi} at contra- trials (time bin, s):<br>○ -0.4 : P = 0.734<br>○ -0.3 : P = 0.325<br>○ -0.2 : P = 0.551<br>○ -0.1 : P = 0.671<br>○ -0.0 : P = 0.265<br>○ 0.1 : P = 0.609<br>○ 0.2 : P = 0.108<br>○ 0.3 : P = 0.246<br>○ 0.4 : P = 0.832<br>○ 0.5 : P = 1.0<br>● Q^{ipsi} at ipsi- trials vs Q^{contra} at ipsi- trials (time bin, s):<br>○ -0.4 : P = 0.966<br>○ -0.3 : P = 0.495<br>○ -0.2 : P = 0.832<br>○ -0.1 : P = 1.0<br>○ -0.0 : P = 0.265<br>○ 0.1 : P = 0.108<br>○ 0.2 : P = 0.06<br>○ 0.3 : P = 0.119<br>○ 0.4 : P = 0.048<br>○ 0.5 : P = 0.142	○ -0.3 : W = 578.0<br>○ -0.2 : W = 487.0<br>○ -0.1 : W = 555.0<br>○ -0.0 : W = 538.0<br>○ 0.1 : W = 441.0<br>○ 0.2 : W = 361.0<br>○ 0.3 : W = 270.0<br>○ 0.4 : W = 242.0<br>○ 0.5 : W = 387.0<br>*<u>NAc:</u>*<br>● Q^{contra} at contra- trials vs Q^{ipsi} at contra- trials (time bin, s):<br>○ -0.4 : W = 77.0<br>○ -0.3 : W = 62.0<br>○ -0.2 : W = 71.0<br>○ -0.1 : W = 75.0<br>○ -0.0 : W = 59.0<br>○ 0.1 : W = 73.0<br>○ 0.2 : W = 48.0<br>○ 0.3 : W = 58.0<br>○ 0.4 : W = 80.0<br>○ 0.5 : W = 85.0<br>● Q^{ipsi} at ipsi- trials vs Q^{contra} at ipsi- trials (time bin, s):<br>○ -0.4 : W = 84.0<br>○ -0.3 : W = 69.0<br>○ -0.2 : W = 80.0<br>○ -0.1 : W = 85.0<br>○ -0.0 : W = 59.0<br>○ 0.1 : W = 48.0<br>○ 0.2 : W = 42.0<br>○ 0.3 : W = 49.0<br>○ 0.4 : W = 40.0<br>○ 0.5 : W = 51.0	
Fig. 3d	Relative value encoding across striatal subregions (proportion)	χ^2 test	● CP-Int vs. NAc: *P* = 1.6e-14<br>● CP-Int vs. CP-Ros: *P* = 4.9e-9<br>● CP-Int vs. CP-Cau: *P* = 0.0048	● CP-Int vs. NAc: χ^2 = 59.03<br>● CP-Int vs. CP-Ros: χ^2 = 34.23	NAc: *n* = 829<br><br>CP-Ros: *n* = 1529<br><br>CP-Int:

			• NAc vs. CP-Ros: *P* = 0.00061<br>• NAc vs. CP-Cau: *P* = 1.2e-6	• CP-Int vs. CP-Cau: χ^2 = 7.97<br><br>• NAc vs. CP-Ros: χ^2 = 11.76<br>• NAc vs. CP-Cau: χ^2 = 23.65	*n* = 958<br><br>CP-Cau: *n* = 720
Fig. 3e	State value encoding across striatal subregions (proportion)	χ^2 test	• CP-Int vs. NAc: *P* = 9.1e-6<br>• CP-Int vs. CP-Ros: *P* = 0.0085<br>• CP-Int vs. CP-Cau: *P* = 0.67<br><br>• NAc vs. CP-Ros: *P* = 0.016<br>• NAc vs. CP-Cau: *P* = 4.3e-6	• CP-Int vs. NAc: χ^2 = 19.70<br>• CP-Int vs. CP-Ros: χ^2 = 6.92<br>• CP-Int vs. CP-Cau: χ^2 = 0.18<br><br>• NAc vs. CP-Ros: χ^2 = 5.80<br>• NAc vs. CP-Cau: χ^2 = 21.11	NAc: *n* = 829<br><br>CP-Ros: *n* = 1529<br><br>CP-Int: *n* = 958<br><br>CP-Cau: *n* = 720
Fig. 3f	Relative value decoding in CP vs NAc	Mann–Whitney U test	• CP vs NAc (time bin, s):<br>○ -0.4 : P =0.025<br>○ -0.3 : P = 0.852<br>○ -0.2 : P = 0.084<br>○ -0.1 : P = 0.335<br>○ 0.0 : P = 0.205<br>○ 0.1 : P = 0.001<br>○ 0.2 : P = 0.017<br>○ 0.3 : P = 0.089<br>○ 0.4 : P = 0.146<br>○ 0.5 : P = 0.007	• CP vs NAc (time bin, s):<br>○ -0.4 : U = -2.246<br>○ -0.3 : U = -0.187<br>○ -0.2 : U = -1.728<br>○ -0.1 : U = -0.965<br>○ 0.0 : U = -1.267<br>○ 0.1 : U = -3.340<br>○ 0.2 : U = -2.39<br>○ 0.3 : U = -1.699<br>○ 0.4 : U = -1.454<br>○ 0.5 : U = -2.721	NAc: n=18<br>CP: n=48

Fig. 3g	State value decoding in CP vs NAc	Mann–Whitney U test	● CP vs NAc (time bin, s) :<br>○ -0.4 : P = 0.002<br>○ -0.3 : P = 0.023<br>○ -0.2 : P = 0.001<br>○ -0.1 : P = 0.001<br>○ -0.0 : P = 0.003<br>○ 0.1 : P = 0.001<br>○ 0.2 : P = 0.001<br>○ 0.3 : P = 0.044<br>○ 0.4 : P = 0.142<br>○ 0.5 : P = 0.023<br>○ 0.6 : P = 0.216<br>○ 0.7 : P = 0.079<br>○ 0.8 : P = 0.205<br>○ 0.9 : P = 0.773<br>○ 1.0 : P = 0.176<br>○ 1.1 : P = 0.545<br>○ 1.2 : P = 0.852<br>○ 1.3 : P = 0.146<br>○ 1.4 : P = 0.943<br>○ 1.5 : P = 0.472<br>○ 1.6 : P = 0.349<br>○ 1.7 : P = 0.146<br>○ 1.8 : P = 0.238<br>○ 1.9 : P = 0.04<br>○ 2.0 : P = 0.009	● CP vs NAc (time bin, s) :<br>○ -0.4 : U = -3.067<br>○ -0.3 : U = -2.275<br>○ -0.2 : U = -3.225<br>○ -0.1 : U = -3.297<br>○ -0.0 : U = -2.952<br>○ 0.1 : U = -3.24<br>○ 0.2 : U = -3.47<br>○ 0.3 : U = -2.016<br>○ 0.4 : U = -1.469<br>○ 0.5 : U = -2.275<br>○ 0.6 : U = -1.238<br>○ 0.7 : U = -1.757<br>○ 0.8 : U = -1.267<br>○ 0.9 : U = -0.288<br>○ 1.0 : U = -1.353<br>○ 1.1 : U = -0.605<br>○ 1.2 : U = -0.187<br>○ 1.3 : U = -1.454<br>○ 1.4 : U = -0.072<br>○ 1.5 : U = -0.72<br>○ 1.6 : U = -0.936<br>○ 1.7 : U = -1.454<br>○ 1.8 : U = -1.181<br>○ 1.9 : U = -2.059<br>○ 2.0 : U = -2.62	NAc: n = 18<br>CP:<br>n = 48
Fig. 3j	Encoding of relative vs. state value along the medial/lateral and dorsal/ventral axes of intermediate CP (proportion)	χ^2 test	Medial/lateral bins:<br>○ 1.00–1.25 mm: *P* = 2.5e-6<br>○ 1.25–1.50 mm: *P* = 1.3e–7<br>○ 1.50–1.75 mm: *P* = 4.4e-4<br>○ 1.75–2.00 mm: *P* = 4.8e-6<br>○ 2.00–2.25 mm: *P* = 0.90<br>○ 2.25–2.50 mm: *P* = 0.63<br>○ 2.50–2.75 mm: *P* = 1.00<br>○ 2.75–3.00 mm: *P* = 0.40<br><br>Dorsal/ventral bins:	Medial/lateral bins:<br>○ 1.00–1.25 mm: χ^2 = 22.19<br>○ 1.25–1.50 mm: χ^2 = 27.94<br>○ 1.50–1.75 mm: χ^2 = 12.34<br>○ 1.75–2.00 mm: χ^2 = 20.93<br>○ 2.00–2.25 mm: χ^2 = 0.02<br>○ 2.25–2.50 mm: χ^2 = 0.24<br>○ 2.50–2.75 mm: χ^2 = 0.00<br>○ 2.75–3.00 mm: χ^2 = 0.70	Medial/lateral bins:<br>○ 1.00–1.25 mm: *n* = 61<br>○ 1.25–1.50 mm: *n* = 130<br>○ 1.50–1.75 mm: *n* = 42<br>○ 1.75–2.00 mm: *n* = 147<br>○ 2.00–2.25 mm: *n* = 168

			○ 2.25–2.75 mm: *P* = 1.00<br>○ 2.75–3.25 mm: *P* = 0.35<br>○ 3.25–3.75 mm: *P* = 0.022<br>○ 3.75–4.25 mm: *P* = 0.093<br>○ 4.25–4.75 mm: *P* = 0.10<br>○ 4.75–5.25 mm: *P* = 8.5e-9<br>○ 5.25–5.75 mm: *P* = 3.5e-4	Dorsal/ventral bins:<br>○ 2.25–2.75 mm: χ^2 = 0.00<br>○ 2.75–3.25 mm: χ^2 = 0.88<br>○ 3.25–3.75 mm: χ^2 = 5.21<br>○ 3.75–4.25 mm: χ^2 = 2.82<br>○ 4.25–4.75 mm: χ^2 = 2.67<br>○ 4.75–5.25 mm: χ^2 = 33.17<br>○ 5.25–5.75 mm: χ^2 = 12.76	○ 2.25–2.50 mm: *n* = 211<br>○ 2.50–2.75 mm: *n* = 126<br>○ 2.75–3.00 mm: *n* = 68<br><br>Dorsal/ventral bins:<br>○ 2.25–2.75 mm: *n* = 22<br>○ 2.75–3.25 mm: *n* = 117<br>○ 3.25–3.75 mm: *n* = 221<br>○ 3.75–4.25 mm: *n* = 176<br>○ 4.25–4.75 mm: *n* = 243<br>○ 4.75–5.25 mm: *n* = 155<br>○ 5.25–5.75 mm: *n* = 24
Fig. 3k	Correlation of relative and state value striatal domain encoding with cortical projection intensity	Pearson correlation	Relative Value:<br>○ PFC: P = 0.002<br>○ ACC: P = 0.016<br>○ OFC: P = 0.153<br>○ Insula: P = 0.677<br>○ SS: P = 0.122<br>○ MO: P = 2.442e-05<br>State Value :<br>○ PFC: P = 0.225<br>○ ACC: P = 0.814<br>○ OFC: P = 0.071<br>○ Insula: P = 0.902<br>○ SS: P = 0.279	Relative Value:<br>○ PFC: r = -0.592<br>○ ACC: r = -0.485<br>○ OFC: r = -0.294<br>○ Insula: r = 0.099<br>○ SS: r = 0.337<br>○ MO: r = 0.744<br>State Value :<br>○ PFC: r = 0.259<br>○ ACC: r = 0.05<br>○ OFC: r =0.373<br>○ Insula: r = -0.022	n = 24 striatal domains sampled above threshold (n = 20 neurons)

			○ MO: P = 0.894	○ SS: r = -0.244<br>○ MO: r =- 0.025	
Ext. Data Fig. 7e	State value encoding across striatal subregions (ΔR^2)	Mann–Whitney U test (normal approximation)	• CP-Int vs. CP-Cau: *P* = 0.0017	• CP-Int vs. CP-Cau: *Z* = 3.13	CP-Int: *n* = 958<br><br>CP-Cau: *n* = 720
Ext. Data Fig. 7m	Encoding of relative vs. state value along the medial/lateral and dorsal/ventral axes of intermediate CP (ΔR^2)	Mann–Whitney U test (normal approximation)	Medial/lateral bins:<br>○ 1.00–1.25 mm: *P* = 4.5e-9<br>○ 1.25–1.50 mm: *P* = 2.2e-10<br>○ 1.50–1.75 mm: *P* = 3.5e-4<br>○ 1.75–2.00 mm: *P* = 3.3e-12<br>○ 2.00–2.25 mm: *P* = 0.66<br>○ 2.25–2.50 mm: *P* = 0.38<br>○ 2.50–2.75 mm: *P* = 0.36<br>○ 2.75–3.00 mm: *P* = 0.47<br><br>Dorsal/ventral bins:<br>○ 2.25–2.75 mm: *P* = 0.77<br>○ 2.75–3.25 mm: *P* = 0.11<br>○ 3.25–3.75 mm: *P* = 0.077<br>○ 3.75–4.25 mm: *P* = 0.021<br>○ 4.25–4.75 mm: *P* = 1.3e-5	Medial/lateral bins:<br>○ 1.00–1.25 mm: *Z* = 5.86<br>○ 1.25–1.50 mm: *Z* = 6.35<br>○ 1.50–1.75 mm: *Z* = 3.57<br>○ 1.75–2.00 mm: *Z* = 6.96<br>○ 2.00–2.25 mm: *Z* = 0.44<br>○ 2.25–2.50 mm: *Z* = 0.89<br>○ 2.50–2.75 mm: *Z* = 0.91<br>○ 2.75–3.00 mm: *Z* = 0.73<br><br>Dorsal/ventral bins:<br>○ 2.25–2.75 mm: *Z* = 0.29<br>○ 2.75–3.25 mm: *Z* = 1.59<br>○ 3.25–3.75 mm: *Z* = 1.77<br>○ 3.75–4.25 mm: *Z* = 2.30<br>○ 4.25–4.75 mm: *Z* = 4.35	Medial/lateral bins:<br>○ 1.00–1.25 mm: *n* = 61<br>○ 1.25–1.50 mm: *n* = 130<br>○ 1.50–1.75 mm: *n* = 42<br>○ 1.75–2.00 mm: *n* = 147<br>○ 2.00–2.25 mm: *n* = 168<br>○ 2.25–2.50 mm: *n* = 211<br>○ 2.50–2.75 mm: *n* = 126<br>○ 2.75–3.00 mm: *n* = 68<br><br>Dorsal/ventral bins:

			○ 4.75–5.25 mm: *P* = 5.9e-13<br>○ 5.25–5.75 mm: *P* = 2.1e-4	○ 4.75–5.25 mm: *Z* = 7.20<br>○ 5.25–5.75 mm: *Z* = 3.70	○ 2.25–2.75 mm: *n* = 22<br>○ 2.75–3.25 mm: *n* = 117<br>○ 3.25–3.75 mm: *n* = 221<br>○ 3.75–4.25 mm: *n* = 176<br>○ 4.25–4.75 mm: *n* = 243<br>○ 4.75–5.25 mm: *n* = 155<br>○ 5.25–5.75 mm: *n* = 24
Fig. 5e	Correlation between the fraction of uncollected VTA stimulations (VTA stimulation/ CS+ obtained) and overall performance (CS+ obtained/trial number) at the session level	Linear mixed effects model with mouse and session nested random effects	CS+ rate (Performance) fixed Effect: P = 0.0002	CS+ rate (Performance) fixed Effect: t = 4.082	n = 41 sessions, 8 mice
Fig. 5f	Average return probability as a function of	Wilcoxon signed ranked test with fdr-bh multiple	• ‘CS+ w/o VTA stim’ vs ‘CS–’: P = 0.0002<br>• ‘CS+ w/o VTA stim’ vs ‘CS+ w/	• ‘CS+ w/o VTA stim’ vs ‘CS–’: W = 26<br>• ‘CS+ w/o VTA stim’ vs ‘CS+ w/	n = 24 sessions, 7 mice

	trial outcome	comparison correction	VTA stim': P = 0.095<br>● 'CS+ w/VTA stim' vs 'CS–': P = 7.152e-07	VTA stim': W = 91<br>● 'CS+ w/VTA stim' vs 'CS–': W = 1	
Fig. 5h	Performance comparison with and without CS+	Wilcoxon signed ranked test	'With CS+' vs 'Without CS+' :<br>● Reward Rate : P = 0.312<br>● p(High) : P = 0.312<br>● $Bias$: P = 0.031<br>● α_{rew}: P = 0.562<br>● α_{stay}: P = 0.031<br>● γ_{forget}: P = 0.438	'With CS+' vs 'Without CS+' :<br>● Reward Rate: W = 0.0<br>● p(High) : W = 0.0<br>● $Bias$: W = 0.0<br>● α_{rew} : W = 7.0<br>● α_{stay} : W = 0.0<br>● γ_{forget} : W = 6.0	n = 6 mice
Fig. 5h	Performance comparison with and without CS+ (normally distributed parameters)	Paired t-test	'With CS+' vs 'Without CS+' :<br>● β_{rew} : P = 0.036<br>● β_{stay} : P = 0.479	'With CS+' vs 'Without CS+' :<br>● β_{rew} : T = 2.838<br>● β_{stay} : T = -0.765	n = 6 mice

Extended Data Table 7. Summary of statistical comparisons.
